# Supplementary material for: Luteolin detoxifies DEHP and prevents liver injury by degrading Uroc1 protein in mice
Source: EMBO Mol Med. 2024 Oct 29;16(11):2699–724. doi: 10.1038/s44321-024-00160-9 (PMC11555401; doi:10.1038/s44321-024-00160-9)
Supplement: Supplementary file 1 — Appendix [file 44321_2024_160_MOESM1_ESM.pdf]

Appendix

Luteolin detoxifies DEHP and prevents liver injury by degrading Uroc1 protein in mice

Table of contents

Appendix Figure S1. Network pharmacology analysis for the novel drugs possessing the abilities for the hepatic removal of excess DEHP 2

Appendix Figure S2. Validation of a long-term stability of the *csa*-DEHP system stored at -80℃ for over 1 year 3

Appendix Figure S3. Luteolin is a potential drug to decease DEHP accumulation in the mouse liver. 4

Appendix Figure S4. *Trans*-UCA serves as the functional metabolite mimicking the luteolin. 5

Appendix Table S1. Numbers of active ingredients from 21 Chinese medicines..........6

Appendix Table S2. Topological calculations of effective chemical compounds..........7

Appendix Table S3. Sequences of candidate aptamers targeting DEHP....................8-9

Appendix Table S4. MOE analysis of binding potentials of luteolin on differentially expressed proteins...10

Appendix Table S5. The sequences of ssDNA nucleotides...11

Appendix Table S6. Capture-SELEX screening condition...12

Appendix Table S7. Mobile phase time table...13

Appendix Table S8. ESI ion source Settings..14

Appendix Table S9. Scan segments...15

Appendix Table S10. Lists of primer sequences for qPCR analysis............................16

Appendix Table S11. Exact *P* values...17-21

Appendix Figure S1

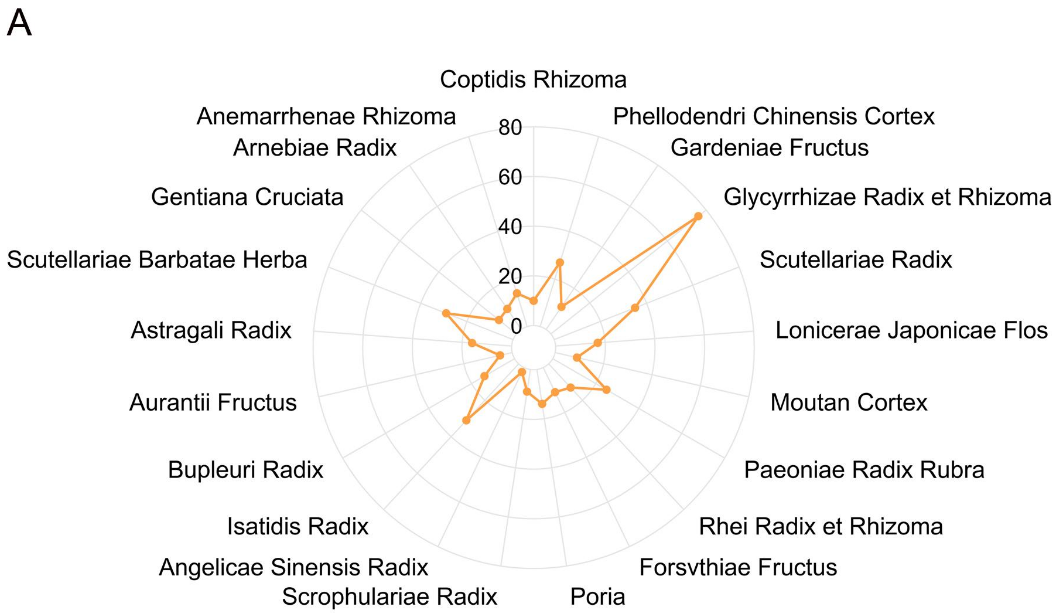

Appendix Figure S1. Network pharmacology analysis for the novel drugs possessing the abilities for the hepatic removal of excess DEHP. (A) Radar analysis of active ingredients from 21 Chinese medicines.

Appendix Figure S2

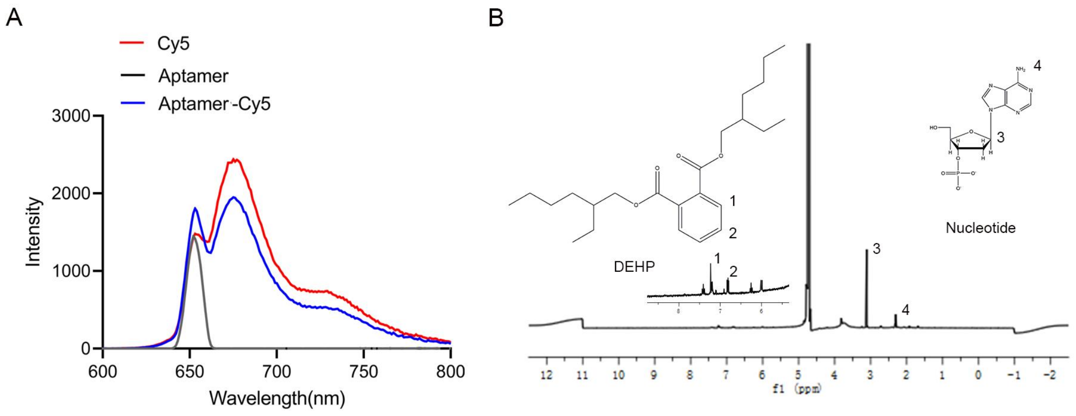

Appendix Figure S2. Validation of a long-term stability of the *csa*-DEHP system stored at -80°C for over 1 year. (**A**) Fluorescent spectra of the Cy5, aptamer and aptamer-Cy5. (**B**) ^{1}H NMR spectra of aptamer-DEHP.

Appendix Figure S3

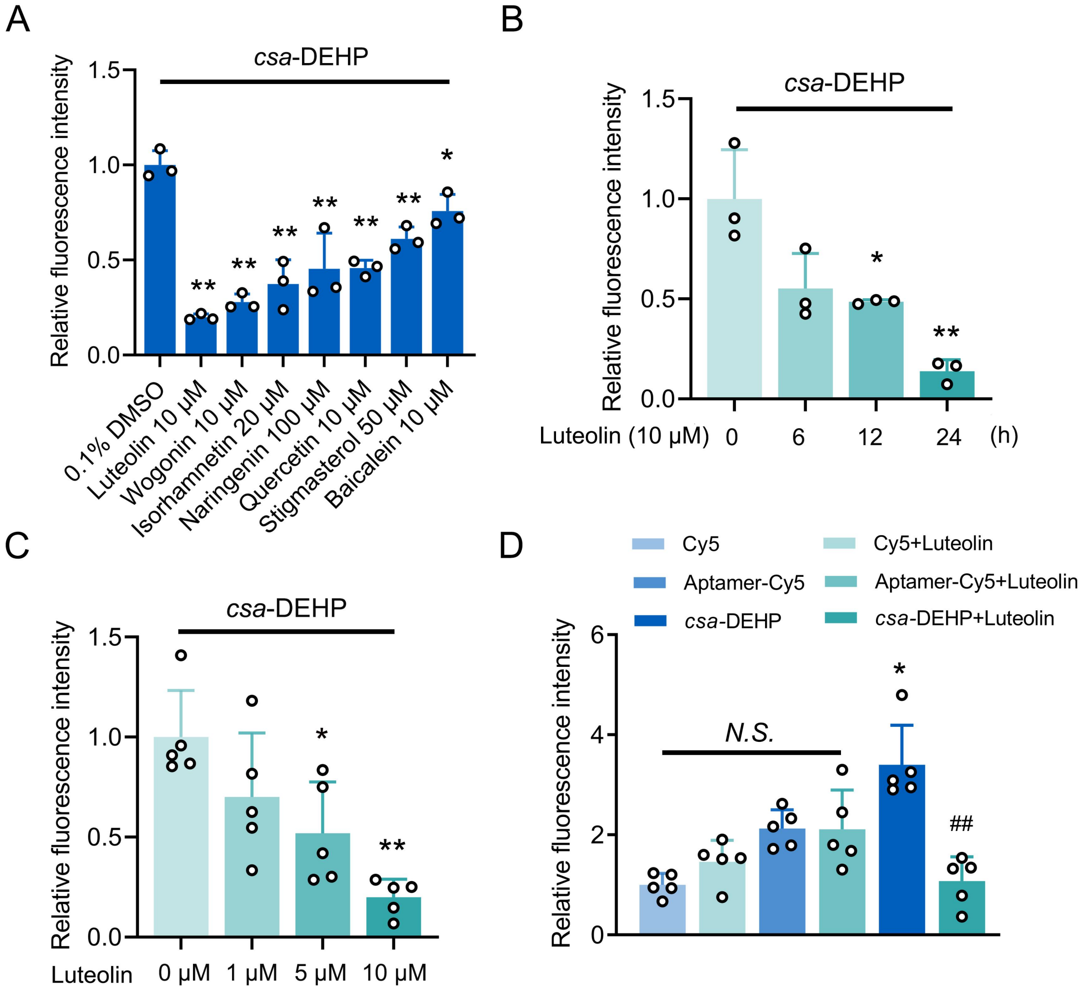

Appendix Figure S3. **Luteolin is a potential drug to decease DEHP accumulation in the mouse liver.** (**A**) Quantitative analysis of the relative fluorescence intensity in Fig. 1D. $^{*}P < 0.05$ and $^{**}P < 0.01$ *vs.* DEHP+0.1% DMSO group, n = 3. (**B**) and (**C**) Quantitative analysis of the relative fluorescence intensity in Fig. 2A. $^{*}P < 0.05$ and $^{**}P < 0.01$ *vs.* 0 h group, n = 3. $^{*}P < 0.05$ and $^{**}P < 0.01$ *vs.* 0 μM group, n = 5. **(D)** Quantitative analysis of the relative fluorescence intensity in Fig. 2B. *N.S.,* no significance, $^{*}P < 0.05$ and $^{**}P < 0.01$ *vs.* Cy5 group, $^{\#\#}P < 0.01$ *vs. csa*-DEHP group, n = 5. All the data were represented as the mean ± SD. The paired Student's *t*-test was employed to compare between two groups. One-way ANOVA with a Fisher's LSD *post hoc* test was utilized to compare among multiple groups. Exact *P* values are listed in Appendix Table S11.

Appendix Figure S4

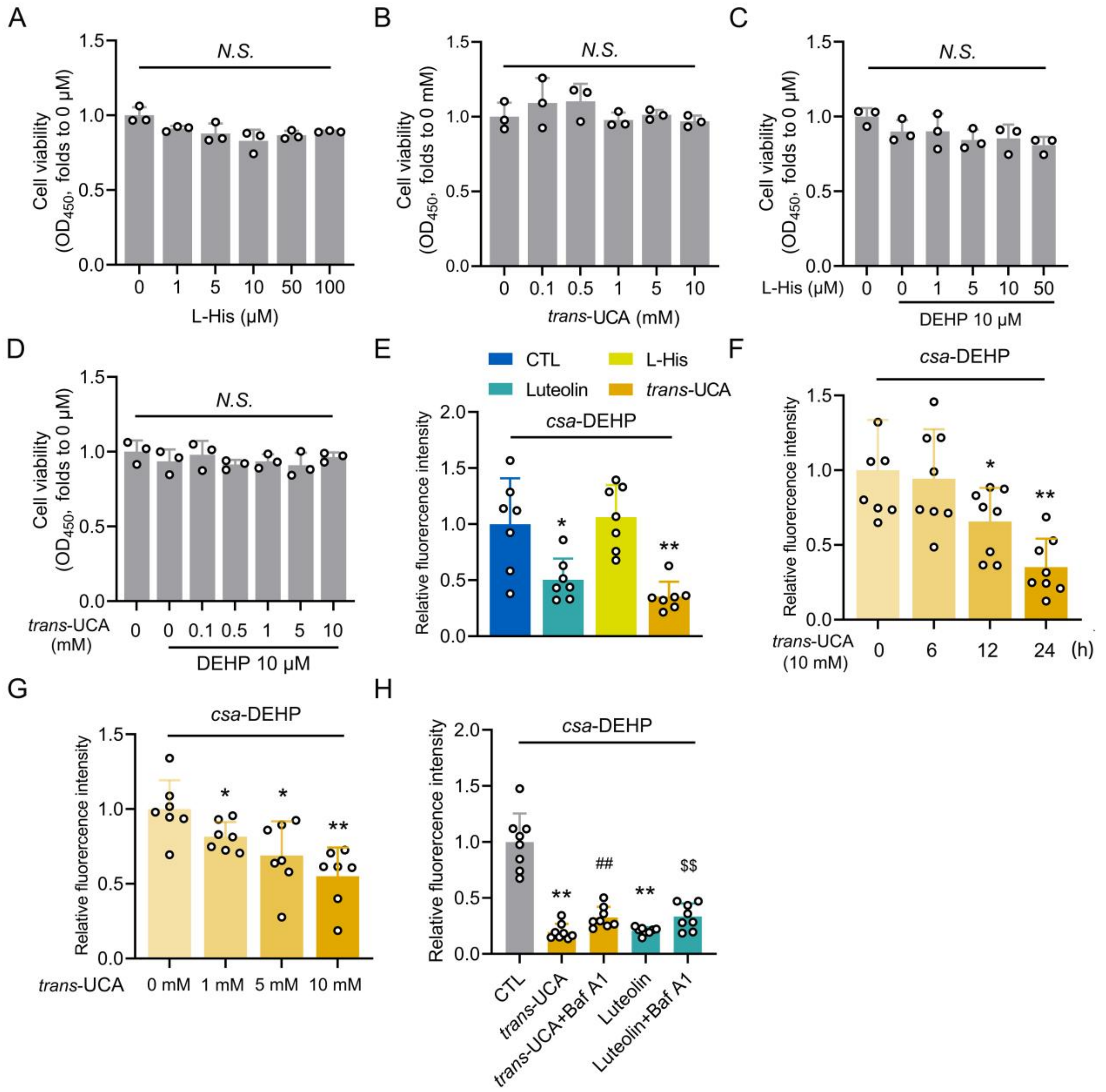

Appendix Figure S4. *Trans*-UCA serves as the functional metabolite mimicking the luteolin. (**A**-**D**) Cell viability analyses of mouse PHs treated with the different doses of L-his and *trans*-UCA. Note that DEHP were added when indicated. *N.S.*, no significance. n = 3. (**E**) Quantitative analysis of the relative fluorescence intensity in Fig.5B. $^{*}P < 0.05$ and $^{**}P < 0.01$ *vs.* CTL group. n = 7. (**F**) and (**G**) Quantitative analysis of the relative fluorescence intensity in Fig. 5D. $^{*}P < 0.05$ and $^{**}P < 0.01$ *vs.* 0 h or 0 μM group. n = 7. (**H**) Quantitative analysis of the relative fluorescence intensity in Fig. 6E. $^{**}P < 0.01$ *vs.* CTL group, $^{\#\#}P < 0.01$ *vs*. *trans*-UCA group, $^{\$\$}P < 0.01$ *vs*. Luteolin group. n = 8. All the data were represented as the mean ± SD. The paired Student's *t*-test was employed to compare between two groups. One-way ANOVA with a Fisher's LSD *post hoc* test was utilized to compare among multiple groups. Exact *P* values are listed in Appendix Table S11.

Appendix Table S1. Numbers of active ingredients from 21 Chinese medicines.

Effective Drug Ingredients	Effective Chemical Compound Counts
Glycyrrhizae Radix et Rhizoma	76
Scutellariae Radix	35
Isatidis Radix	31
Scutellariae Barbatae Herba	29
Phellodendri Chinensis Cortex	27
Paeoniae Radix Rubra	25
Lonicerae Japonicae Flos	17
Astragali Radix	16
Poria	14
Bupleuri Radix	14
Anemarrhenae Rhizoma	14
Rhei Radix et Rhizoma	13
Gardeniae Fructus	11
Forsvthiae Fructus	11
Coptidis Rhizoma	10
Arnebiae Radix	10
Moutan Cortex	9
Scrophulariae Radix	9
Gentiana Cruciata	9
Aurantii Fructus	5
Angelicae Sinensis Radix	2

Appendix Table S2. Topological calculations of effective chemical compounds.

Effective Chemical Compounds	Degree
β-Sitosterol	12
Sitosterol	11
Stigmasterol	10
Quercetin	10
Kaempferol	9
Baicalin	4
Mairin	4
Baicalein	3
Luteolin	3
Wogonin	3
Isorhamnetin	3
Coptisine	3
Mandenol	3
Ethyloleate	3

Appendix Table S3. Sequences of candidate aptamers targeting DEHP.

Aptamer	Sequence (5'-3')	ΔG (kcal/mol)
Seq.1	TCACGTAGAGAAAGGGGACGTGGGGGA GCTGTGCGGTCGG	-4.9
Seq.2	GGCCCACAGATAGTAGAACCACGGCGG GCGAATGGGGGCG	-9.3
Seq.3	CCGGCGCCAAGACCCCCTCGTGTCGAC ATACCGTGCGTGC	-6.7
Seq.4	CCCGCGAGGGCTCCGTCAGGGCTGCCA GGACGTGCTGCCG	-11.9
Seq.5	CCCCGGCAGGCGGTGGAGCCCAGGCTG ACGTAGCGAGTTG	-9.9
Seq.6	CGGCCCGCTACCTTCCGGAGGGTTTACT AGTGCGCCGCGG	-13.4
Seq.7	GCAACGACCTGAGCGCCAACACGGTTA ATGGATATGCCGC	-4.7
Seq.8	GAGCGGGGGGAGACACCCGCACCCGAA CTTGATTCGGATG	-15.8
Seq.9	CTCGACAGGGGTAATCGGGATGGAAAG GATCGTGGTGGCG	-8.7
Seq.10	TTCCGGGCATACGTATCGAATGTTGTGA GCCGTGGGCTGC	-9.9
Seq.11	GGGCGGAATGGATACGGTGGATGTGAC GCGAGGCTAGAGA	-3.8
Seq.12	GGCGCAGAGGAACTCAGGCACGCCTTC GGACCGCAGCCTG	-8.2
Seq.13	GCGTCTGCGTGCGCCGAAGGCACAGGC CTCGGGCAGAGAC	-17.2
Seq.14	CGCCCAACAGGACAGACGTCAGTGTTT CGTCAGGCGGCCC	-11.8
Seq.15	GGGGGCGGGAGATGACGTATAGTGGCG ACGCGGCGTGGGC	-5.8
Seq.16	CGGGGTGTGCGTGAGTGATCTCCCCCTG CGCCGCCGAGCC	-14.4

Seq.17	GCACGAAGGGAGGTGATTTGGCAGGAT GGTCGCGGGCGTG	-6.5
Seq.18	GGGTGATCGCCTGGGGTCCCGGGACGC CCGTAGCGGTCTG	-16.8
Seq.19	GCCAGCGTTCGGGGAATCGCCGCTGGT GTGTGGTGCGGGC	-13.1
Seq.20	GCCGGCGGACGGGCGCTCAAACAGGAG GTGAAGTGGCGAC	-7.9
Seq.21	GCCCGCAACGGACCAGACCTCACCCTC GAACGGGGGGATG	-10.1
Seq.22	GCAATGTCACGGGAGCGGATGGTGAAA TCGGCGTGGGCTC	-7.9
Seq.23	CGGGACGATCGGGTCGAGTGGCACATC GGGCGTTGTGGTG	-9.9
Seq.24	CCAGCAACACGGTCCACATCTTGTAAG GGCGGGCGGGCTG	-9.4
Seq.25	CGGGGGGGACCACAGAGGATGATCGGG CGCTGGCGGTGGG	-5.7

Appendix Table S4. MOE analysis of binding potentials of luteolin on differentially expressed proteins

Protein	S (kcal/mol)
Uroc1	-7.123
Dld	-6.5
Lqgap2	-6.4526
Eif3c	-6.2816
Actb	-6.199
Rnf213	-6.1671
Prkar1a	-6.1011
Rpn	-5.9371
Fasn	-5.8474
Picalm	-5.7623
Bccip	-5.7199
Acaa1b	-5.7154
Prss8	-5.5851
Cfap36	-5.3149
Rbx1	-5.3026
Serpina1c	-5.2459
Eif1a-1	-5.1661
Dbt	-5.0128
Fosl1	-4.6273
Ptms	-4.0883

Appendix Table S5. The sequences of ssDNA nucleotides.

ssDNA Name	Sequence (5'-3')
Random Library	ATAGGAGTCACGACGACCAG-N40-TATGTGCGTCTACCTCTTGA
Forward Primer	ATAGGAGTCACGACGACCAG
Phosphorylated Reverse Primer	P-TCAAGAGGTAGACGCACATA
Biotin-labeled Chain P1	TCAAGAGGTAGACGC

Appendix Table S6. Capture-SELEX screening condition.

ssDNA<br><br>(nmol)	Complementary Chain<br><br>(nmol)	Reverse Screening Target<br><br>(μM)	Positive Screen Target<br><br>(μM)	Incubation Time<br><br>(h)
1	1.5	-	100	12
0.1	0.15	-	60	12
0.08	0.12	60	60	2
0.08	0.12	-	60	1.5
0.08	0.12	60	60	1.5
0.08	0.12	-	40	1.5
0.06	0.09	40	40	1
0.06	0.09	40	40	0.5

Appendix Table S7. Mobile phase time table.

Time (min)	Flow (mL/min)	B%
0	0.3	50
2.5	0.4	100
3.5	0.55	100
6	0.55	100
6.5	0.3	50
8	0.3	50

Appendix Table S8. ESI ion source Settings.

Parameters	Drying Gas Temperature (℃)	Drying Gas Flow (mL/min)	Nebulizer pressure (psi)	Sheath gas temperature (℃)	Sheath gas flow (mL/min)	Capillary (V)	Ion mode
DEHP	250	1	35	400	12	4000	positive
MEHP	250	10	35	400	8	4000	negative

Appendix Table S9. Scan segments.

Compound	Precursor Ion (m/z)	Product Ion 1 (m/z)	Collision Energy 1 (v)	Product Ion 2 (m/z)	Collision Energy 2 (v)	Dwell	Fragmentor (v)	Cell Accelerator Voltage (v)	Declustering Potential (v)
DEHP	391	167	19	149	25	200	170	4	51
DEHP-D4	395	153	33	—	—	200	170	4	81
MEHP	277	127	14	134	16	200	170	4	31
MEHP-D4	281	138	14	—	—	200	150	4	21

Appendix Table S10. Lists of primer sequences for qPCR analysis.

Mouse Genes	Forward Primer Sequences (5'-3')	Reverse Primer Sequences (5'-3')
Uroc1	ACATGCTCCGGTTAGAACTCC	GTGGGAAATAGCGCAAGGC
36B4	GAAACTGCTGCCTCACATCCG	GCTGGCACAGTGACCTCACACG

Appendix Table S11. Exact *P* values.

Fig. 1E

	Mouse primary hepatocytes	
Comparison	*P* value	Symbol
CTL *vs.* DEHP+0.1% DMSO	1.30185E-15	**
DEHP+0.1% DMSO *vs.* DEHP+Luteolin 10 μM	6.96359E-15	##
DEHP+0.1% DMSO *vs.* DEHP+Wogonin 10 μM	2.56552E-14	##
DEHP+0.1% DMSO *vs.* DEHP+Isorhamnetin 20 μM	6.96974E-14	##
DEHP+0.1% DMSO *vs.* DEHP+Naringenin 100 μM	2.15551E-12	##
DEHP+0.1% DMSO *vs.* DEHP+Quercetin 10 μM	2.65743E-12	##
DEHP+0.1% DMSO *vs.* DEHP+Stigmasterol 50 μM	1.28108E-11	##
DEHP+0.1% DMSO *vs.* DEHP+Baicalein 10 μM	1.62041E-11	##

Fig. 2C

	Liver		Serum		Urine	
Comparison	*P* value	Symbol	*P* value	Symbol	*P* value	Symbol
CTL *vs.* DEHP	3.28832E-12	**	0.00075853	**	2.8588E-05	**
DEHP *vs.* DEHP+Luteolin	5.30258E-09	##	0.04278569	#	0.00406305	##

Fig. 2D

	Liver		Serum		Urine	
Comparison	*P* value	Symbol	*P* value	Symbol	*P* value	Symbol
CTL *vs.* DEHP	8.2757E-06	**	1.5154E-08	**	1.154E-05	**
DEHP *vs.* DEHP+Luteolin	1.63043E-05	##	1.5777E-05	##	0.00063033	##

Fig. 2E

	AST		ALT	
Comparison	*P* value	Symbol	*P* value	Symbol
CTL *vs.* DEHP	0.001077335	**	0.00082558	**
DEHP *vs.* DEHP+Luteolin	0.003216993	##	0.0001713	##

Fig. 3D

	Mouse primary hepatocytes	
Comparison	*P* value	Symbol
CTL *vs.* DEHP	0.256507824	*N.S.*

Fig. 3E

	Mouse primary hepatocytes	
Comparison	*P* value	Symbol
DEHP *vs.* DEHP+Luteolin	0.065744554	*N.S.*

Fig. 4A

	Mouse primary hepatocytes	
Comparison	*P* value	Symbol
DEHP CTL *vs.* DEHP+MG132	0.000519587	**
DEHP CTL *vs.* DEHP+Baf A1	0.005020217	**
DEHP+Luteolin *vs.* DEHP+Luteolin+Baf A1	0.004384965	##
DEHP+Luteolin *vs.* DEHP+Luteolin+MG132	0.281635733	*N.S.*

Fig. 4F

	Liver		Serum		Urine	
Comparison	*P* value	Symbol	*P* value	Symbol	*P* value	Symbol
AAV-TBG+DEHP *vs.* AAV-TBG+DEHP+Luteolin	6.07118E-06	**	9.549E-06	**	0.00093172	**
AAV-TBG+DEHP+Luteolin *vs.* AAV-Uroc1+DEHP+Luteolin	1.77384E-05	##	0.00133261	##	0.00014461	##

Fig. 4G

	Liver		Serum		Urine	
Comparison	*P* value	Symbol	*P* value	Symbol	*P* value	Symbol
AAV-TBG+DEHP *vs.* AAV-TBG+DEHP+Luteolin	0.000694739	**	0.00258238	**	4.9988E-07	**
AAV-TBG+DEHP+Luteolin *vs.* AAV-Uroc1+DEHP+Luteolin	0.0023174	##	0.00102694	##	7.2915E-06	##

Fig. 4H

	AST		ALT	
Comparison	*P* value	Symbol	*P* value	Symbol
AAV-TBG+DEHP *vs.* AAV-TBG+DEHP+Luteolin	0.009567148	**	0.00177885	**
AAV-TBG+DEHP+Luteolin *vs.* AAV-Uroc1+DEHP+Luteolin	0.000912929	##	0.00752499	##

Fig. 5C

	Mouse primary hepatocytes	
Comparison	*P* value	Symbol
CTL *vs.* Luteolin	5.84237E-19	**
CTL *vs.* L-His	8.9528E-13	**
CTL *vs.* *trans*-UCA	6.82376E-19	**

Fig. 5E

	Liver		Serum		Urine	
Comparison	*P* value	Symbol	*P* value	Symbol	*P* value	Symbol
DEHP *vs.* DEHP+*trans*-UCA	2.03177E-09	**	0.00100796	**	0.00393928	**

Fig. 5F

	Liver		Serum		Urine	
Comparison	*P* value	Symbol	*P* value	Symbol	*P* value	Symbol
DEHP *vs.* DEHP+*trans*-UCA	0.003127038	**	0.00093581	**	4.5613E-05	**

Fig. 5G

	AST		ALT	
Comparison	*P* value	Symbol	*P* value	Symbol
DEHP *vs.* DEHP+*trans*-UCA	0.004307907	**	0.00800023	**

Fig. 6D

	Mouse primary hepatocytes	
Comparison	*P* value	Symbol
CTL *vs.* DEHP	0.027934837	*
DEHP *vs.* DEHP+Luteolin	8.95555E-06	##
DEHP *vs.* DEHP+*trans*-UCA	9.23483E-05	##

Fig. 6F

	Mouse primary hepatocytes	
Comparison	*P* value	Symbol
CTL *vs.* *trans*-UCA	2.68817E-14	**
trans-UCA *vs.* *trans*-UCA+Baf A1	2.84794E-15	##
CTL *vs.* Luteolin	4.27248E-14	**

Luteolin *vs.* Luteolin+Baf A1	2.85921E-15	$$

Fig. EV1A

	Mouse primary hepatocytes	
Comparison	*P* value	Symbol
Luteolin 0 μM *vs.* Luteolin 1 μM	0.452707913	*N.S.*
Luteolin 0 μM *vs.* Luteolin 5 μM	0.828144564	*N.S.*
Luteolin 0 μM *vs.* Luteolin 10 μM	0.760488794	*N.S.*
Luteolin 0 μM *vs.* Luteolin 25 μM	0.548575435	*N.S.*
Luteolin 0 μM *vs.* Luteolin 50 μM	0.432703877	*N.S.*

Fig. EV1B

	Mouse primary hepatocytes	
Comparison	*P* value	Symbol
Wogonin 0 μM *vs* . Wogonin 1 μM	0.854462689	*N.S.*
Wogonin 0 μM *vs.* Wogonin 5 μM	0.148159157	*N.S.*
Wogonin 0 μM *vs.* Wogonin 10 μM	0.811876168	*N.S.*
Wogonin 0 μM *vs.* Wogonin 25 μM	0.42360741	*N.S.*
Wogonin 0 μM *vs.* Wogonin 50 μM	0.9053992	*N.S.*

Fig. EV1C

	Mouse primary hepatocytes	
Comparison	*P* value	Symbol
Isorhamnetin 0 μM *vs.* Isorhamnetin 5 μM	0.280165456	*N.S.*
Isorhamnetin 0 μM *vs.* Isorhamnetin 10 μM	0.824508541	*N.S.*
Isorhamnetin 0 μM *vs.* Isorhamnetin 20 μM	0.072491249	*N.S.*
Isorhamnetin 0 μM *vs.* Isorhamnetin 40 μM	0.288354826	*N.S.*
Isorhamnetin 0 μM *vs.* Isorhamnetin 80 μM	0.135672806	*N.S.*

Fig. EV1D

	Mouse primary hepatocytes	
Comparison	*P* value	Symbol
Naringenin 0 μM *vs.* Naringenin 10 μM	0.062234939	*N.S.*
Naringenin 0 μM *vs.* Naringenin 50 μM	0.334608584	*N.S.*
Naringenin 0 μM *vs.* Naringenin 100 μM	0.509398801	*N.S.*
Naringenin 0 μM *vs.* Naringenin 250 μM	0.102545409	*N.S.*
Naringenin 0 μM *vs.* Naringenin 500 μM	0.000578675	**

Fig. EV1E

	Mouse primary hepatocytes	
Comparison	*P* value	Symbol
Quercetin 0 μM *vs.* Quercetin 5 μM	0.18800671	*N.S.*
Quercetin 0 μM *vs.* Quercetin 10 μM	0.773148805	*N.S.*
Quercetin 0 μM *vs.* Quercetin 50 μM	0.002582195	**
Quercetin 0 μM *vs.* Quercetin 100 μM	0.001056841	**
Quercetin 0 μM *vs.* Quercetin 250 μM	6.07501E-06	**

Fig. EV1F

	Mouse primary hepatocytes	
Comparison	*P* value	Symbol
Stigmasterol 0 μM *vs.* Stigmasterol 5 μM	0.142503184	*N.S.*
Stigmasterol 0 μM *vs.* Stigmasterol 10 μM	0.297306142	*N.S.*
Stigmasterol 0 μM *vs.* Stigmasterol 50 μM	0.629968943	*N.S.*
Stigmasterol 0 μM *vs.* Stigmasterol 100 μM	0.940043883	*N.S.*
Stigmasterol 0 μM *vs.* Stigmasterol 250 μM	0.235544603	*N.S.*

Fig. EV1G

	Mouse primary hepatocytes	
Comparison	*P* value	Symbol
Baicalin 0 μM *vs.* Baicalin 1 μM	0.227889156	*N.S.*
Baicalin 0 μM *vs.* Baicalin 5 μM	0.272846187	*N.S.*
Baicalin 0 μM *vs.* Baicalin 10 μM	0.092019537	*N.S.*
Baicalin 0 μM *vs.* Baicalin 25 μM	0.202324891	*N.S.*
Baicalin 0 μM *vs.* Baicalin 50 μM	0.290557759	*N.S.*

Fig. EV1H

	Mouse primary hepatocytes	
Comparison	*P* value	Symbol
Kaempferol 0 μM *vs.* Kaempferol 10 μM	0.000106129	**
Kaempferol 0 μM *vs.* Kaempferol 50 μM	5.83058E-06	**
Kaempferol 0 μM *vs.* Kaempferol 100 μM	5.64669E-05	**
Kaempferol 0 μM *vs.* Kaempferol 250 μM	7.73681E-06	**
Kaempferol 0 μM *vs.* Kaempferol 500 μM	5.92315E-05	**

Fig. EV1I

	Mouse primary hepatocytes	
Comparison	*P* value	Symbol
Paeoniflorin 0 μM *vs.* Paeoniflorin 10 μM	0.001013648	**
Paeoniflorin 0 μM *vs.* Paeoniflorin 50 μM	0.001777704	**
Paeoniflorin 0 μM *vs.* Paeoniflorin 100 μM	0.000685772	**
Paeoniflorin 0 μM *vs.* Paeoniflorin 250 μM	0.00794299	**
Paeoniflorin 0 μM *vs.* Paeoniflorin 500 μM	0.002052233	**

Fig. EV1J

	Mouse primary hepatocytes	
Comparison	*P* value	Symbol
Berberine Hydrochloride 0 μM *vs.* Berberine Hydrochloride 5 μM	0.016425572	*
Berberine Hydrochloride 0 μM *vs.* Berberine Hydrochloride 10 μM	0.000266002	**
Berberine Hydrochloride 0 μM *vs.* Berberine Hydrochloride 20 μM	1.87044E-07	**
Berberine Hydrochloride 0 μM *vs.* Berberine Hydrochloride 40 μM	9.86392E-07	**
Berberine Hydrochloride 0 μM *vs.* Berberine Hydrochloride 80 μM	1.23536E-06	**

Fig. EV1K

	Mouse primary hepatocytes	
Comparison	*P* value	Symbol
DEHP 0 μM *vs.* DEHP 0.025 μM	0.556173597	*N.S.*
DEHP 0 μM *vs.* DEHP 0.05 μM	0.821933516	*N.S.*

DEHP 0 μM *vs.* DEHP 0.1 μM	0.386191174	*N.S.*
DEHP 0 μM *vs.* DEHP 1 μM	0.660145219	*N.S.*
DEHP 0 μM *vs.* DEHP 10 μM	0.683516899	*N.S.*

Fig. EV1L

	Mouse primary hepatocytes	
Comparison	*P* value	Symbol
DEHP 0 μM+Luteolin 0 μM *vs.* DEHP 10 μM+Luteolin 0 μM	0.3796	*N.S.*
DEHP 10 μM+Luteolin 0 μM *vs.* DEHP 10 μM+Luteolin 1 μM	0.9847	*N.S.*
DEHP 10 μM+Luteolin 0 μM *vs.* DEHP 10 μM+Luteolin 5 μM	0.9023	*N.S.*
DEHP 10 μM+Luteolin 0 μM *vs.* DEHP 10 μM+Luteolin 10 μM	0.8694	*N.S.*
DEHP 10 μM+Luteolin 0 μM *vs.* DEHP 10 μM+Luteolin 25 μM	0.7812	*N.S.*

Fig. EV3E

	Mouse primary hepatocytes	
Comparison	*P* value	Symbol
csa-DEHP 0 μM *vs.* *csa*-DEHP 50 μM	0.9385	*N.S.*
csa-DEHP 0 μM *vs.* *csa*-DEHP 150 μM	0.1064	*N.S.*
csa-DEHP 0 μM *vs.* *csa*-DEHP 300 μM	0.5822	*N.S.*

Fig. EV3G

	Mouse primary hepatocytes	
Comparison	*P* value	Symbol
csa-DEHP 0 h *vs.* *csa*-DEHP 12 h	0.896702871	*N.S.*
csa-DEHP 0 h *vs.* *csa*-DEHP 24 h	0.816538763	*N.S.*
csa-DEHP 0 h *vs.* *csa*-DEHP 48 h	0.947853068	*N.S.*

Fig. EV3J

	Mouse primary hepatocytes	
Comparison	*P* value	Symbol
csa-DEHP *vs.* *csa*-DEHP+CPZ	5.14454E-08	**
csa-DEHP *vs.* *csa*-DEHP+EIPA	8.38874E-08	**
csa-DEHP *vs.* *csa*-DEHP+Filipin	2.81942E-05	**

Fig. EV3L

	Mouse primary hepatocytes	
Comparison	*P* value	Symbol
csa-DEHP 37 ℃ *vs.* *csa*-DEHP 4 ℃	4.14538E-06	**

Fig. EV3N

	Mouse primary hepatocytes	
Comparison	*P* value	Symbol
Cy5 *vs.* Aptamer-Cy5	0.355125043	*N.S.*
Cy5 *vs.* *csa*-DEHP	0.000171953	**

Fig. EV4A

Week	0		1		2		3		4	
Comparison	*P* value	Symbol	*P* value	Symbol	*P* value	Symbol	*P* value	Symbol	*P* value	Symbol
CTL *vs.* DEHP	0.217626917	*N.S.*	0.37783705	*N.S.*	0.25990029	*N.S.*	0.26930309	*N.S.*	0.54115803	*N.S.*
DEHP *vs.* DEHP+Luteolin	0.813267093	*N.S.*	0.38604216	*10+*	0.93456799	*N.S.*	0.93808626	*N.S.*	0.57296742	*N.S.*

Fig. EV4B

	Mouse primary hepatocytes	
Comparison	*P* value	Symbol
CTL *vs.* DEHP	0.9029	*N.S.*
DEHP *vs.* DEHP+Luteolin	0.9853	*N.S.*

Fig. EV4C

	Mouse primary hepatocytes	
Comparison	*P* value	Symbol
CTL *vs.* DEHP	0.2451	*N.S.*
DEHP *vs.* DEHP+Luteolin	0.9866	*N.S.*

Fig. EV4D

	Mouse primary hepatocytes		
Comparison	*P* value	Symbol	++
CTL *vs.* DEHP	0.0002	**	
DEHP *vs.* DEHP+Luteolin	0.0025	##	

Fig. EV4E

	Mouse primary hepatocytes	
Comparison	*P* value	Symbol
CTL *vs.* DEHP	2.78556E-07	**
DEHP *vs.* DEHP+Luteolin	1.66865E-05	##

Fig. EV5B

	Mouse primary hepatocytes	
Comparison	*P* value	Symbol
CTL *vs.* DEHP	0.820337953	*N.S.*

Fig. EV5C

	Mouse primary hepatocytes	
Comparison	*P* value	Symbol
DEHP *vs.* DEHP+Luteolin	9.5566E-08	**

Fig. EV5D

	Mouse primary hepatocytes	
Comparison	*P* value	Symbol
DEHP *vs.* DEHP+Luteolin	7.37139E-05	**

Fig. EV5F

	Mouse primary hepatocytes	
Comparison	*P* value	Symbol
Vector *vs.* Vector+Luteolin	0.007647457	**
Vector+Luteolin *vs.* Uroc1+Luteolin	3.40019E-06	##
Uroc1+Luteolin *vs.* Uroc1^{A270G}+Luteolin	0.001185679	$$
Uroc1+Luteolin *vs.* Uroc1^{V272G}+Luteolin	0.003008006	$$
Uroc1+Luteolin *vs.* Uroc1$^{A270G/V272G}$+Luteolin	3.37153E-05	$$

Fig. EV5G

Comparison	Mouse primary hepatocytes *P* value	Symbol
NC shRNA *vs.* NC shRNA+Luteolin	1.83021E-07	**
NC shRNA *vs.* Uroc1 shRNA	1.25422E-07	**
NC shRNA+Luteolin *vs.* Uroc1 shRNA+Luteolin	0.890898387	*N.S.*
Uroc1 shRNA *vs.* Uroc1 shRNA+Luteolin	0.97794089	*N.S.*

Fig. EV5H

Comparison	Mouse primary hepatocytes *P* value	Symbol
AAV-TBG *vs.* AAV-TBG+DEHP	0.135280349	*N.S.*
AAV-TBG *vs.* AAV-TBG+DEHP+Luteolin	0.9867773	*N.S.*
AAV-TBG+DEHP+Luteolin *vs.* AAV-Uroc1+DEHP+Luteolin	0.001923661	**

Fig. S3A

Comparison	Mouse primary hepatocytes *P* value	Symbol
0.1% DMSO *vs.* Luteolin 10 μM	5.73531E-05	**
0.1% DMSO *vs.* Wogonin 10 μM	0.000134842	**
0.1% DMSO *vs.* Isorhamnetin 20 μM	0.001864028	**
0.1% DMSO *vs.* Naringenin 100 μM	0.009487474	**
0.1% DMSO *vs.* Quercetin 10 μM	0.00180365	**
0.1% DMSO *vs.* Stigmasterol 50 μM	0.002384713	**
0.1% DMSO *vs.* Baicalein 10 μM	0.022284404	*

Fig. S3B

Comparison	Mouse primary hepatocytes *P* value	Symbol
Luteolin 10 μM 0 h *vs.* Luteolin 10 μM 6 h	0.06194101	*N.S.*
Luteolin 10 μM 0 h *vs.* Luteolin 10 μM 12 h	0.022321809	*
Luteolin 10 μM 0 h *vs.* Luteolin 10 μM 24 h	0.004099527	**

Fig. S3C

Comparison	Mouse primary hepatocytes *P* value	Symbol
Luteolin 0 μM *vs.* Luteolin 1 μM	0.129403378	*N.S.*
Luteolin 0 μM *vs.* Luteolin 5 μM	0.014635467	*
Luteolin 0 μM *vs.* Luteolin 10 μM	9.49763E-05	**

Fig. S3D

Comparison	Mouse primary hepatocytes *P* value	Symbol
Cy5 *vs.* Cy5+Luteolin	0.4079	*N.S.*
Cy5 *vs.* Aptamer-Cy5	0.06	*N.S.*
Aptamer-Cy5 *vs.* Aptame-Cy5+Luteolin	0.9686	*N.S.*
Aptamer-Cy5 *vs.* *csa*-DEHP	0.0205	*
csa-DEHP *vs.* *csa*-DEHP+Luteolin	0.0091	##

Fig. S4A

Comparison	Mouse primary hepatocytes *P* value	Symbol
L-His 0 μM *vs.* L-His 1 μM	0.2439	*N.S.*
L-His 0 μM *vs.* L-His 5 μM	0.3305	*N.S.*
L-His 0 μM *vs.* L-His 10 μM	0.1932	*N.S.*
L-His 0 μM *vs.* L-His 50 μM	0.2741	*N.S.*
L-His 0 μM *vs.* L-His 100 μM	0.268	*N.S.*

Fig. S4B

Comparison	Mouse primary hepatocytes *P* value	Symbol
trans-UCA 0 mM *vs.* *trans*-UCA 0.1 mM	0.2439	*N.S.*
trans-UCA 0 mM *vs.* *trans*-UCA 0.5 mM	0.3305	*N.S.*
trans-UCA 0 mM *vs.* *trans*-UCA 1 mM	0.1932	*N.S.*
trans-UCA 0 mM *vs.* *trans*-UCA 5 mM	0.2741	*N.S.*
trans-UCA 0 mM *vs.* *trans*-UCA 10 mM	0.268	*N.S.*

Fig. S4C

Comparison	Mouse primary hepatocytes *P* value	Symbol
DEHP 0 μM+L-His 0 μM *vs.* DEHP 10 μM+L-His 0 μM	0.7764	*N.S.*
DEHP 10 μM+L-His 0 μM *vs.* DEHP 10 μM+L-His 1 μM	0.9847	*N.S.*
DEHP 10 μM+L-His 0 μM *vs.* DEHP 10 μM+L-His 5 μM	0.9023	*N.S.*
DEHP 10 μM+L-His 0 μM *vs.* DEHP 10 μM+L-His 10 μM	0.8694	*N.S.*
DEHP 10 μM+L-His 0 μM *vs.* DEHP 10 μM+L-His 50 μM	0.7812	*N.S.*

Fig. S4D

Comparison	Mouse primary hepatocytes *P* value	Symbol
DEHP 0 μM+*trans*-UCA 0 mM *vs.* DEHP 10 μM+*trans*-UCA 0 mM	0.9571	*N.S.*
DEHP 10 μM+trans-UCA 0 mM *vs.* DEHP 10 μM+*trans*-UCA 0.1 mM	0.6293	*N.S.*
DEHP 10 μM+trans-UCA 0 mM *vs.* DEHP 10 μM+*trans*-UCA 0.5 mM	0.999	*N.S.*
DEHP 10 μM+trans-UCA 0 mM *vs.* DEHP 10 μM+*trans*-UCA 1 mM	0.9999	*N.S.*
DEHP 10 μM+trans-UCA 0 mM *vs.* DEHP 10 μM+*trans*-UCA 5 mM	0.9909	*N.S.*
DEHP 10 μM+trans-UCA 0 mM *vs.* DEHP 10 μM+*trans*-UCA 10 mM	0.995	*N.S.*

Fig. S4E

Comparison	Mouse primary hepatocytes *P* value	Symbol
CTL *vs.* Luteolin	0.012985709	*
CTL *vs.* L-His	0.748112087	*N.S.*
CTL *vs.* *trans*-UCA	0.001834962	**

Fig. S4F

Comparison	Mouse primary hepatocytes *P* value	Symbol
trans-UCA 0 h *vs.* *trans*-UCA 6 h	0.736693783	*N.S.*
trans-UCA 0 h *vs.* *trans*-UCA 12 h	0.03093585	*

trans-UCA 0 h *vs.* *trans*-UCA 24 h	0.000315082	**

Fig. S4G

	Mouse primary hepatocytes	
Comparison	*P* value	Symbol
trans-UCA 0 mM *vs.* *trans*-UCA 1 mM	0.043891291	*
trans-UCA 0 mM *vs.* *trans*-UCA 5 mM	0.017839878	*
trans-UCA 0 mM *vs.* *trans*-UCA 10 mM	0.000923613	**

Fig. S4H

	Mouse primary hepatocytes	
Comparison	*P* value	Symbol
CTL *vs.* *trans*-UCA	6.08022E-07	**
trans-UCA *vs.* *trans*-UCA+Baf A1	0.008663687	##
CTL *vs.* Luteolin	4.87853E-07	**
Luteolin *vs.* Luteolin+Baf A1	0.009885645	$$
