# Supplementary figures and images for: Luteolin detoxifies DEHP and prevents liver injury by degrading Uroc1 protein in mice
Source: EMBO Mol Med. 2024 Oct 29;16(11):2699–724. doi: 10.1038/s44321-024-00160-9 (PMC11555401; doi:10.1038/s44321-024-00160-9)

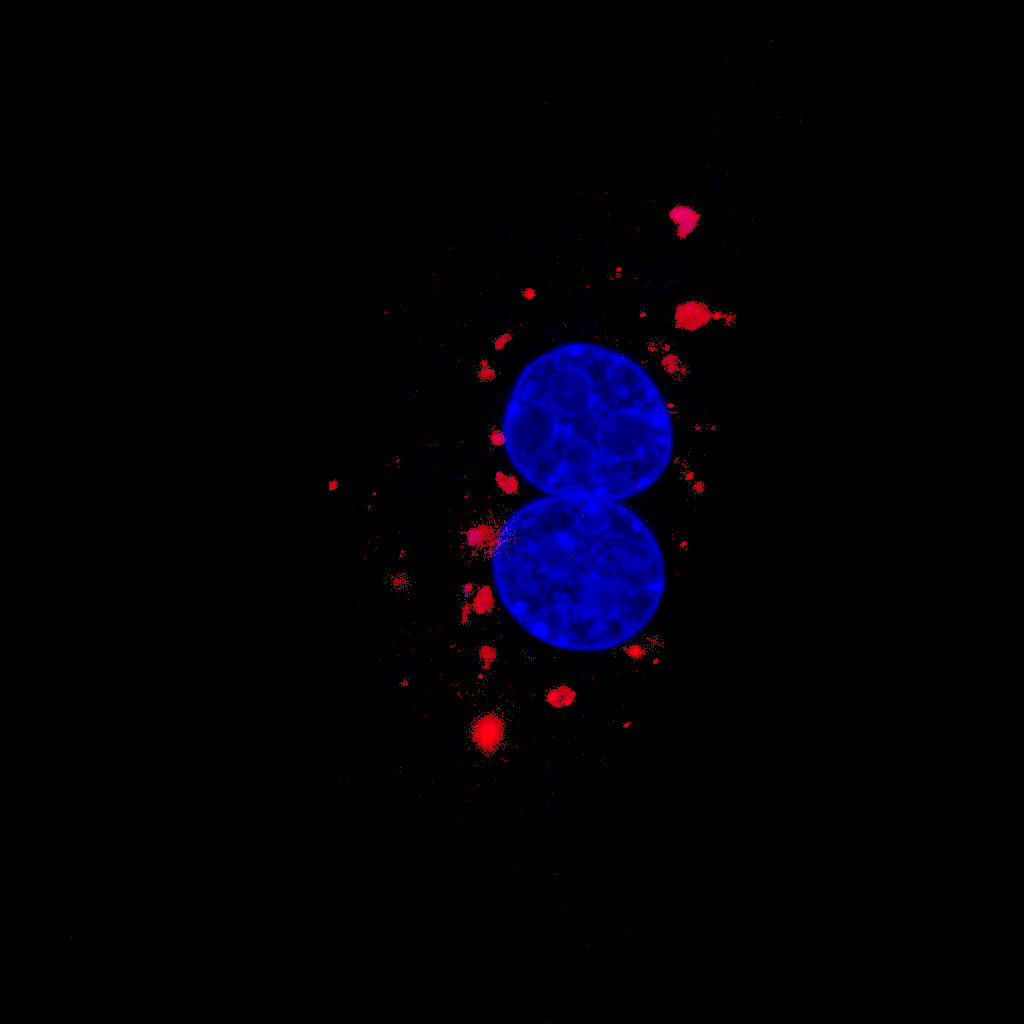

Supplement: Supplementary file 7 — Source data Fig. 1 [file 44321_2024_160_MOESM7_ESM.zip › Figure 1/1D/0.1% DMSO.tif]

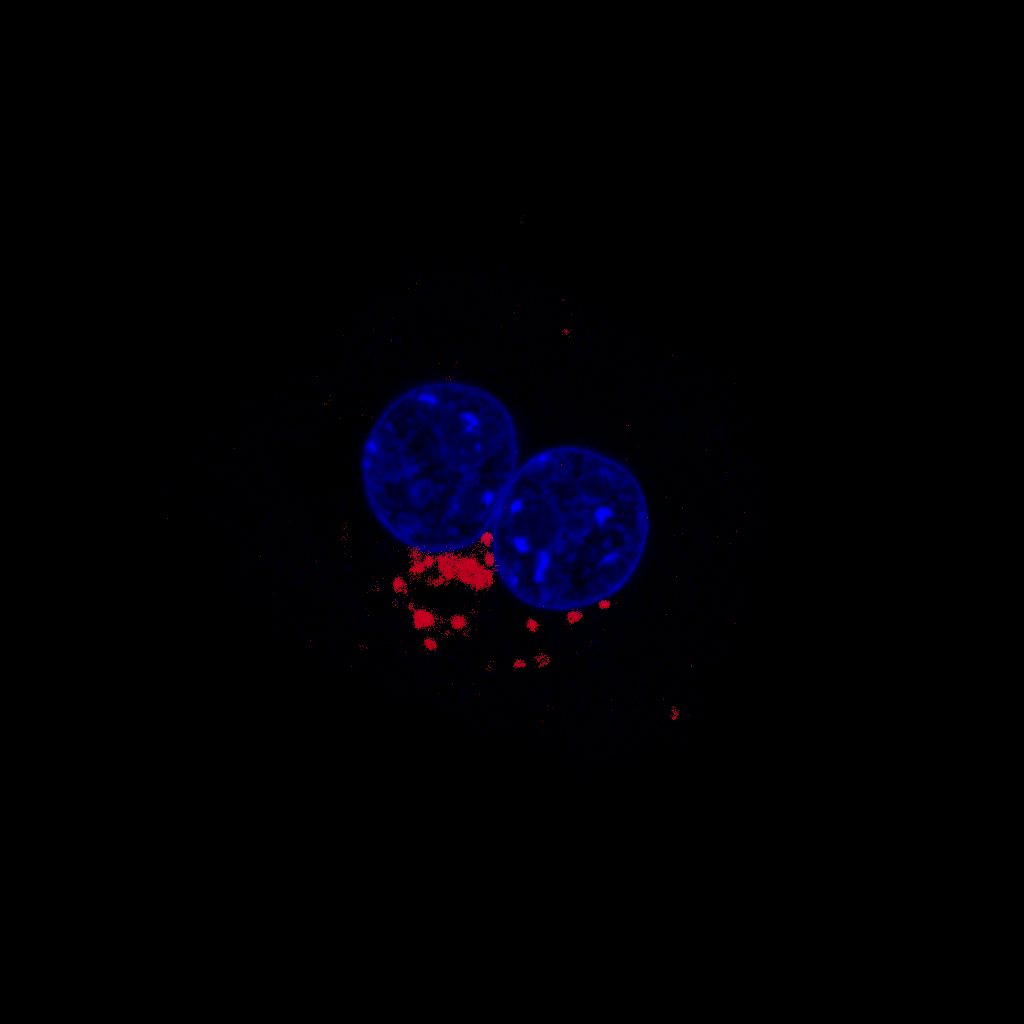

Supplement: Supplementary file 7 — Source data Fig. 1 [file 44321_2024_160_MOESM7_ESM.zip › Figure 1/1D/Baicalin.tif]

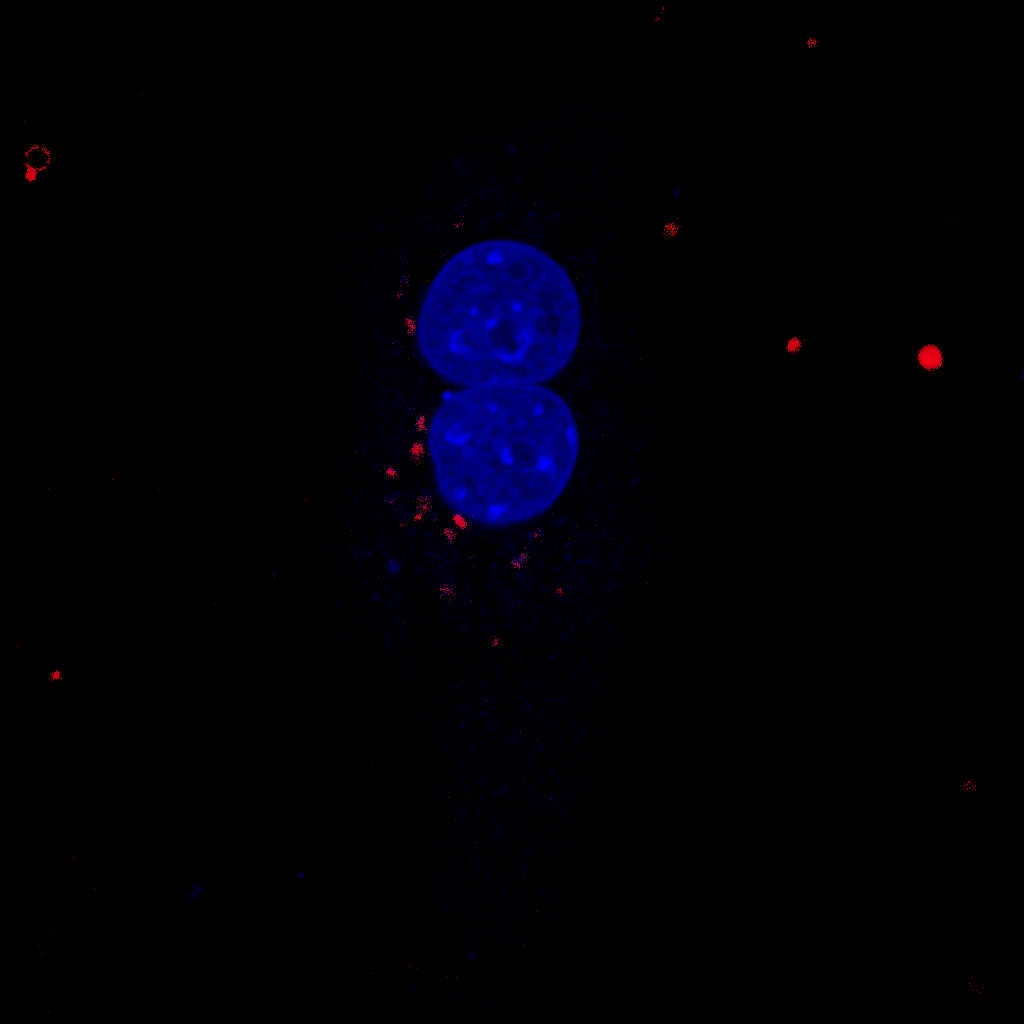

Supplement: Supplementary file 7 — Source data Fig. 1 [file 44321_2024_160_MOESM7_ESM.zip › Figure 1/1D/Isorhamnetin.tif]

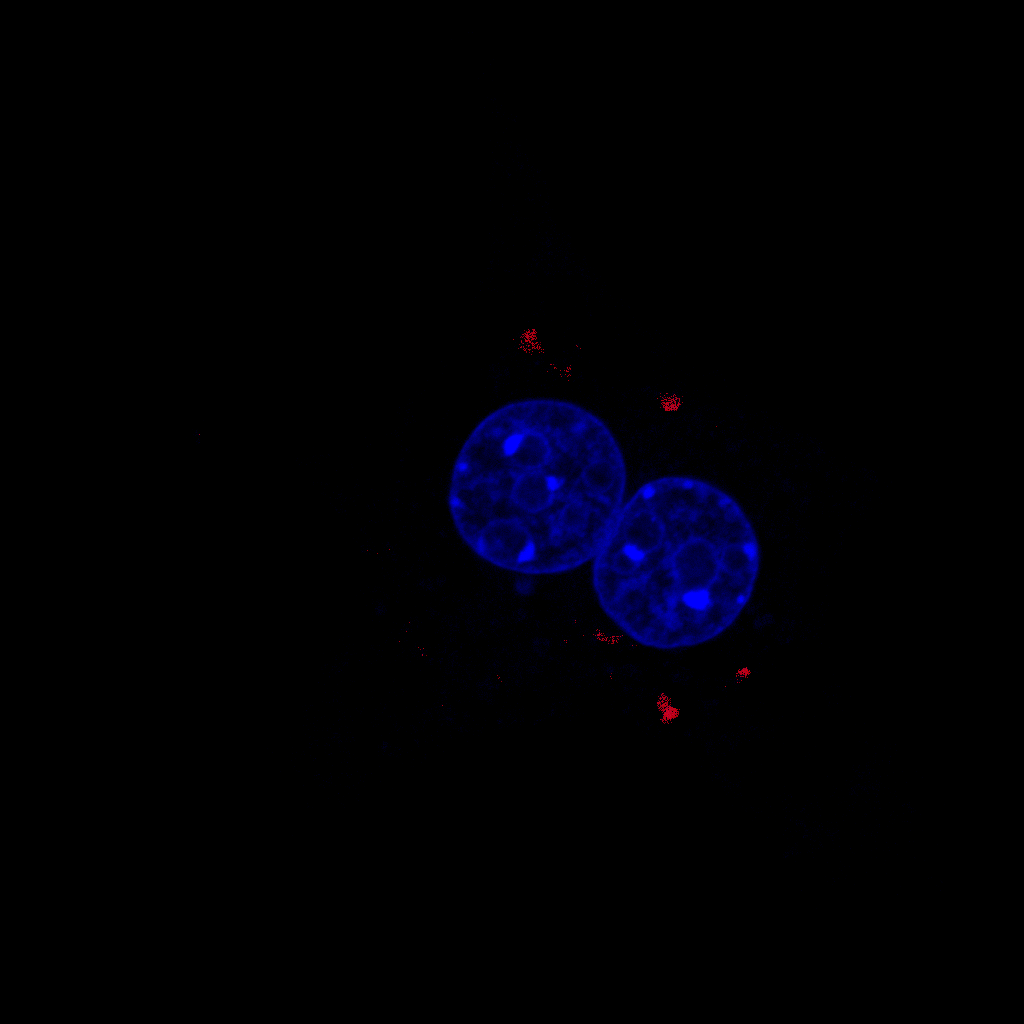

Supplement: Supplementary file 7 — Source data Fig. 1 [file 44321_2024_160_MOESM7_ESM.zip › Figure 1/1D/Luteolin.tif]

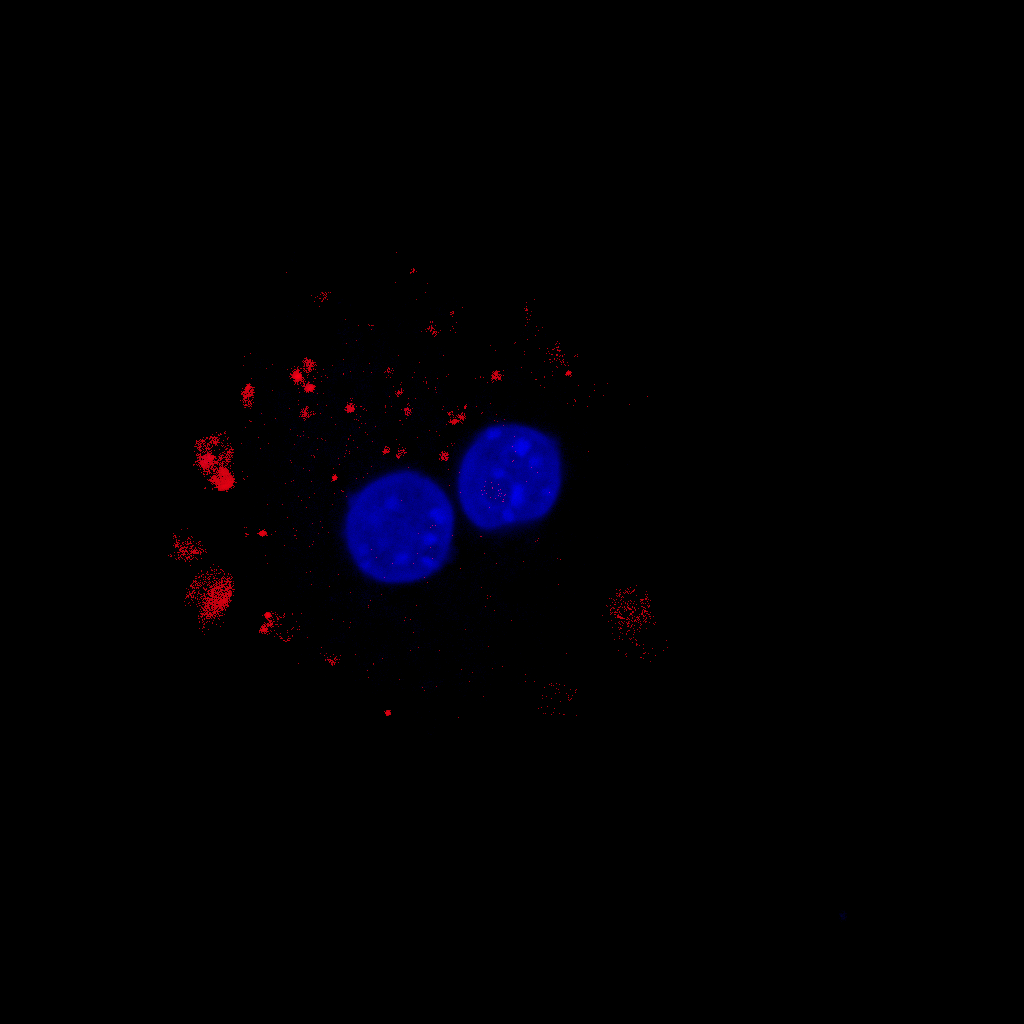

Supplement: Supplementary file 7 — Source data Fig. 1 [file 44321_2024_160_MOESM7_ESM.zip › Figure 1/1D/Naringenin.tif]

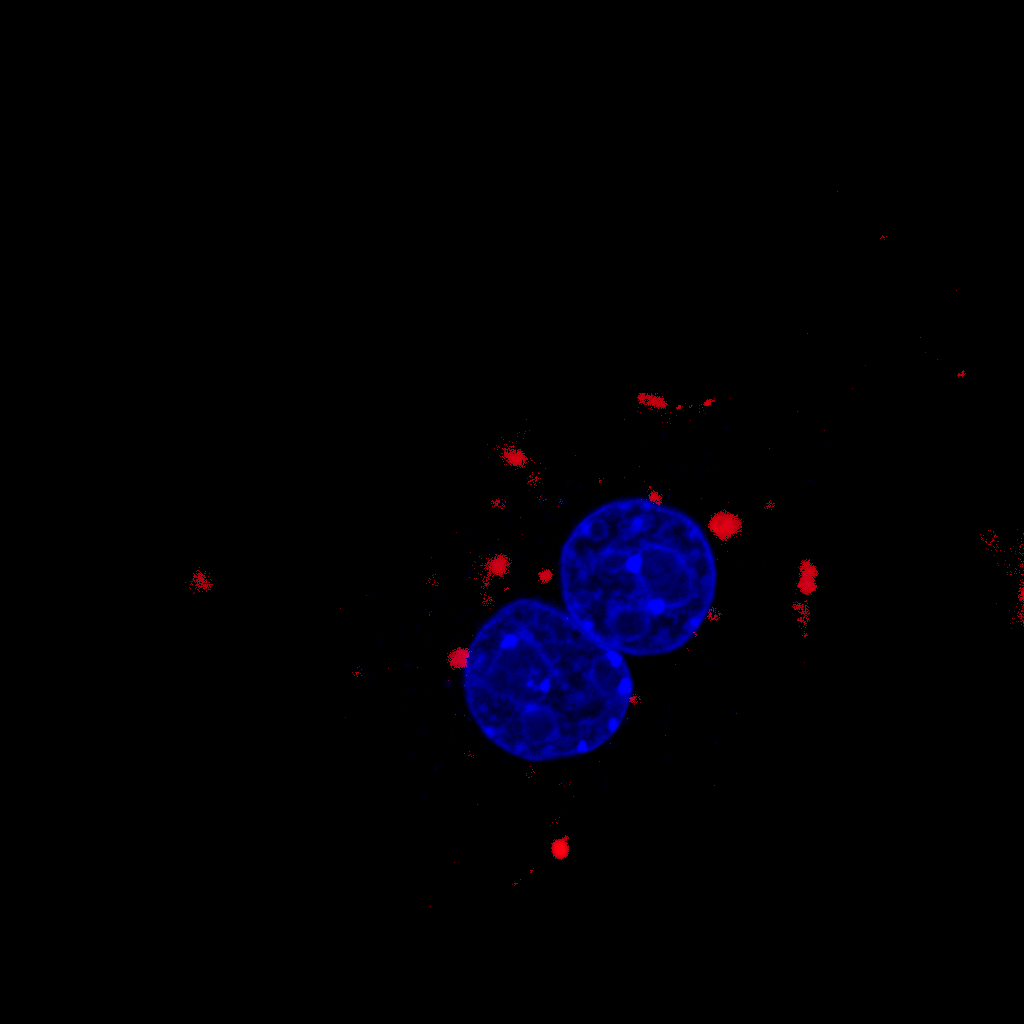

Supplement: Supplementary file 7 — Source data Fig. 1 [file 44321_2024_160_MOESM7_ESM.zip › Figure 1/1D/Quercetin.tif]

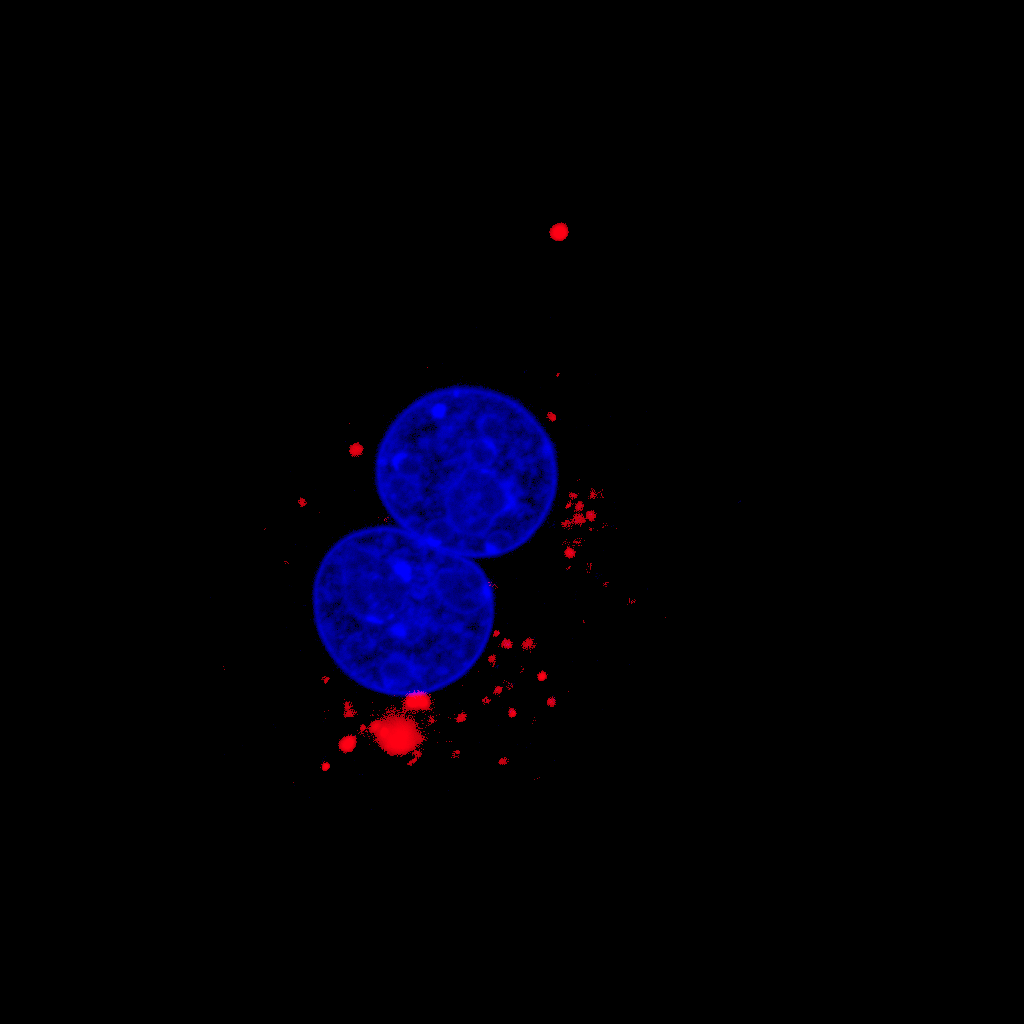

Supplement: Supplementary file 7 — Source data Fig. 1 [file 44321_2024_160_MOESM7_ESM.zip › Figure 1/1D/Stigmasterol.tif]

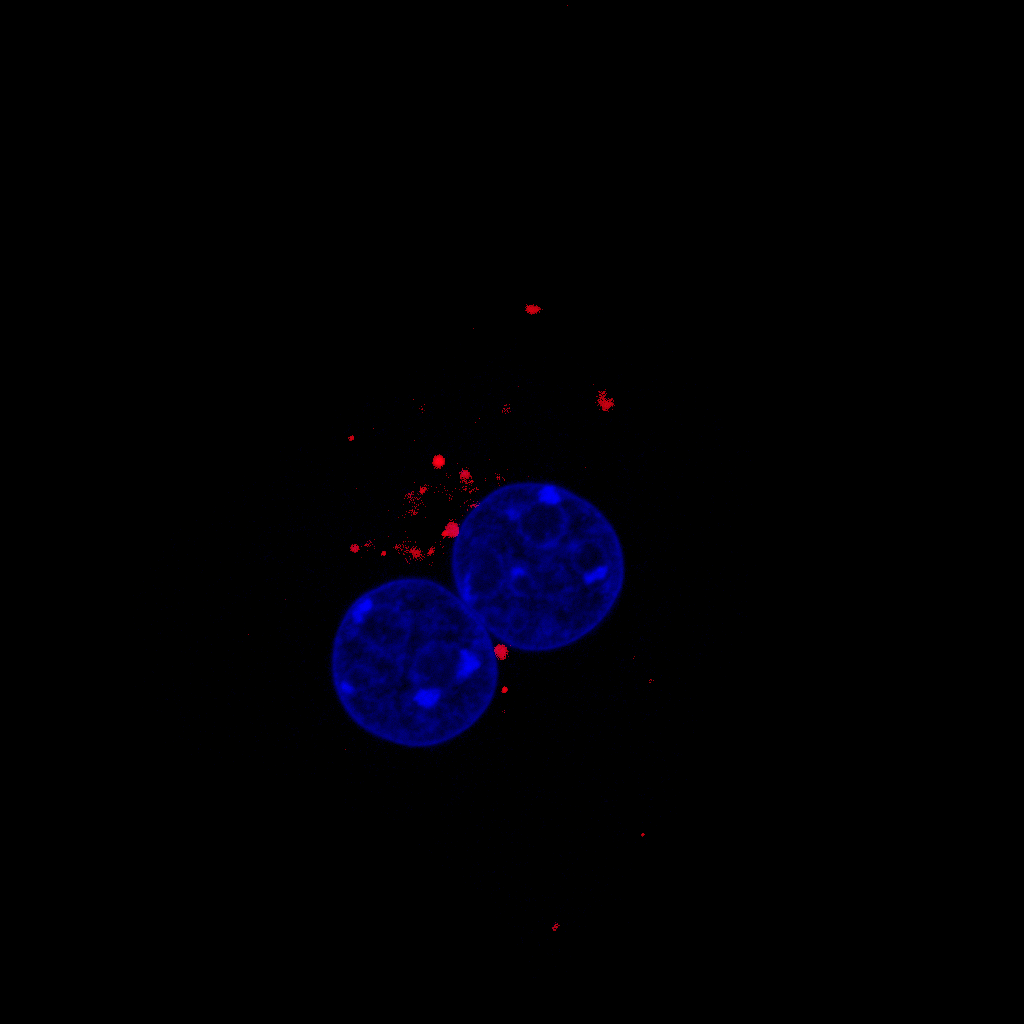

Supplement: Supplementary file 7 — Source data Fig. 1 [file 44321_2024_160_MOESM7_ESM.zip › Figure 1/1D/Wogonin.tif]

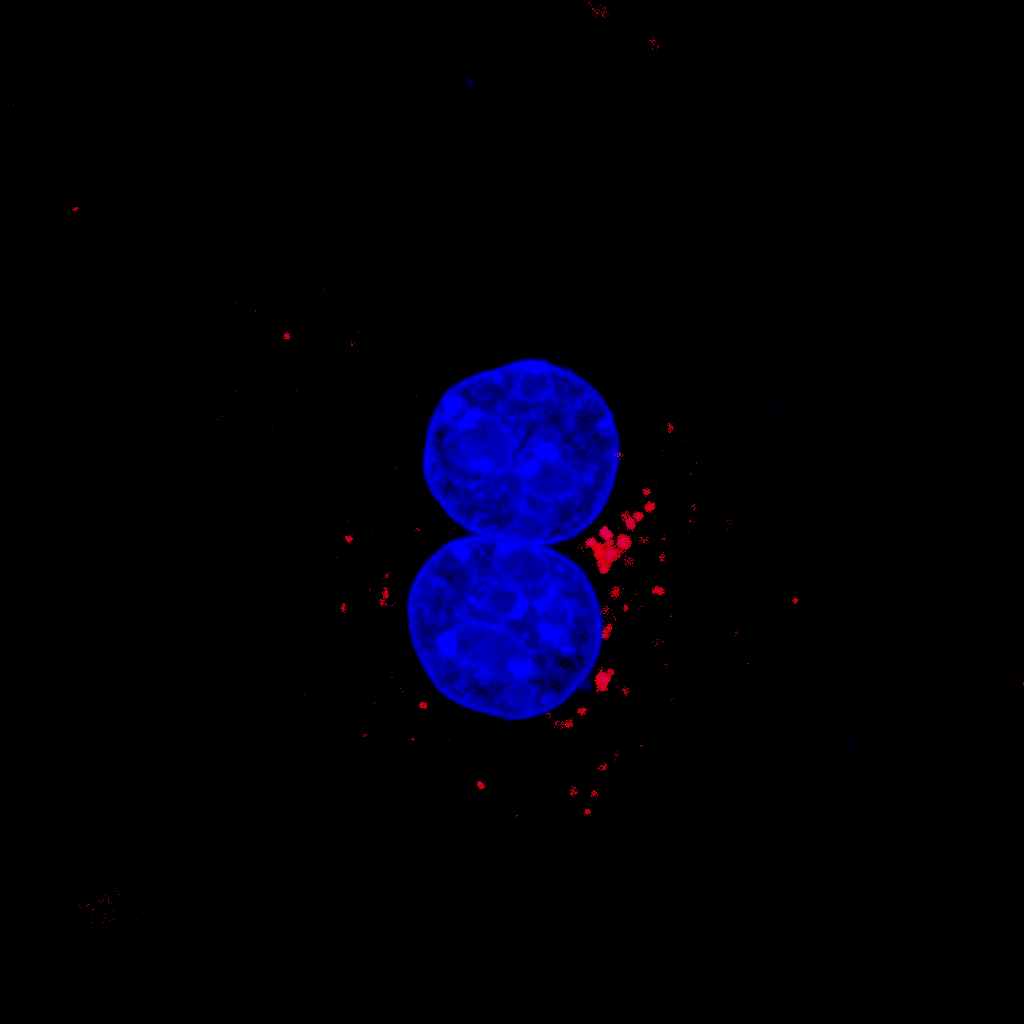

Supplement: Supplementary file 8 — Source data Fig. 2 [file 44321_2024_160_MOESM8_ESM.zip › Figure 2/2A/csa-DEHP+Luteoli 6h.tif]

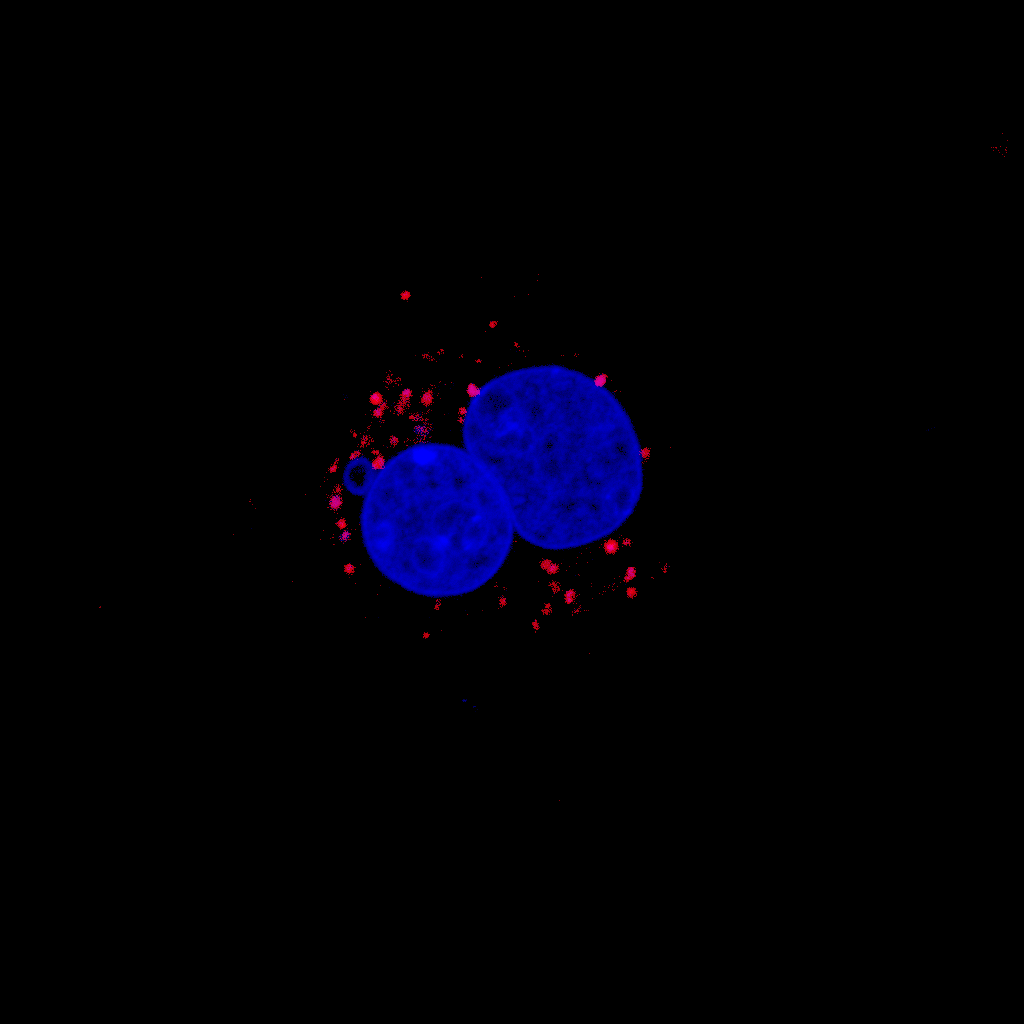

Supplement: Supplementary file 8 — Source data Fig. 2 [file 44321_2024_160_MOESM8_ESM.zip › Figure 2/2A/csa-DEHP+Luteolin 0 μM.tif]

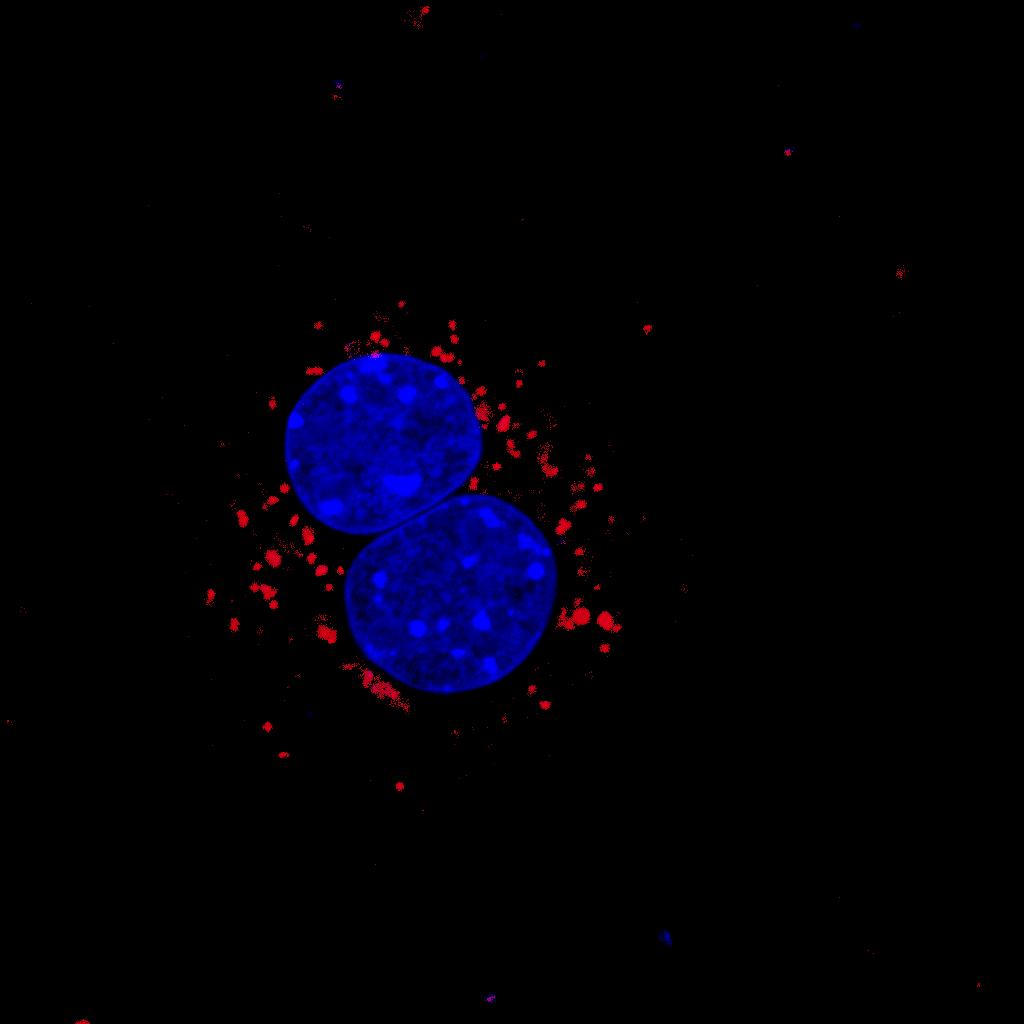

Supplement: Supplementary file 8 — Source data Fig. 2 [file 44321_2024_160_MOESM8_ESM.zip › Figure 2/2A/csa-DEHP+Luteolin 0h.tif]

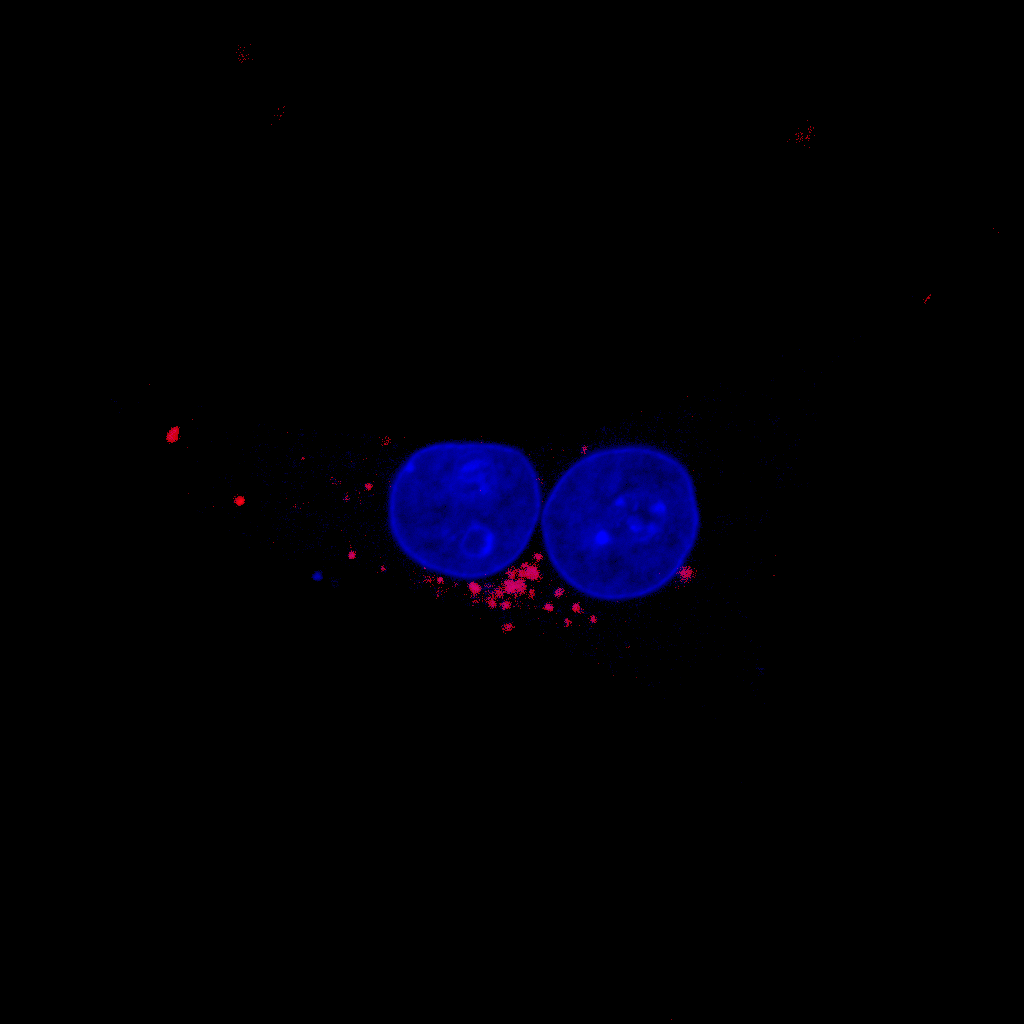

Supplement: Supplementary file 8 — Source data Fig. 2 [file 44321_2024_160_MOESM8_ESM.zip › Figure 2/2A/csa-DEHP+Luteolin 1 μM.tif]

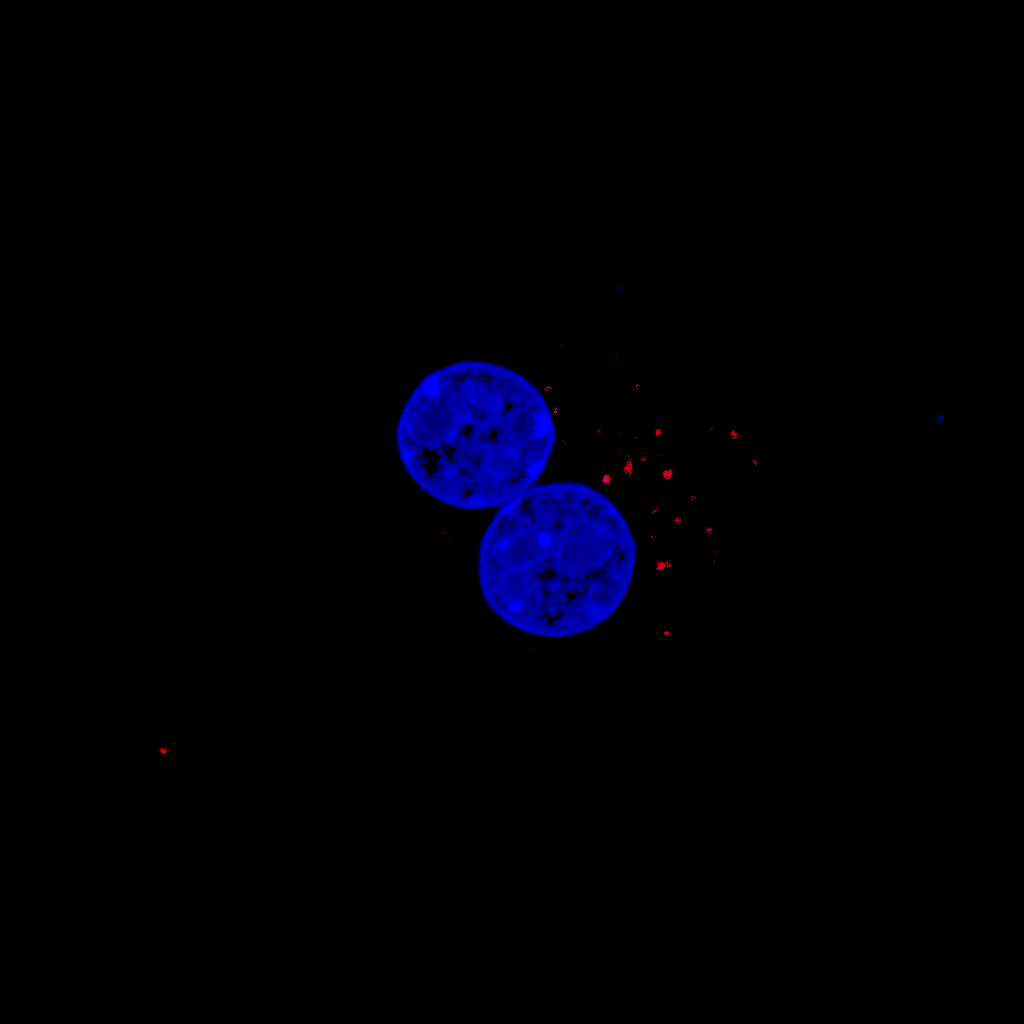

Supplement: Supplementary file 8 — Source data Fig. 2 [file 44321_2024_160_MOESM8_ESM.zip › Figure 2/2A/csa-DEHP+Luteolin 10 μM.tif]

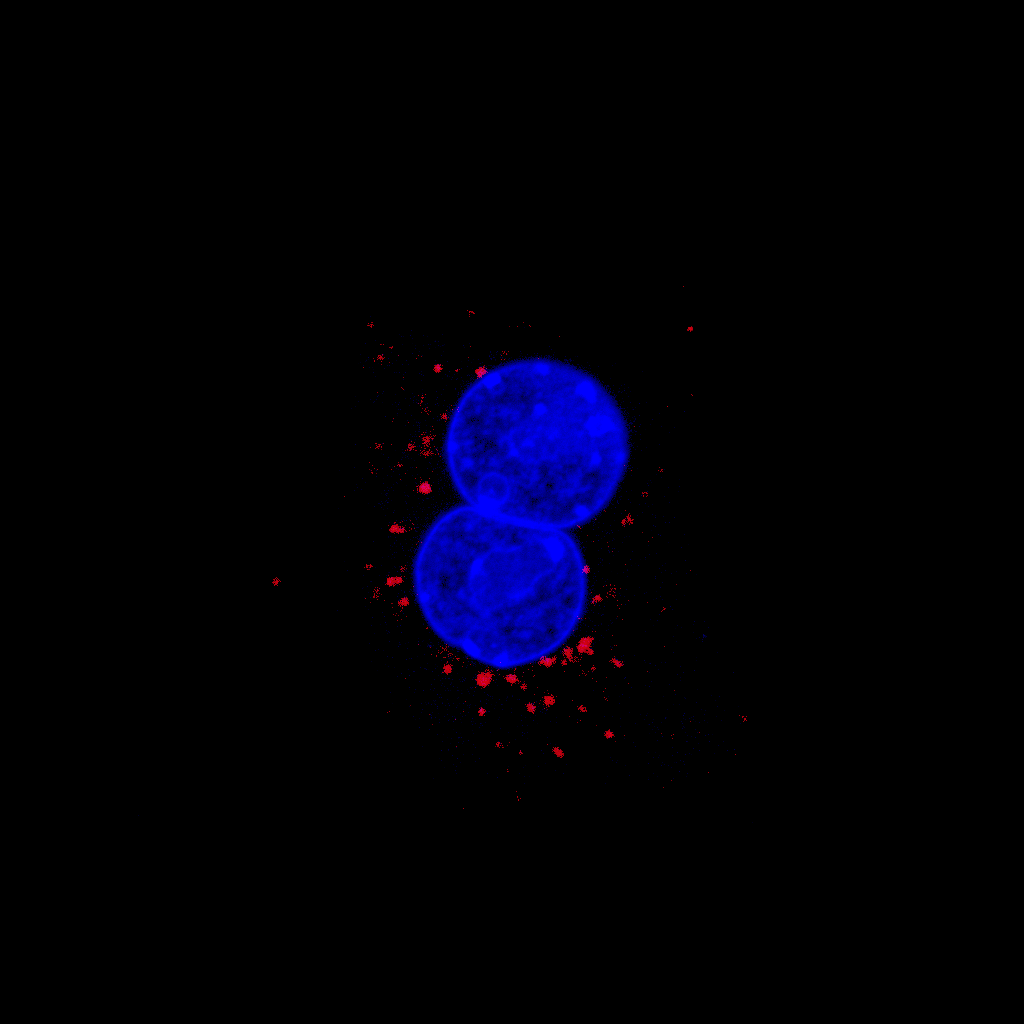

Supplement: Supplementary file 8 — Source data Fig. 2 [file 44321_2024_160_MOESM8_ESM.zip › Figure 2/2A/csa-DEHP+Luteolin 12h.tif]

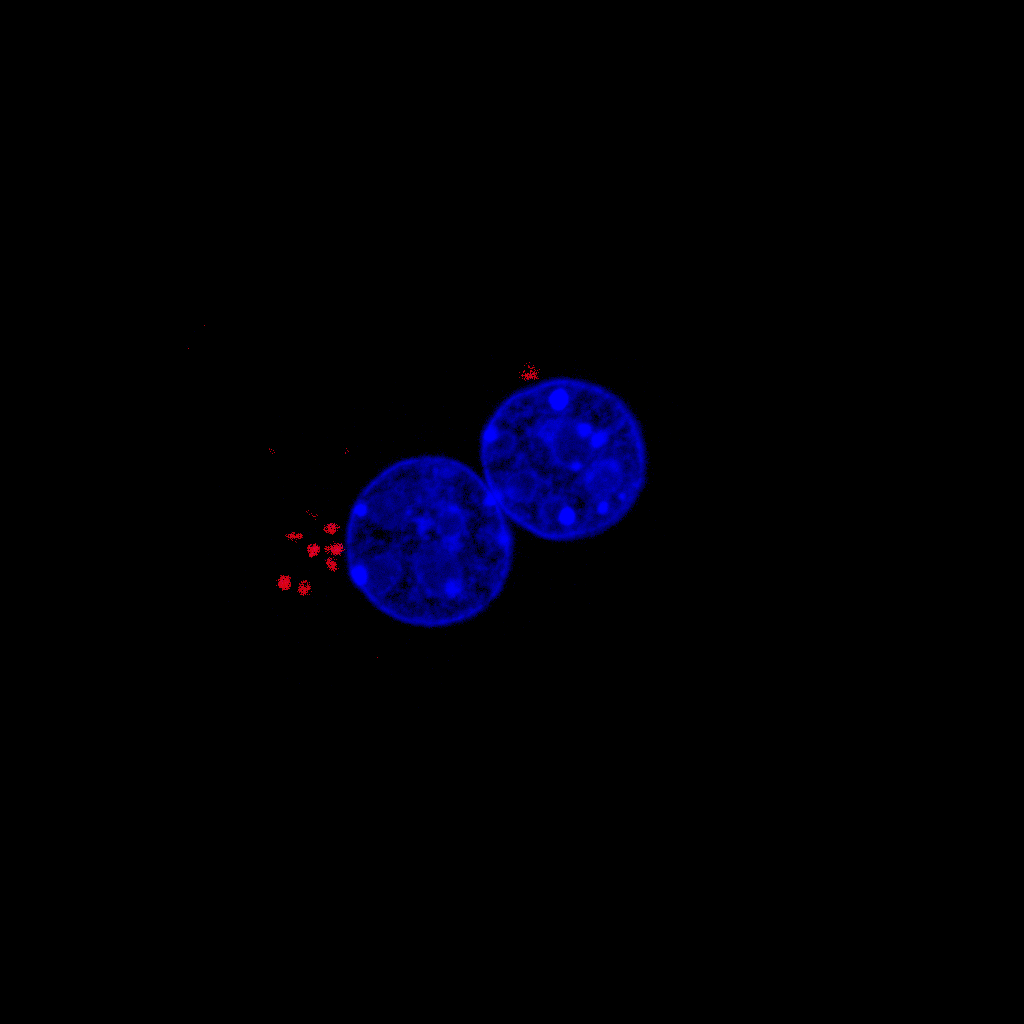

Supplement: Supplementary file 8 — Source data Fig. 2 [file 44321_2024_160_MOESM8_ESM.zip › Figure 2/2A/csa-DEHP+Luteolin 24h.tif]

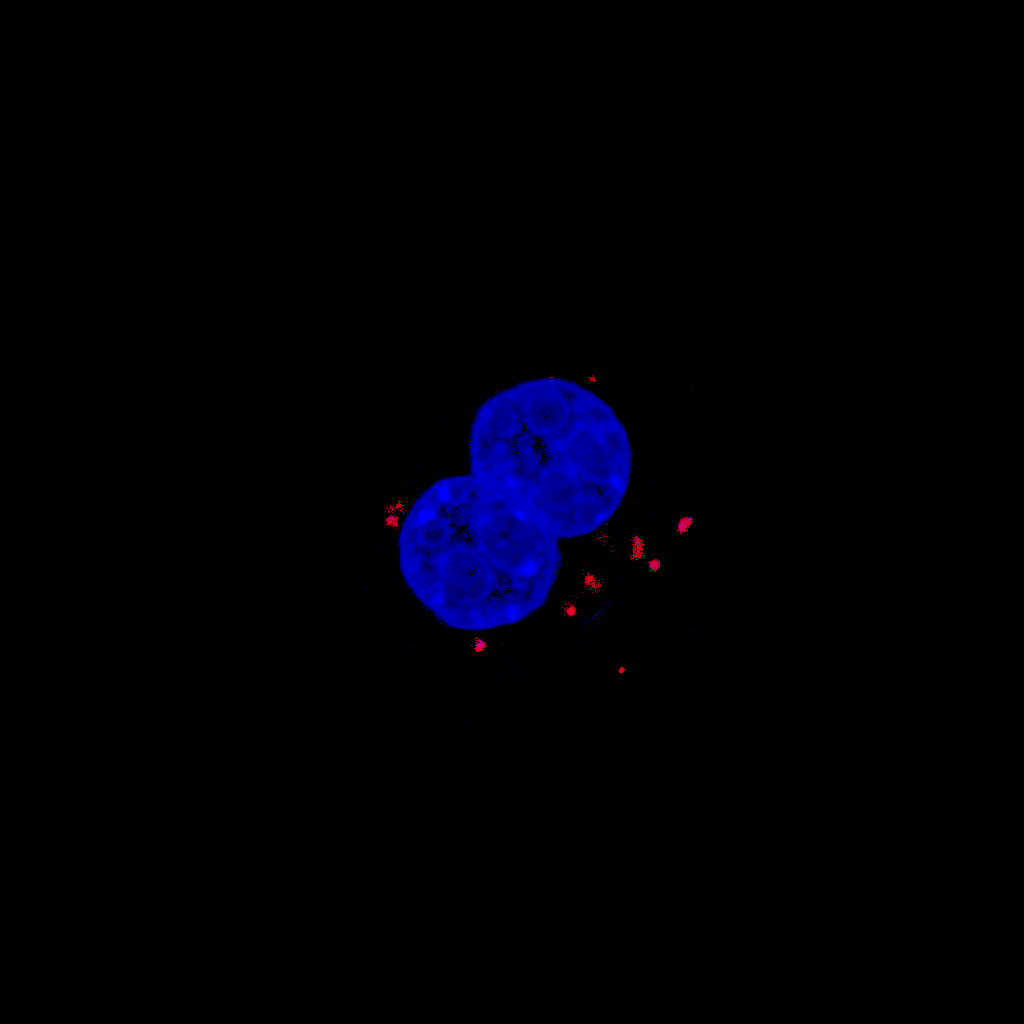

Supplement: Supplementary file 8 — Source data Fig. 2 [file 44321_2024_160_MOESM8_ESM.zip › Figure 2/2A/csa-DEHP+Luteolin 5 μM.tif]

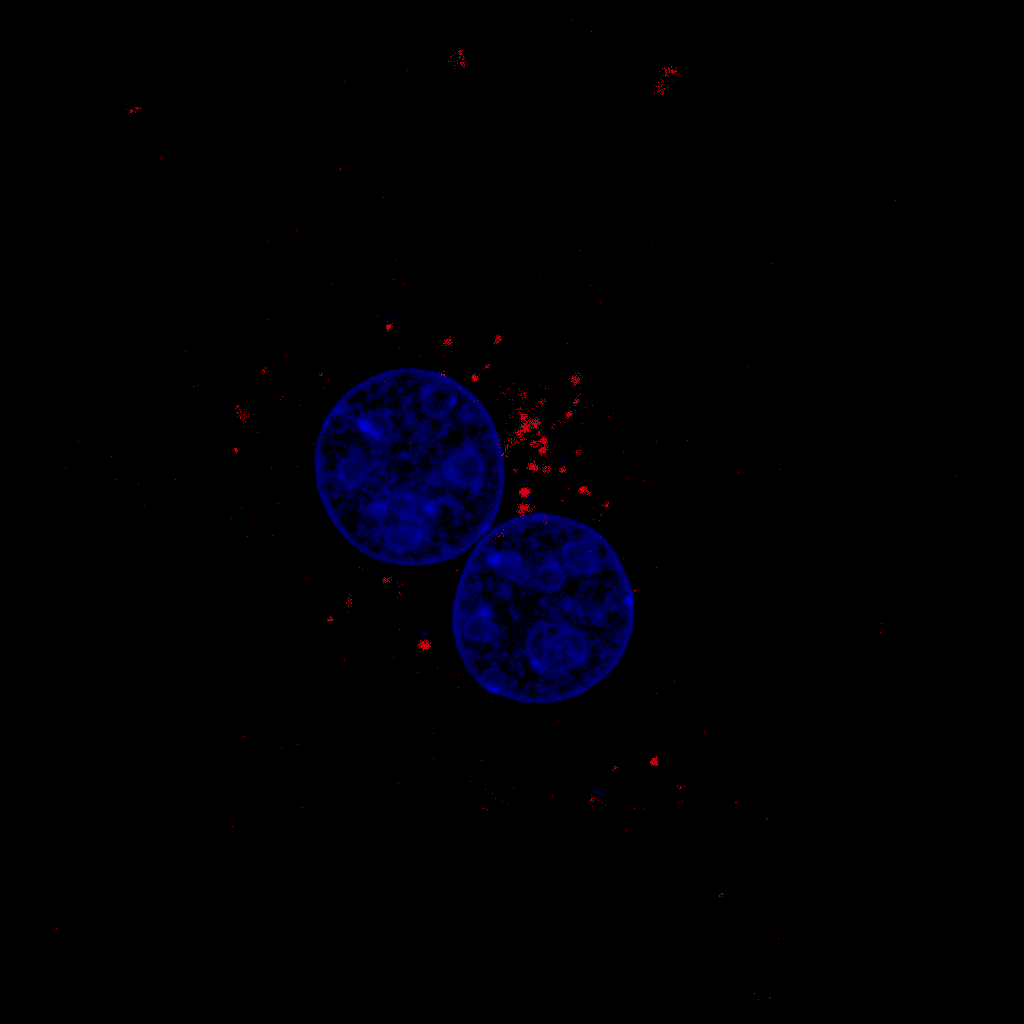

Supplement: Supplementary file 8 — Source data Fig. 2 [file 44321_2024_160_MOESM8_ESM.zip › Figure 2/2B/Aptamer-cy5 +Luteolin.tif]

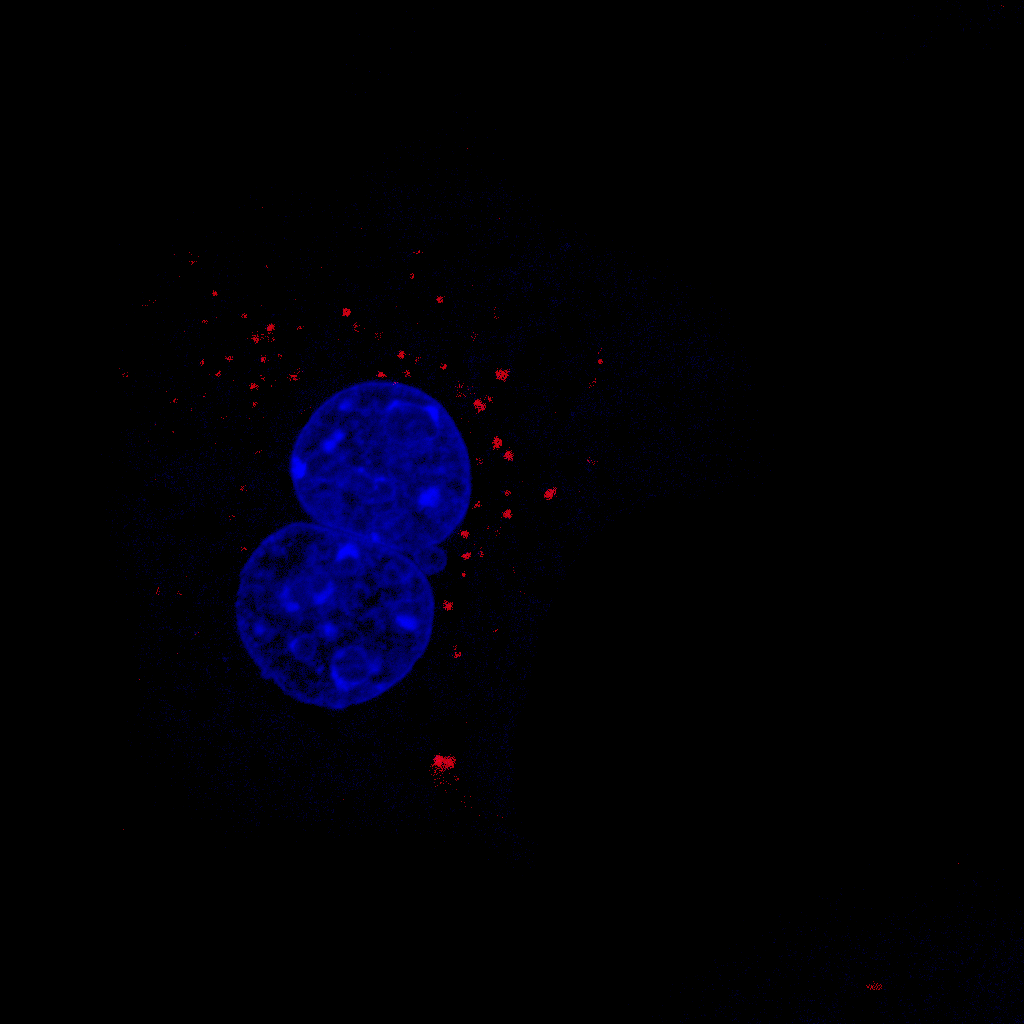

Supplement: Supplementary file 8 — Source data Fig. 2 [file 44321_2024_160_MOESM8_ESM.zip › Figure 2/2B/Aptamer-cy5.tif]

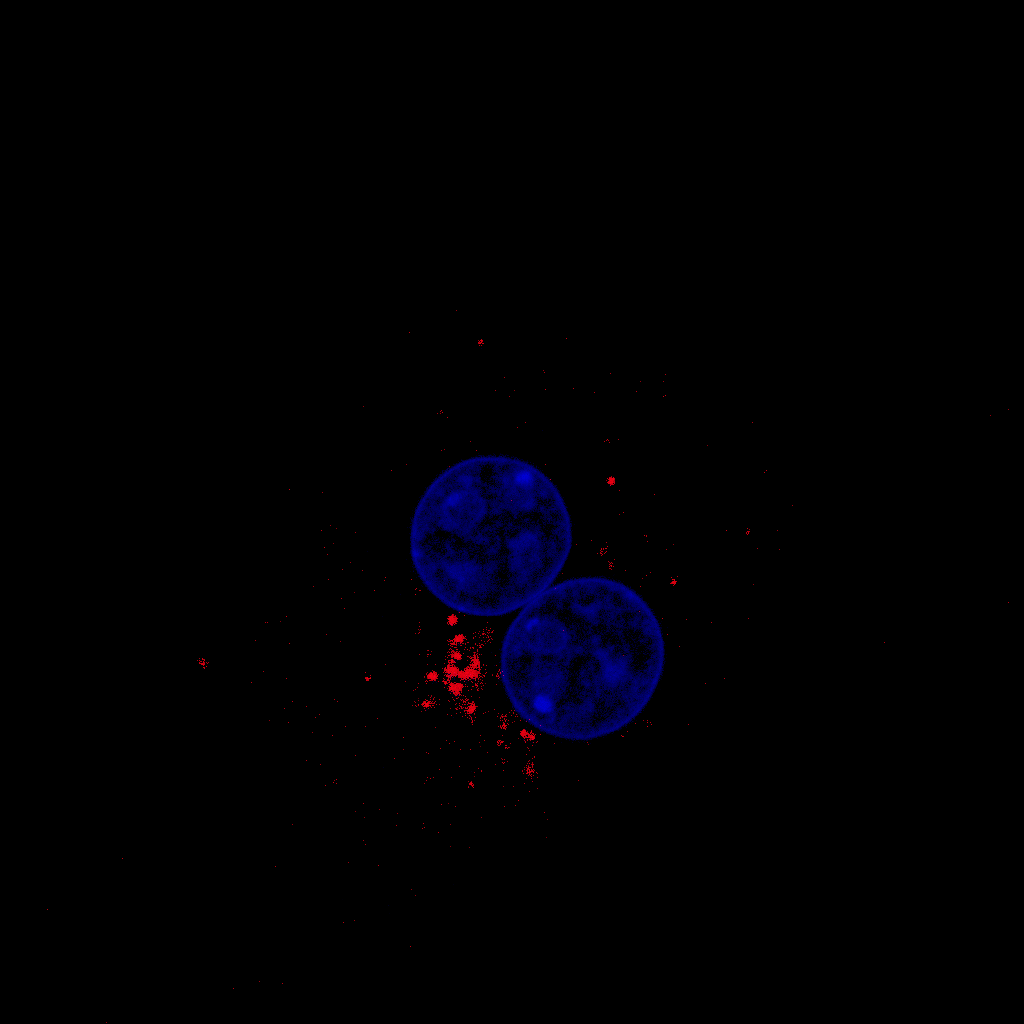

Supplement: Supplementary file 8 — Source data Fig. 2 [file 44321_2024_160_MOESM8_ESM.zip › Figure 2/2B/csa-DEHP.tif]

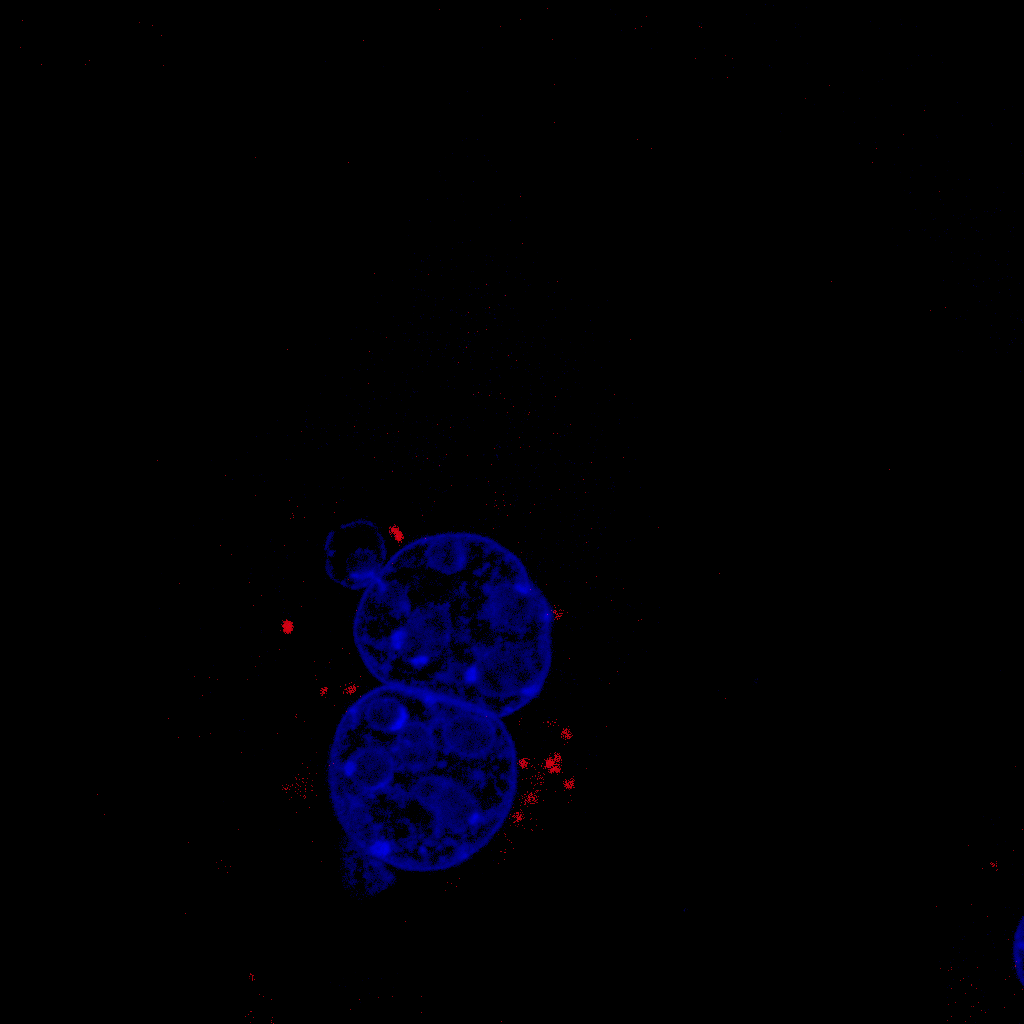

Supplement: Supplementary file 8 — Source data Fig. 2 [file 44321_2024_160_MOESM8_ESM.zip › Figure 2/2B/Cy5+luteolin.tif]

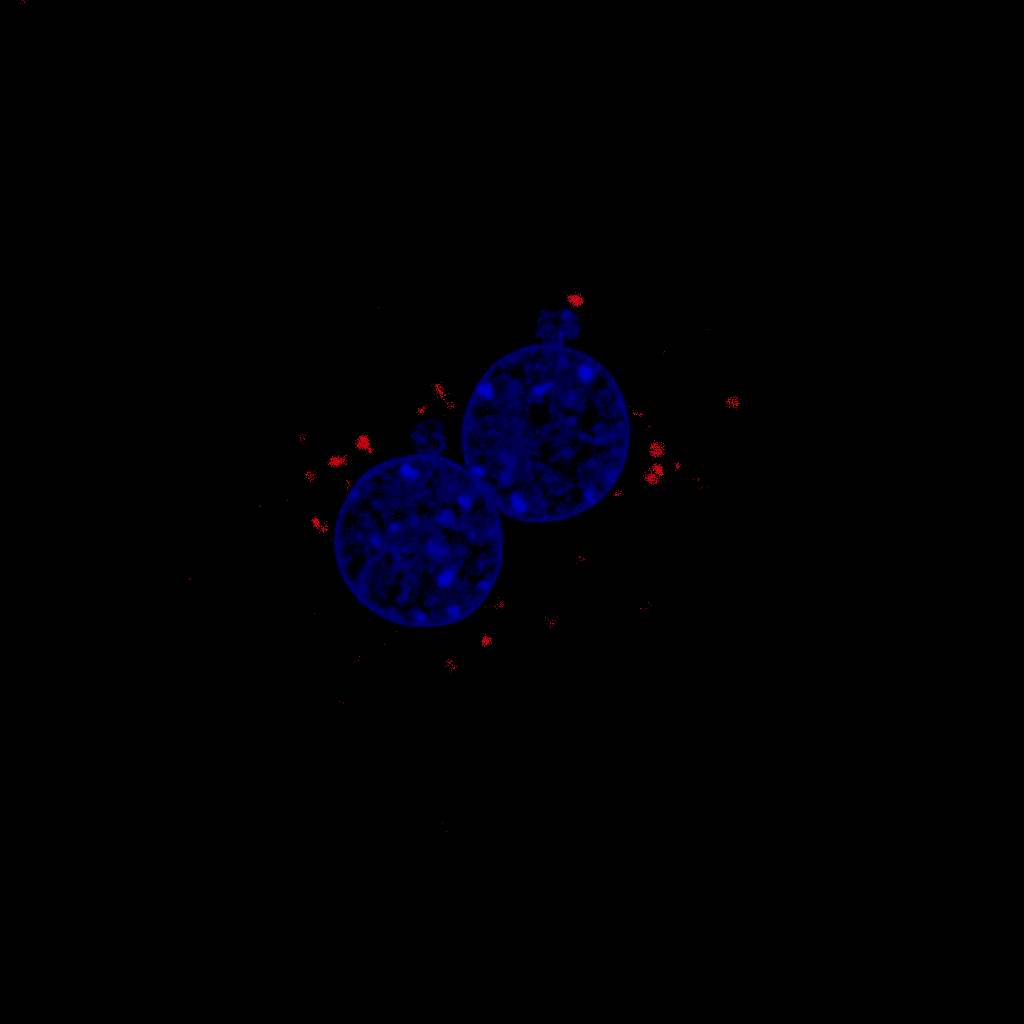

Supplement: Supplementary file 8 — Source data Fig. 2 [file 44321_2024_160_MOESM8_ESM.zip › Figure 2/2B/Cy5.tif]

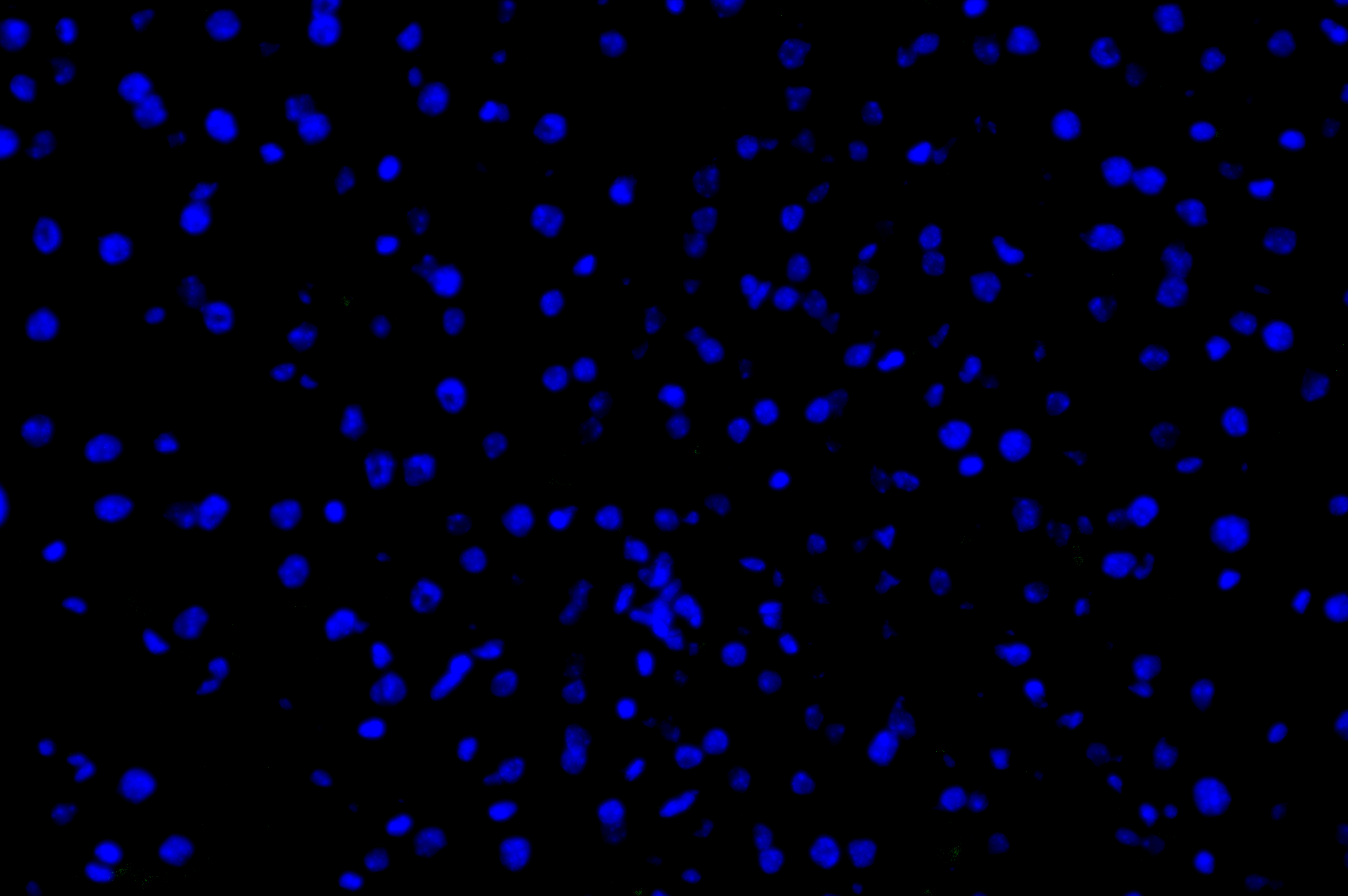

Supplement: Supplementary file 8 — Source data Fig. 2 [file 44321_2024_160_MOESM8_ESM.zip › Figure 2/2F/CTL.tif]

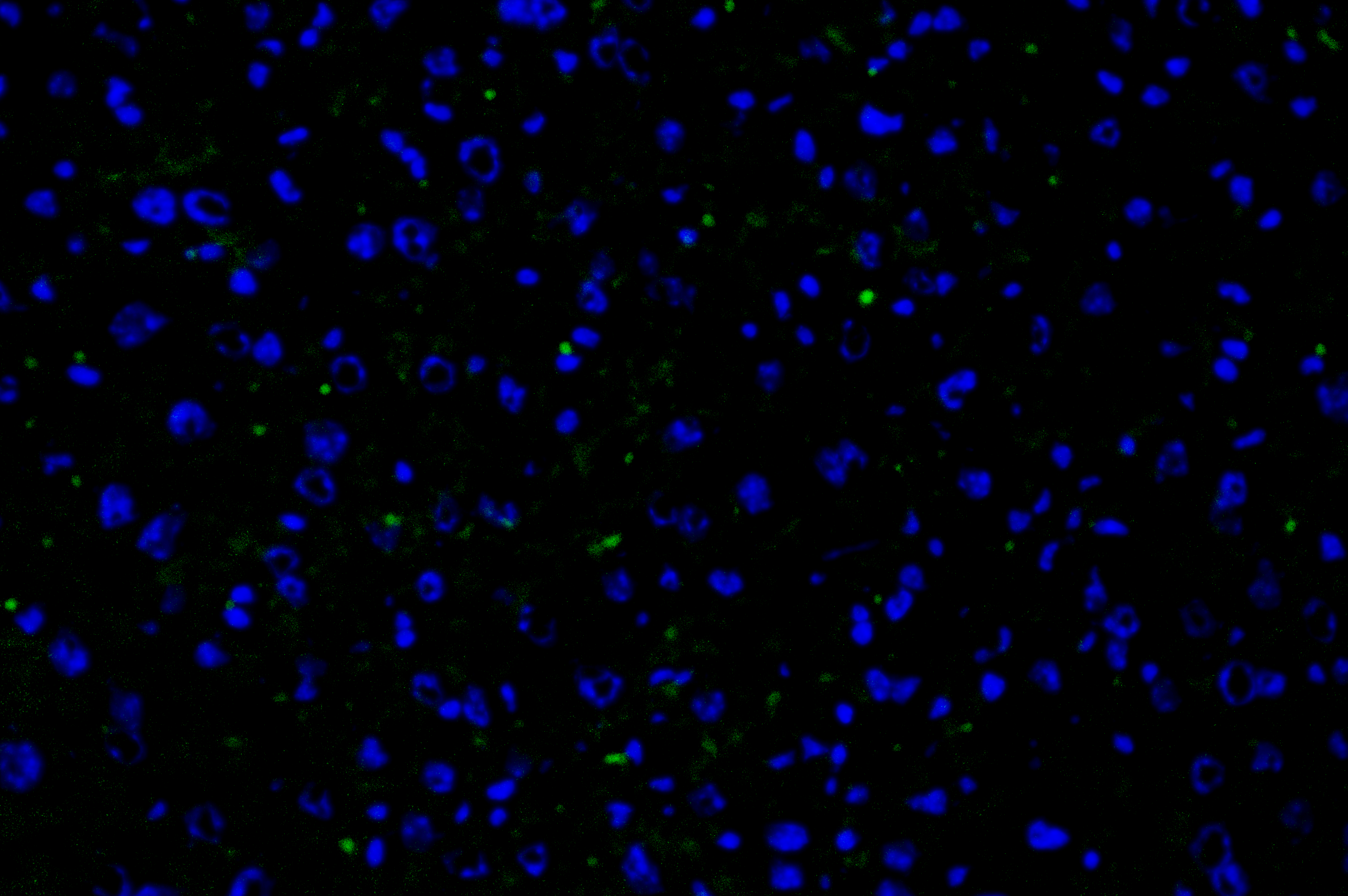

Supplement: Supplementary file 8 — Source data Fig. 2 [file 44321_2024_160_MOESM8_ESM.zip › Figure 2/2F/DEHP+Luteolin.tif]

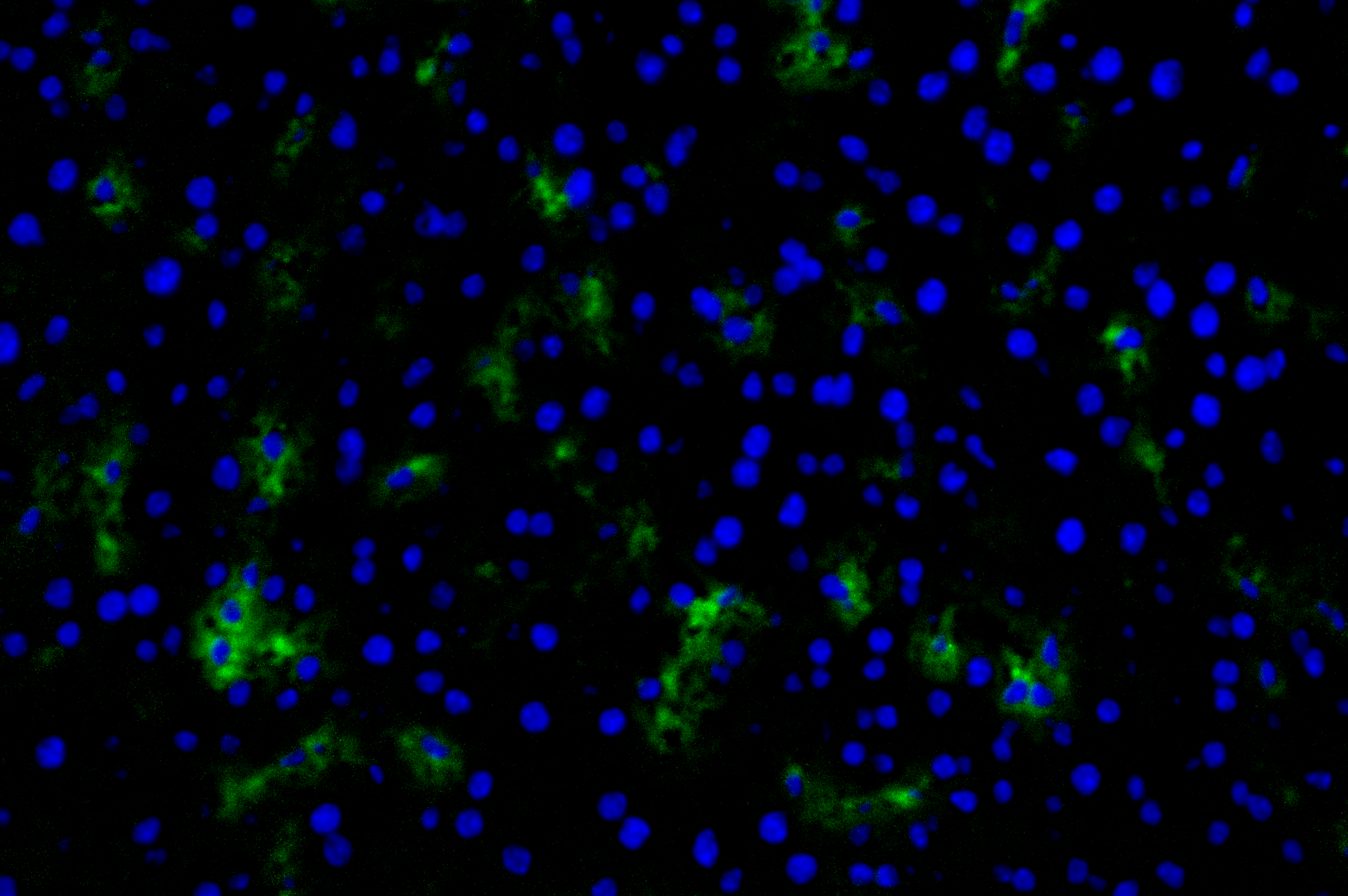

Supplement: Supplementary file 8 — Source data Fig. 2 [file 44321_2024_160_MOESM8_ESM.zip › Figure 2/2F/DEHP.tif]

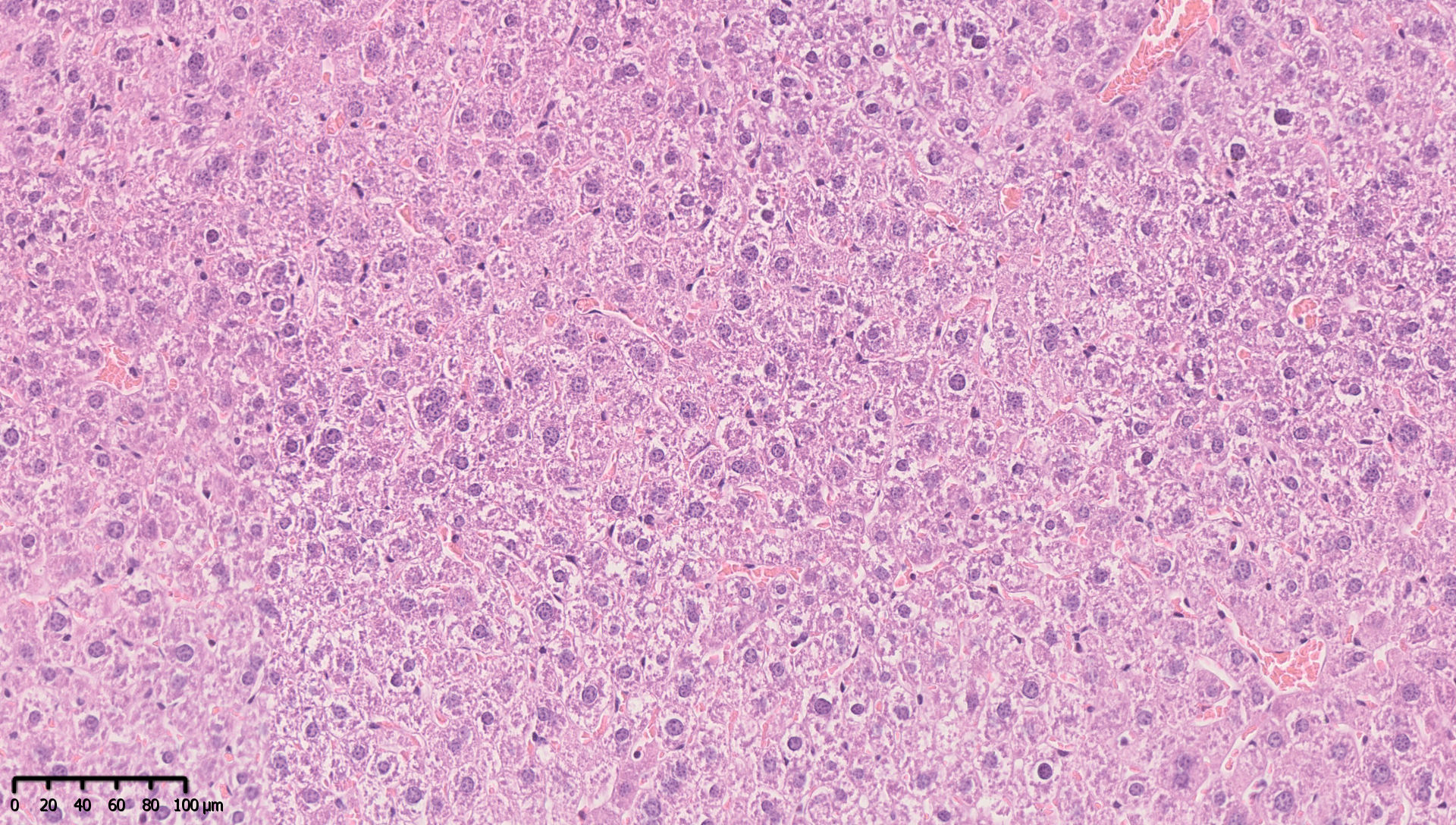

Supplement: Supplementary file 8 — Source data Fig. 2 [file 44321_2024_160_MOESM8_ESM.zip › Figure 2/2G/CTL.jpg]

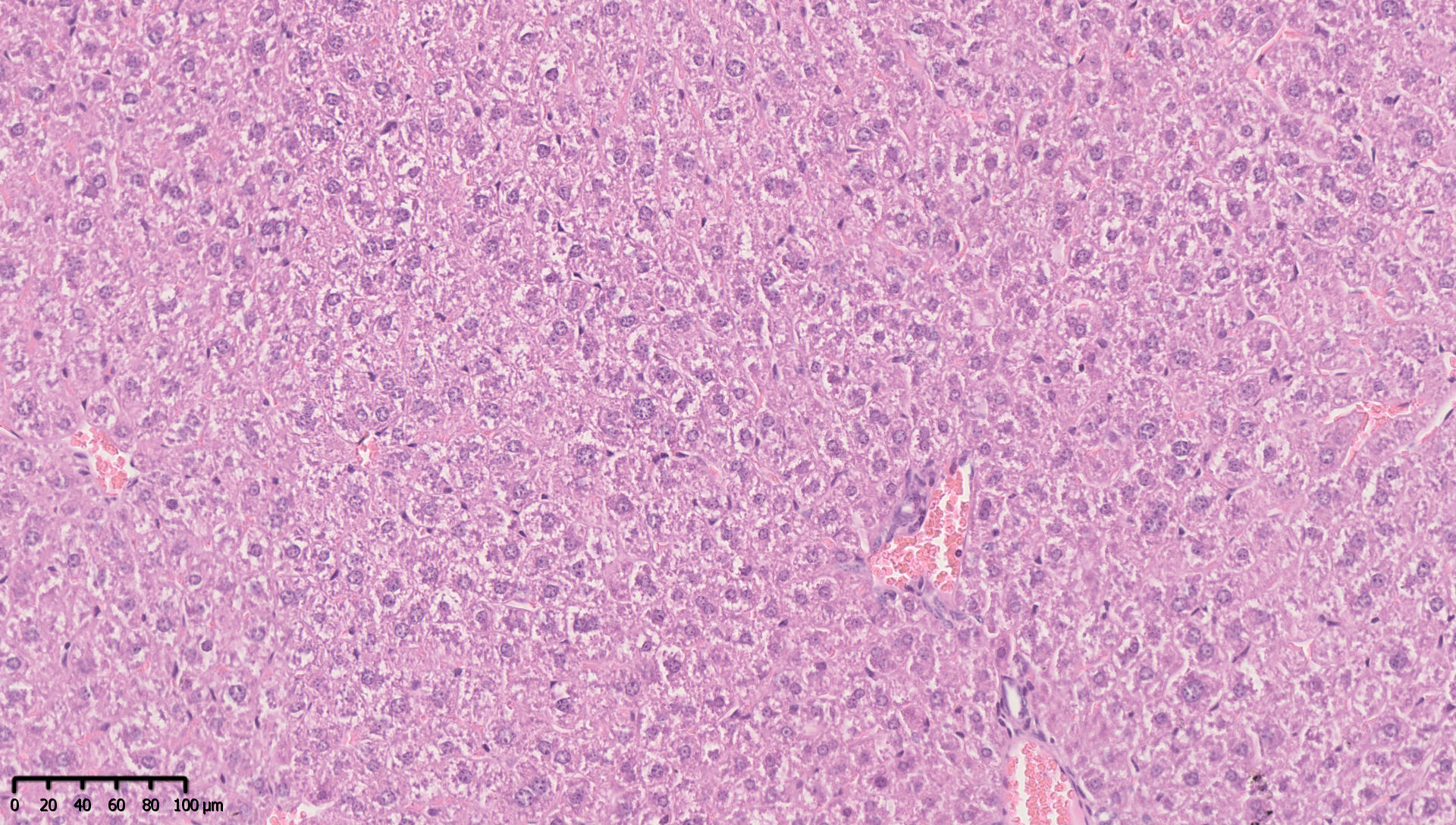

Supplement: Supplementary file 8 — Source data Fig. 2 [file 44321_2024_160_MOESM8_ESM.zip › Figure 2/2G/DEHP+Luteolin.jpg]

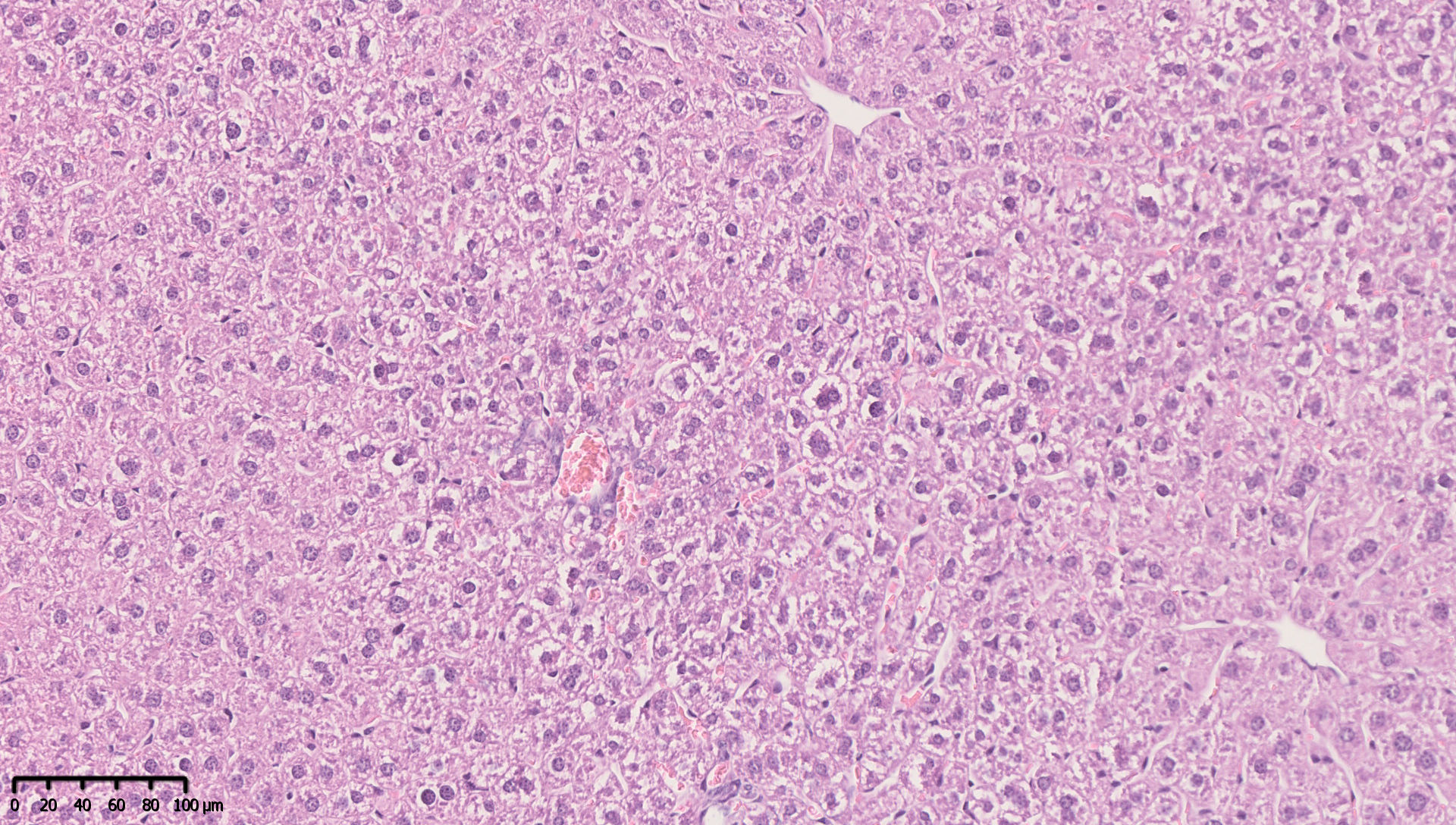

Supplement: Supplementary file 8 — Source data Fig. 2 [file 44321_2024_160_MOESM8_ESM.zip › Figure 2/2G/DEHP.jpg]

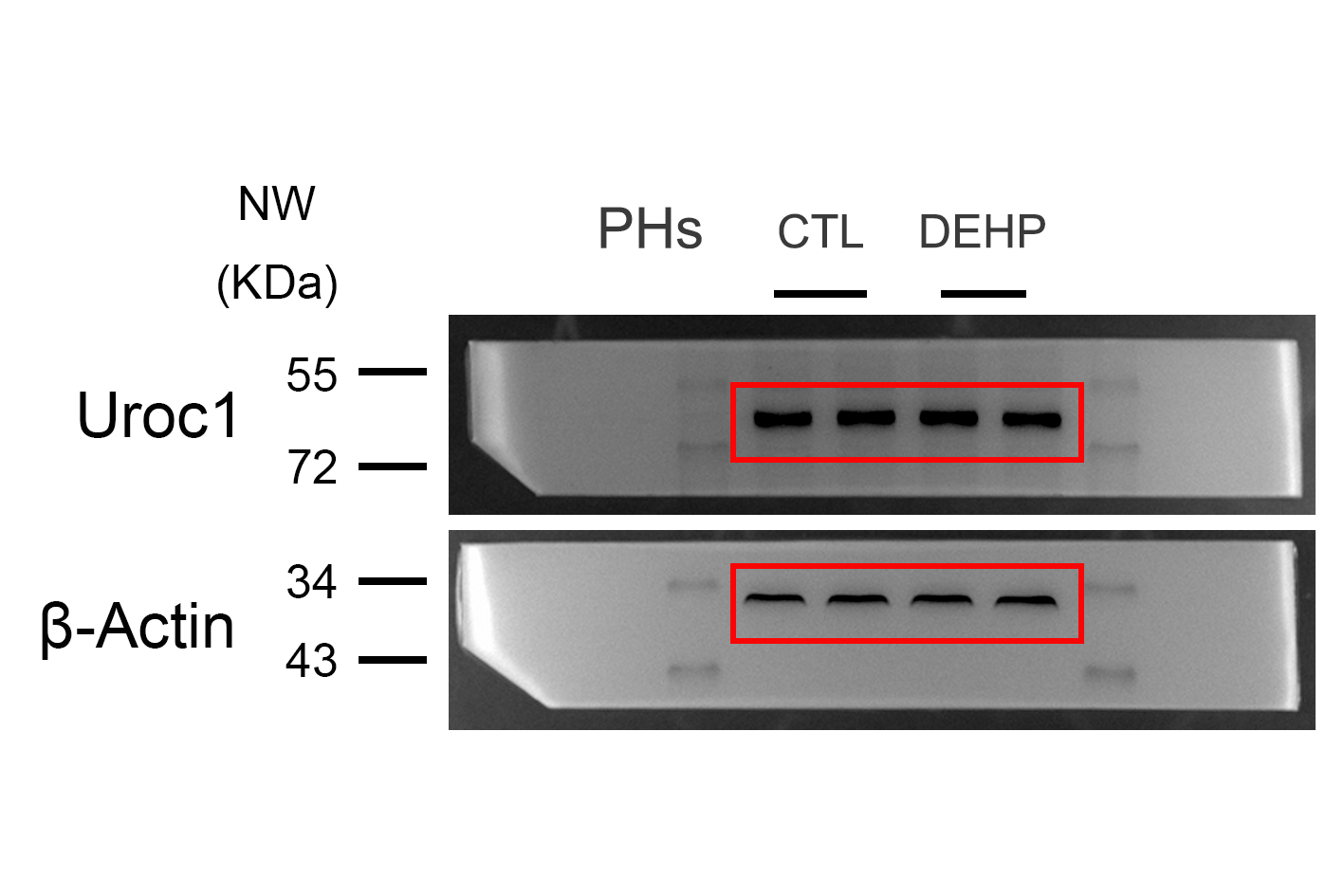

Supplement: Supplementary file 9 — Source data Fig. 3 [file 44321_2024_160_MOESM9_ESM.zip › Figure 3/3F/PHs CTL DEHP WB.tif]

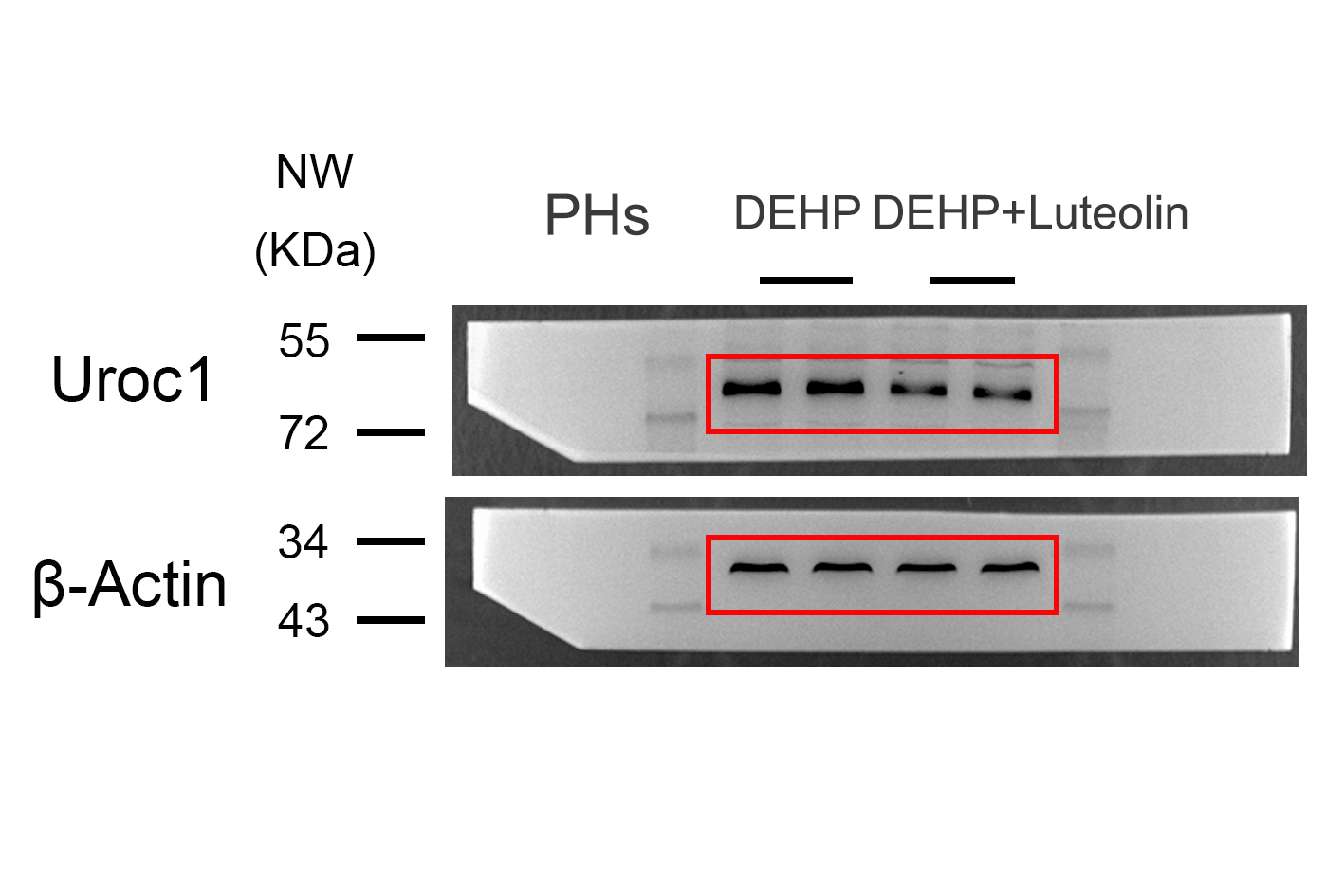

Supplement: Supplementary file 9 — Source data Fig. 3 [file 44321_2024_160_MOESM9_ESM.zip › Figure 3/3G/PHs DEHP DEHP+ Luteolin WB.tif]

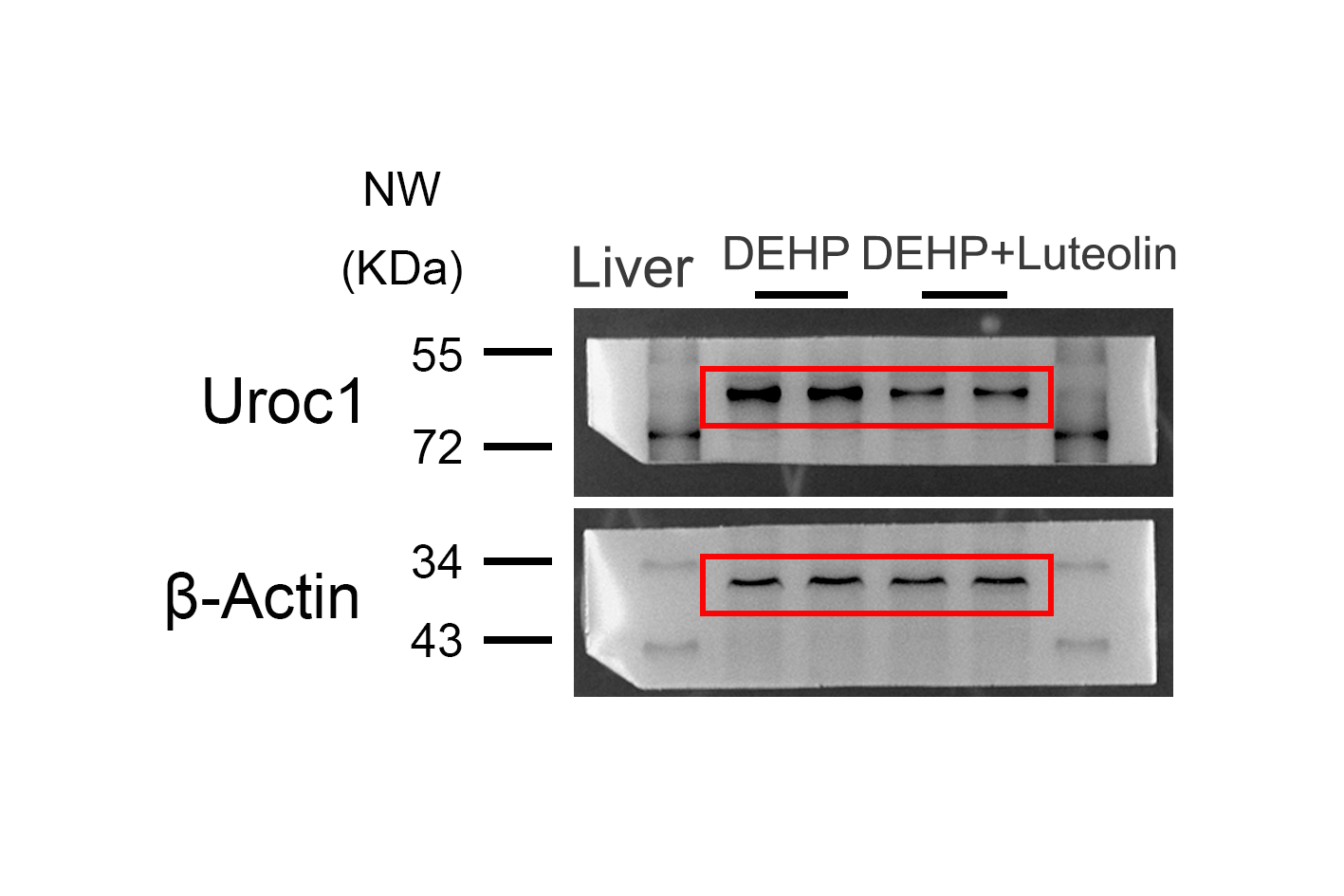

Supplement: Supplementary file 9 — Source data Fig. 3 [file 44321_2024_160_MOESM9_ESM.zip › Figure 3/3H/Liver DEHP DEHP+ Luteolin WB.tif]

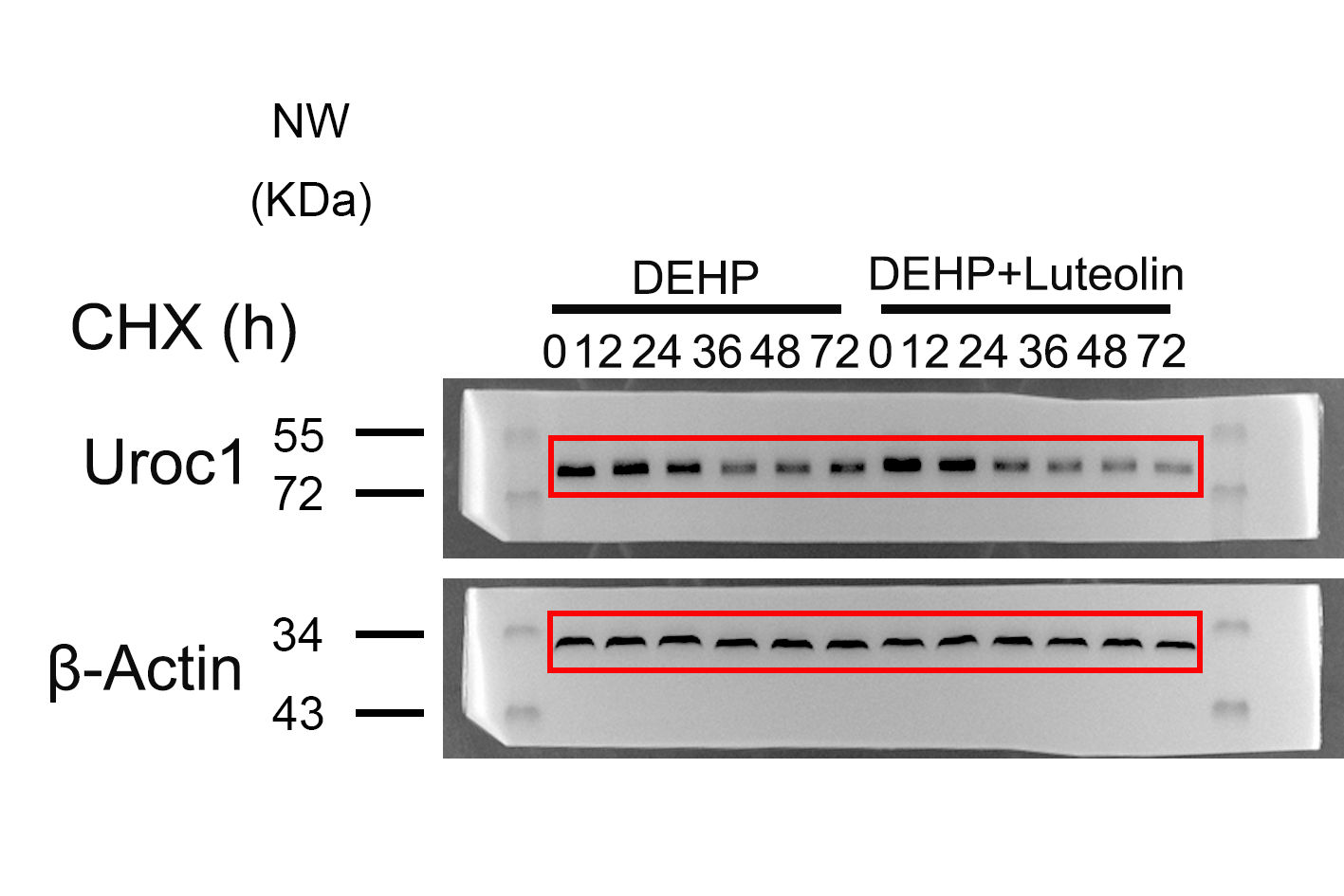

Supplement: Supplementary file 9 — Source data Fig. 3 [file 44321_2024_160_MOESM9_ESM.zip › Figure 3/3I/3i WB.tif]

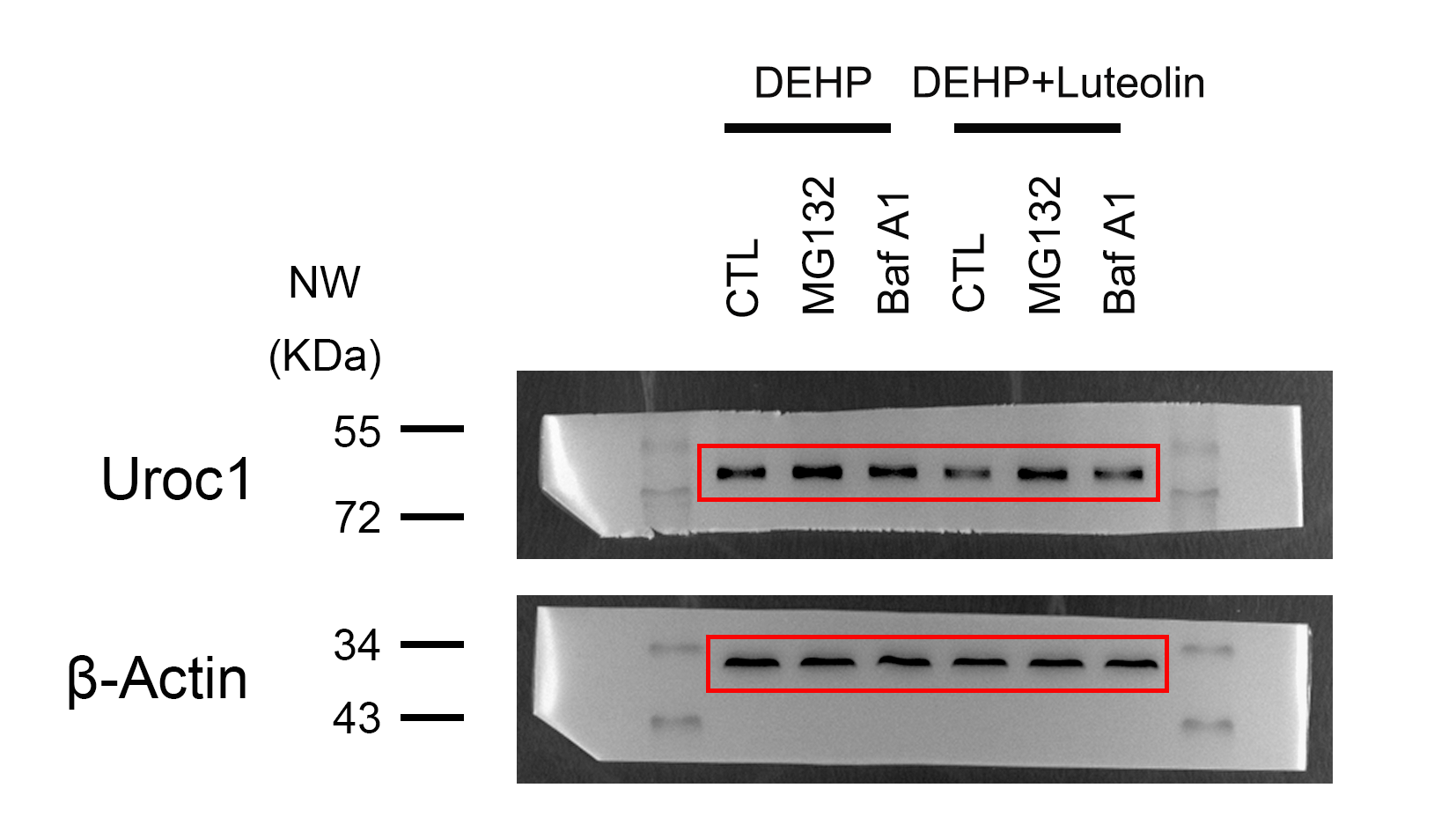

Supplement: Supplementary file 10 — Source data Fig. 4 [file 44321_2024_160_MOESM10_ESM.zip › Figure 4/4A/Fig 4A.tif]

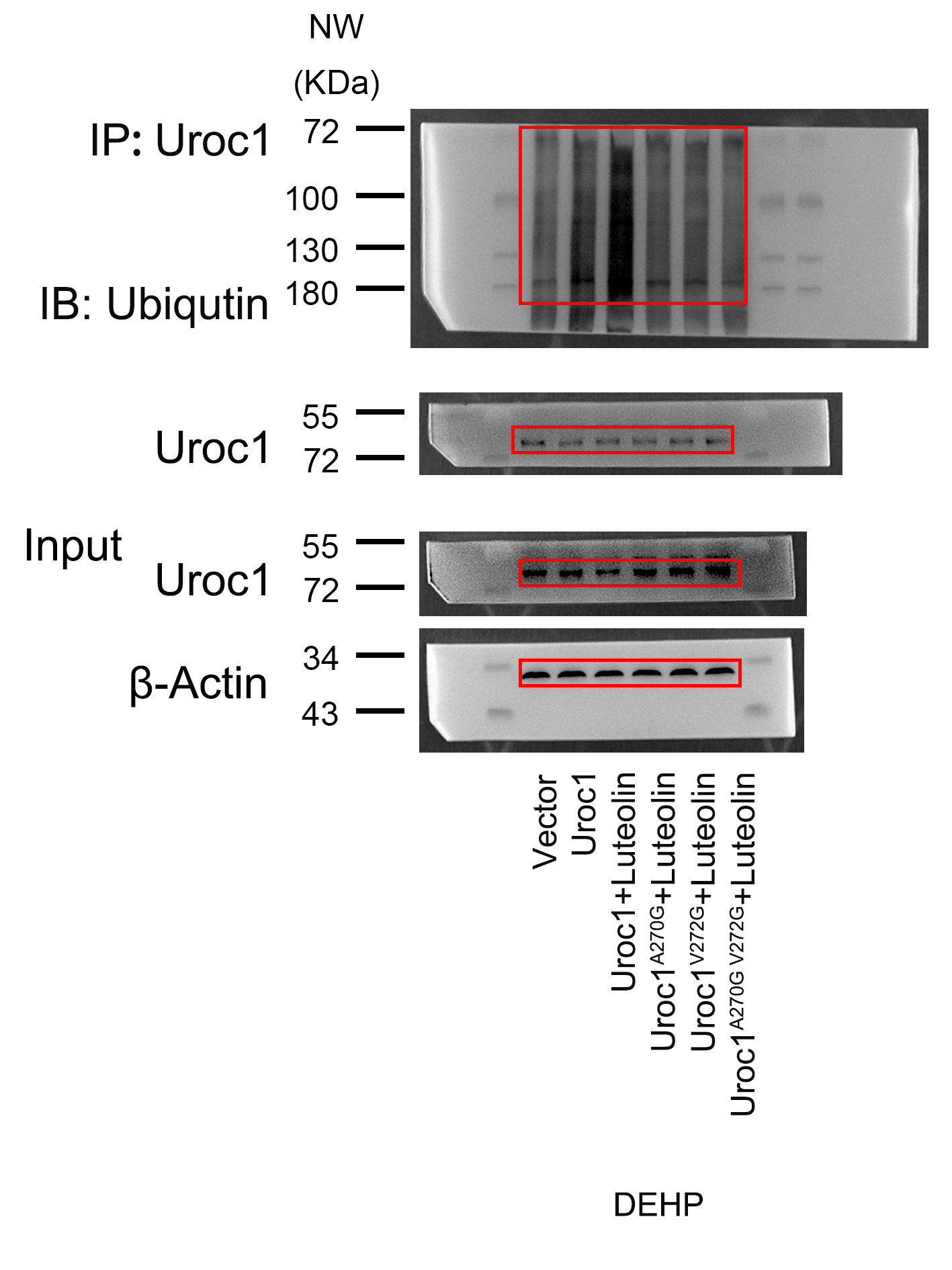

Supplement: Supplementary file 10 — Source data Fig. 4 [file 44321_2024_160_MOESM10_ESM.zip › Figure 4/4C/4C.tif]

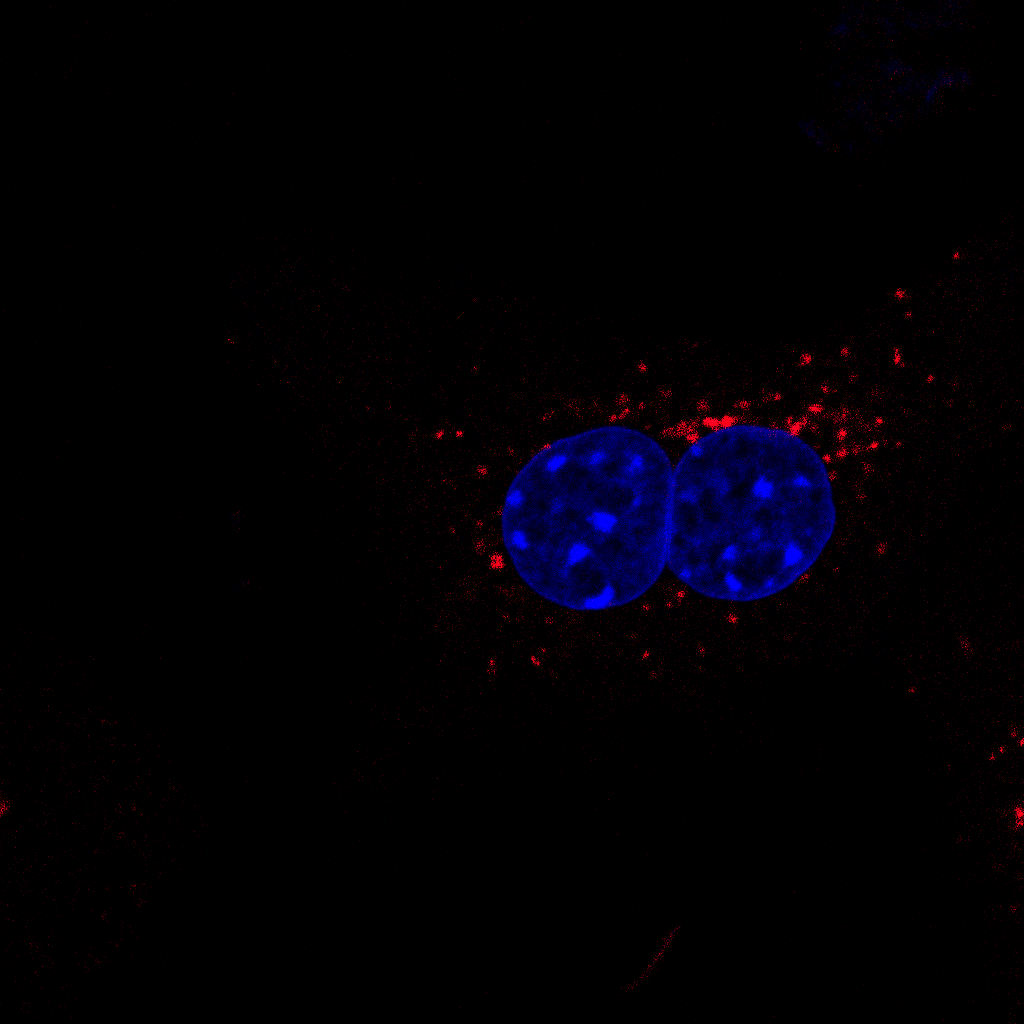

Supplement: Supplementary file 10 — Source data Fig. 4 [file 44321_2024_160_MOESM10_ESM.zip › Figure 4/4D/Uroc1 A270G V272G+Luteolin.tif]

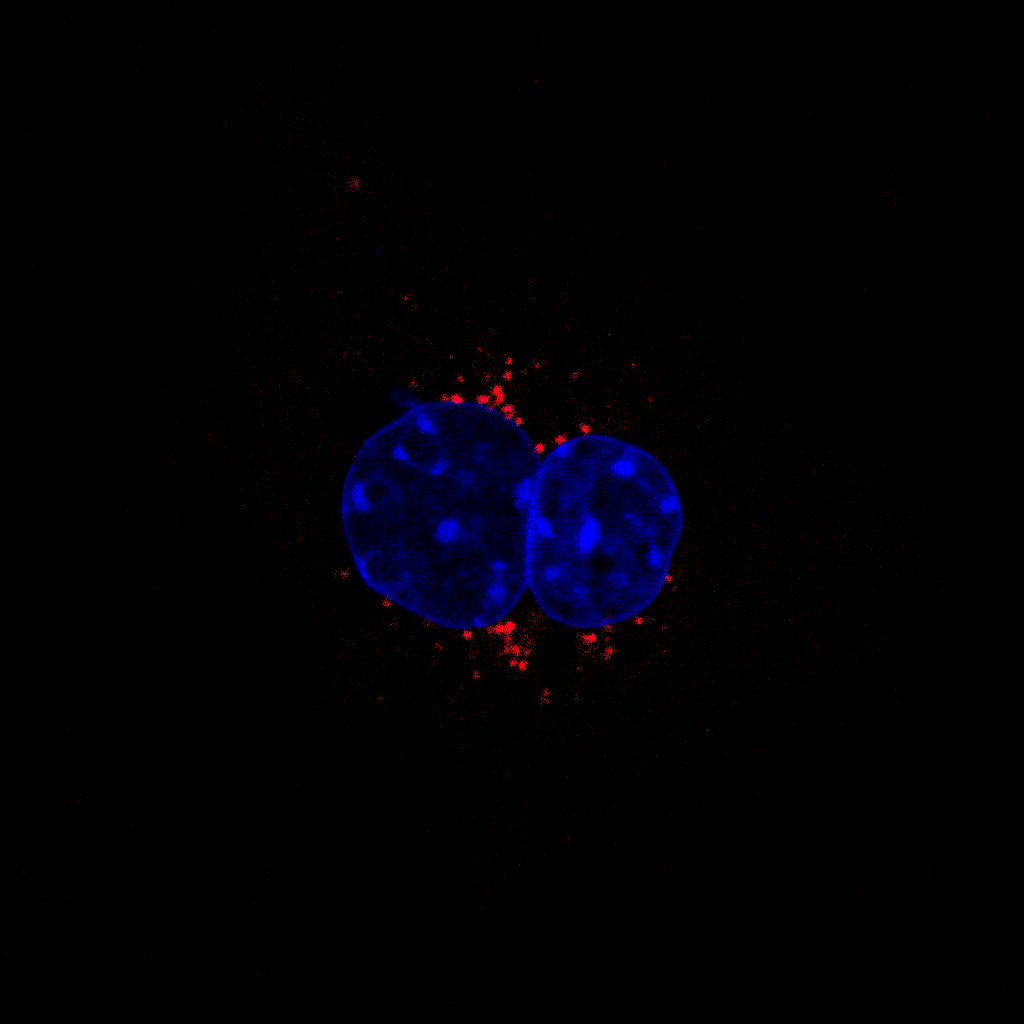

Supplement: Supplementary file 10 — Source data Fig. 4 [file 44321_2024_160_MOESM10_ESM.zip › Figure 4/4D/Uroc1 A270G+Luteolin.tif]

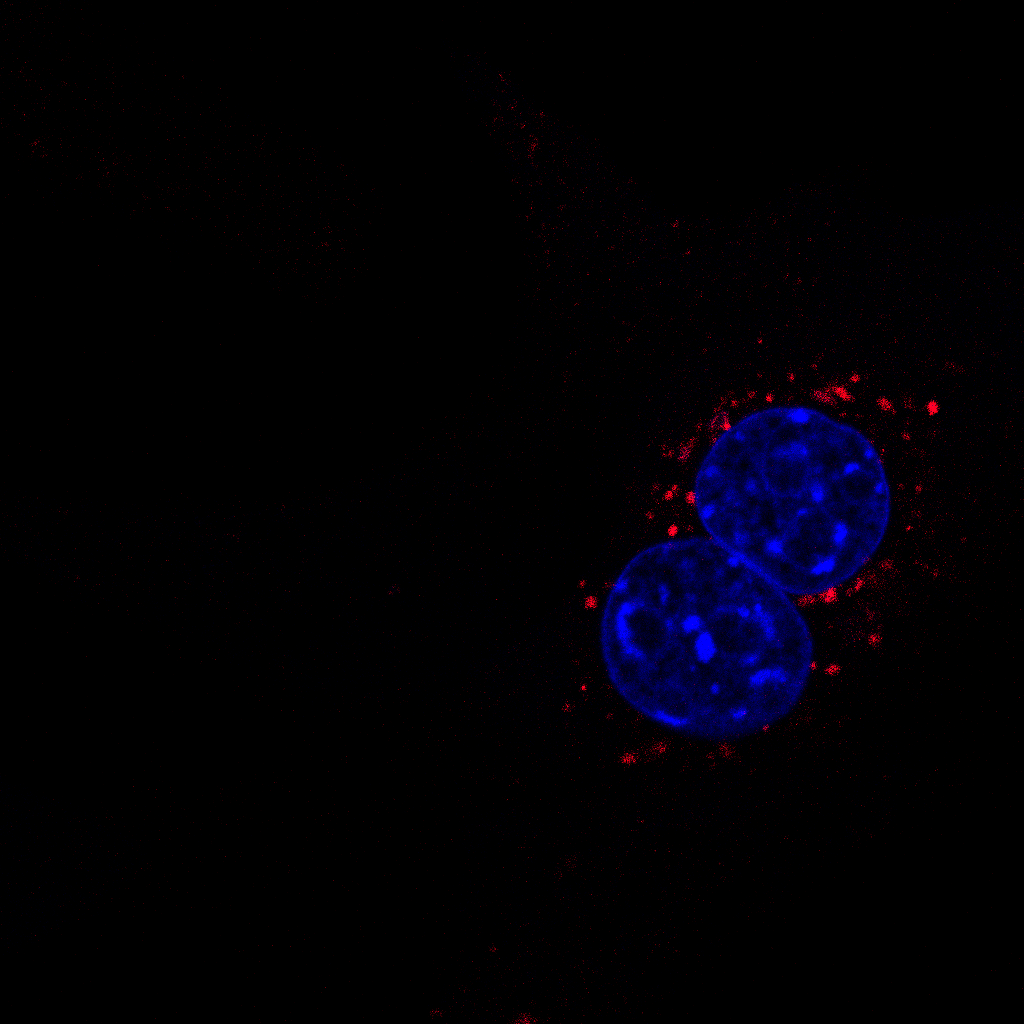

Supplement: Supplementary file 10 — Source data Fig. 4 [file 44321_2024_160_MOESM10_ESM.zip › Figure 4/4D/Uroc1 V272G+Luteolin.tif]

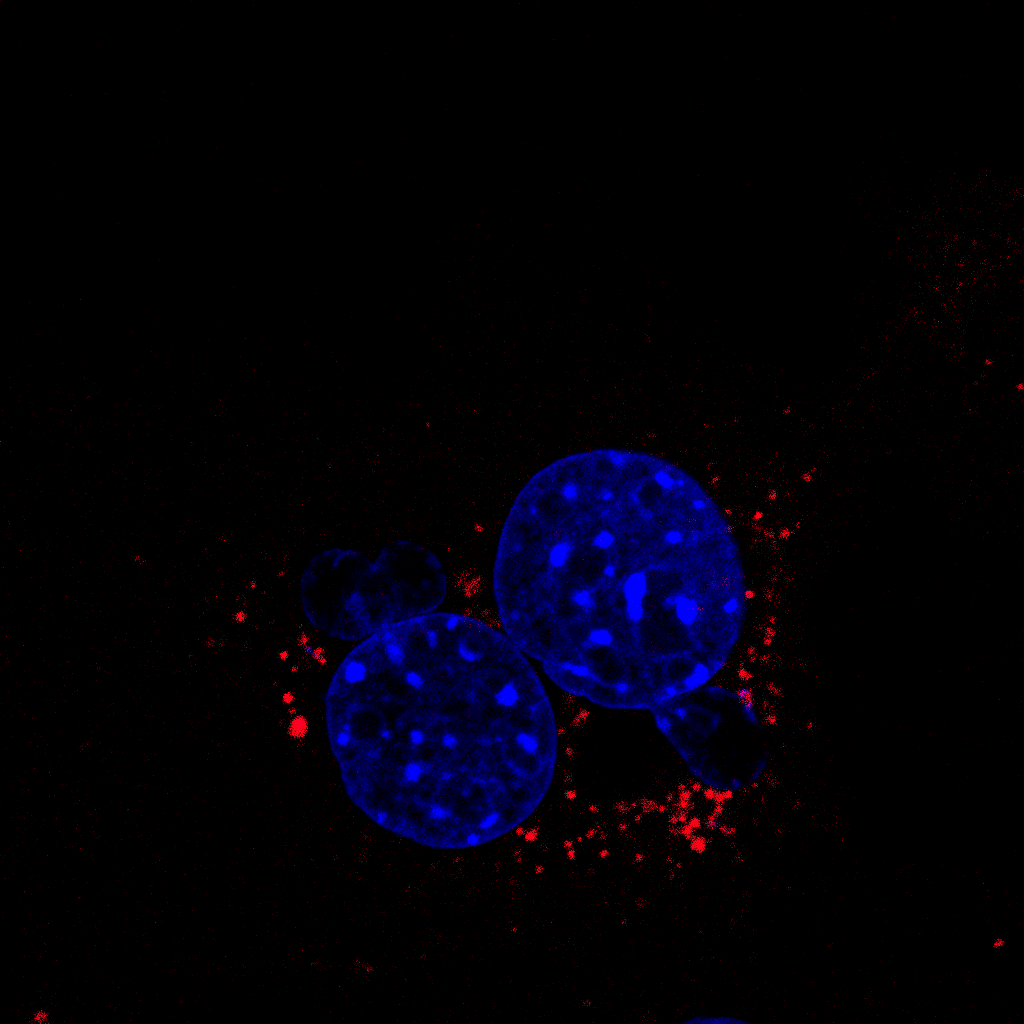

Supplement: Supplementary file 10 — Source data Fig. 4 [file 44321_2024_160_MOESM10_ESM.zip › Figure 4/4D/Uroc1+Luteolin.tif]

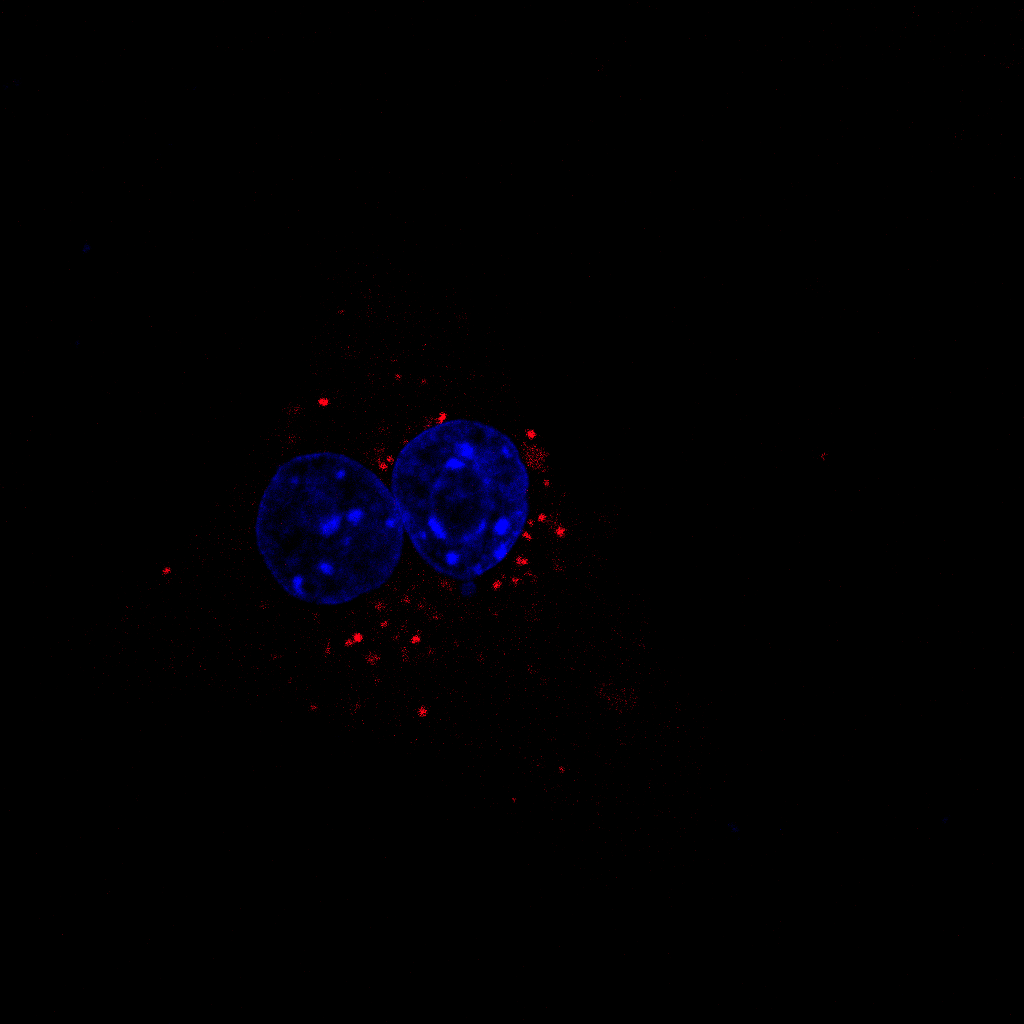

Supplement: Supplementary file 10 — Source data Fig. 4 [file 44321_2024_160_MOESM10_ESM.zip › Figure 4/4D/Vector+Luteolin.tif]

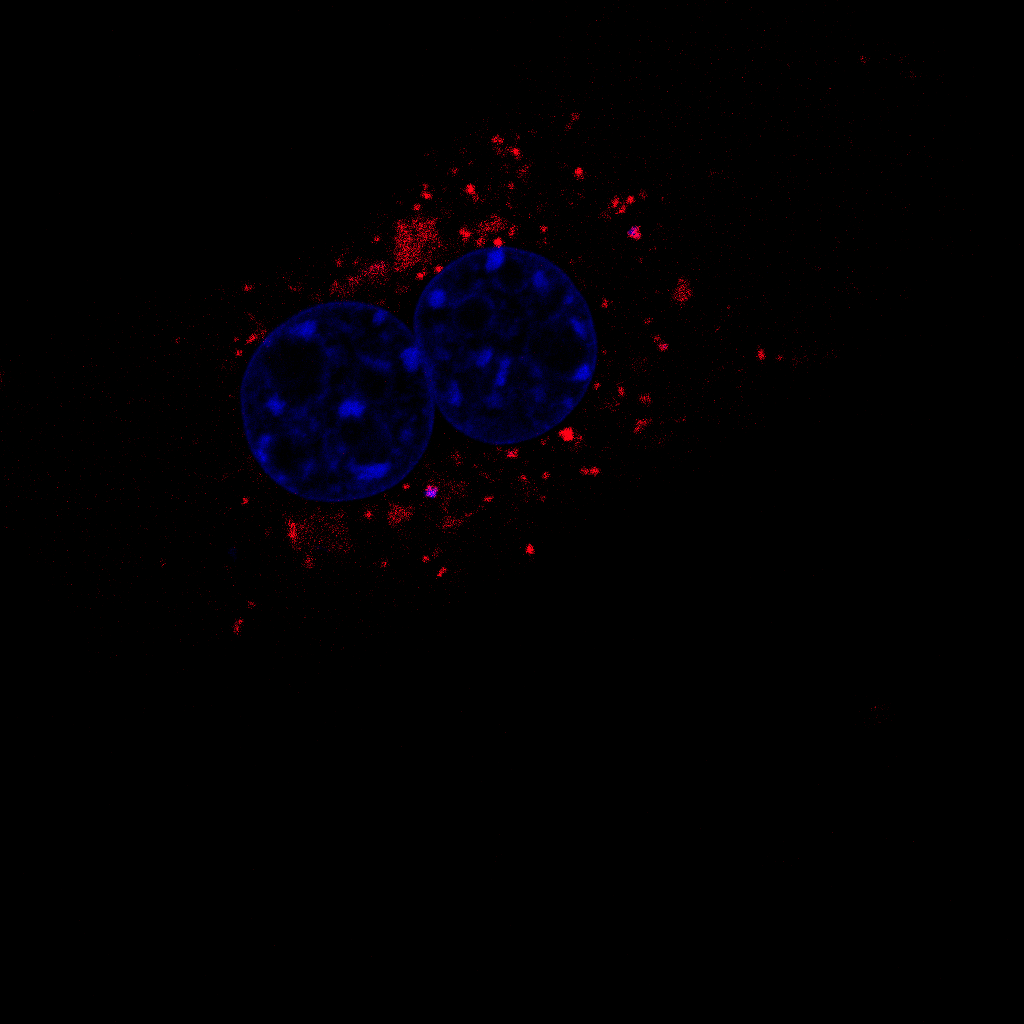

Supplement: Supplementary file 10 — Source data Fig. 4 [file 44321_2024_160_MOESM10_ESM.zip › Figure 4/4D/Vector.tif]

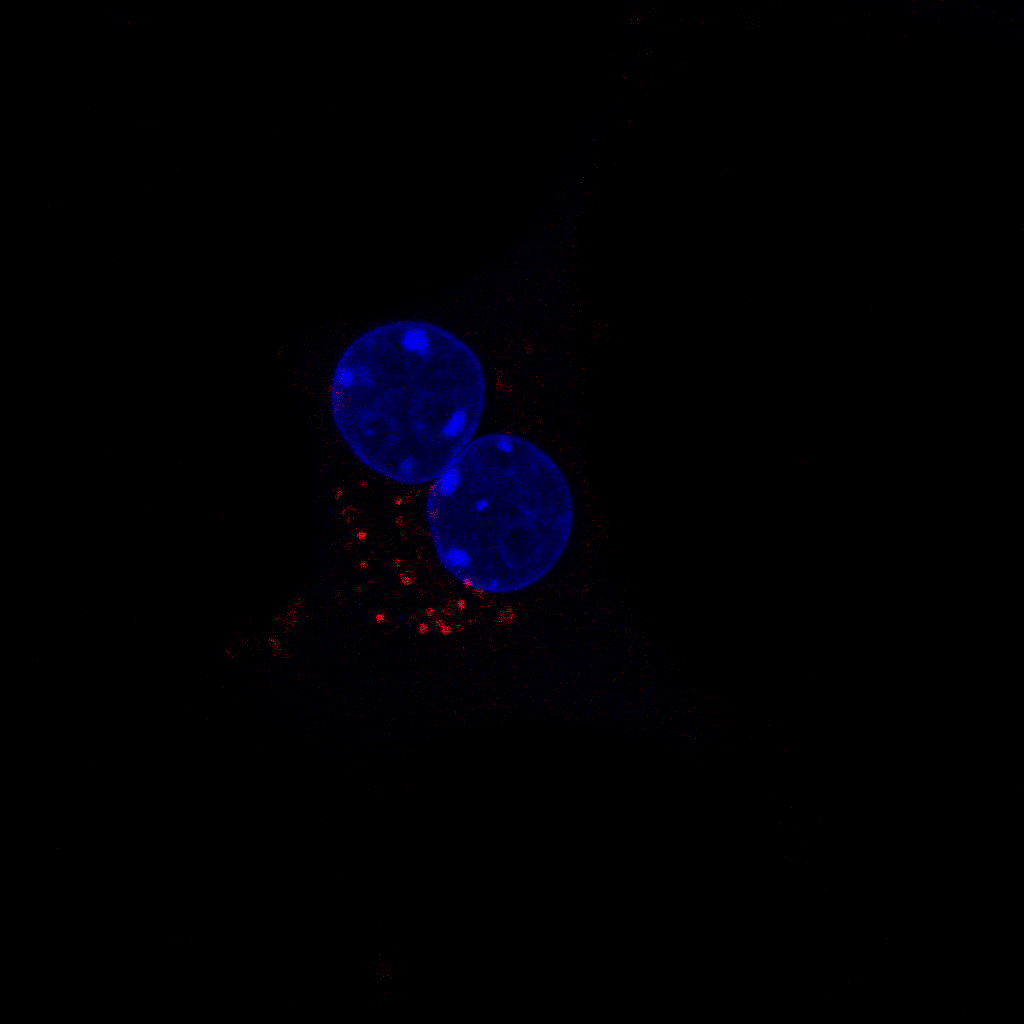

Supplement: Supplementary file 10 — Source data Fig. 4 [file 44321_2024_160_MOESM10_ESM.zip › Figure 4/4E/NC sh RNA Luteolin.tif]

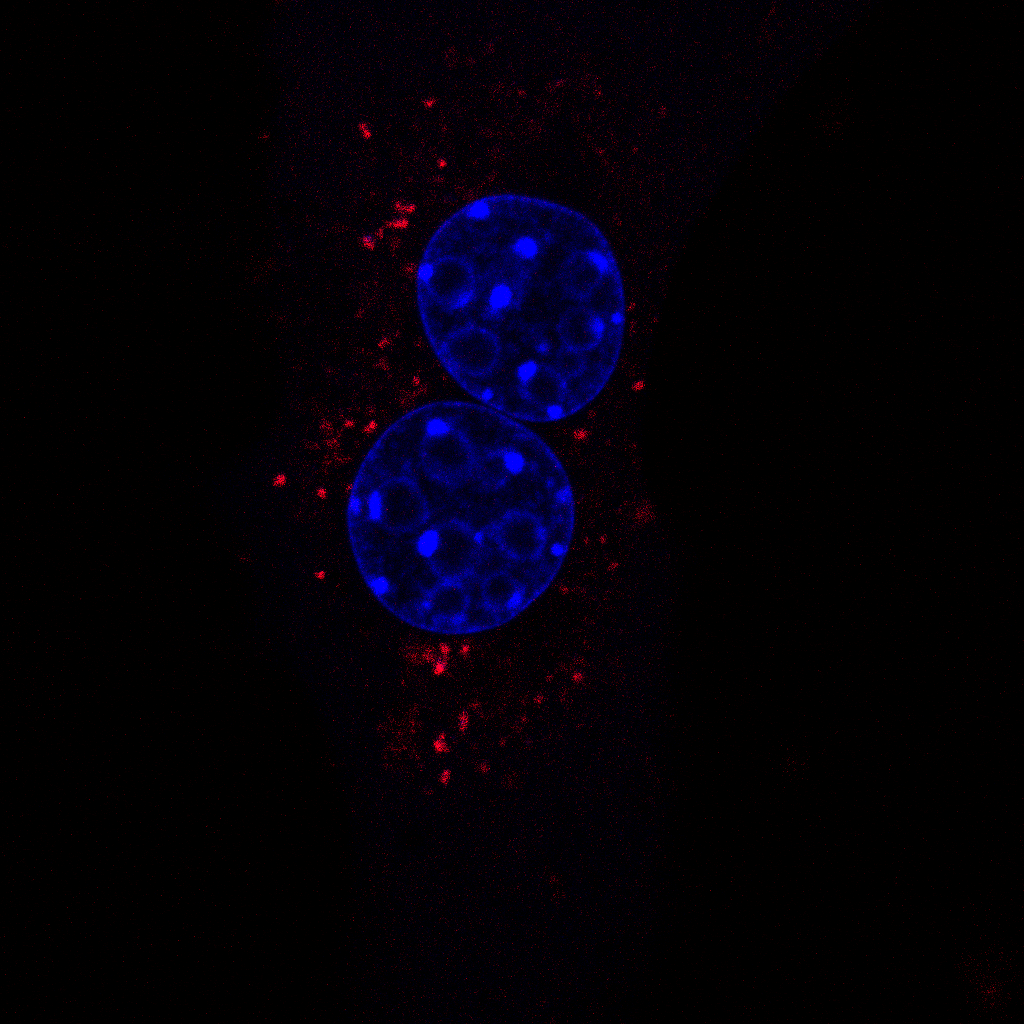

Supplement: Supplementary file 10 — Source data Fig. 4 [file 44321_2024_160_MOESM10_ESM.zip › Figure 4/4E/NC sh RNA CTL.tif]

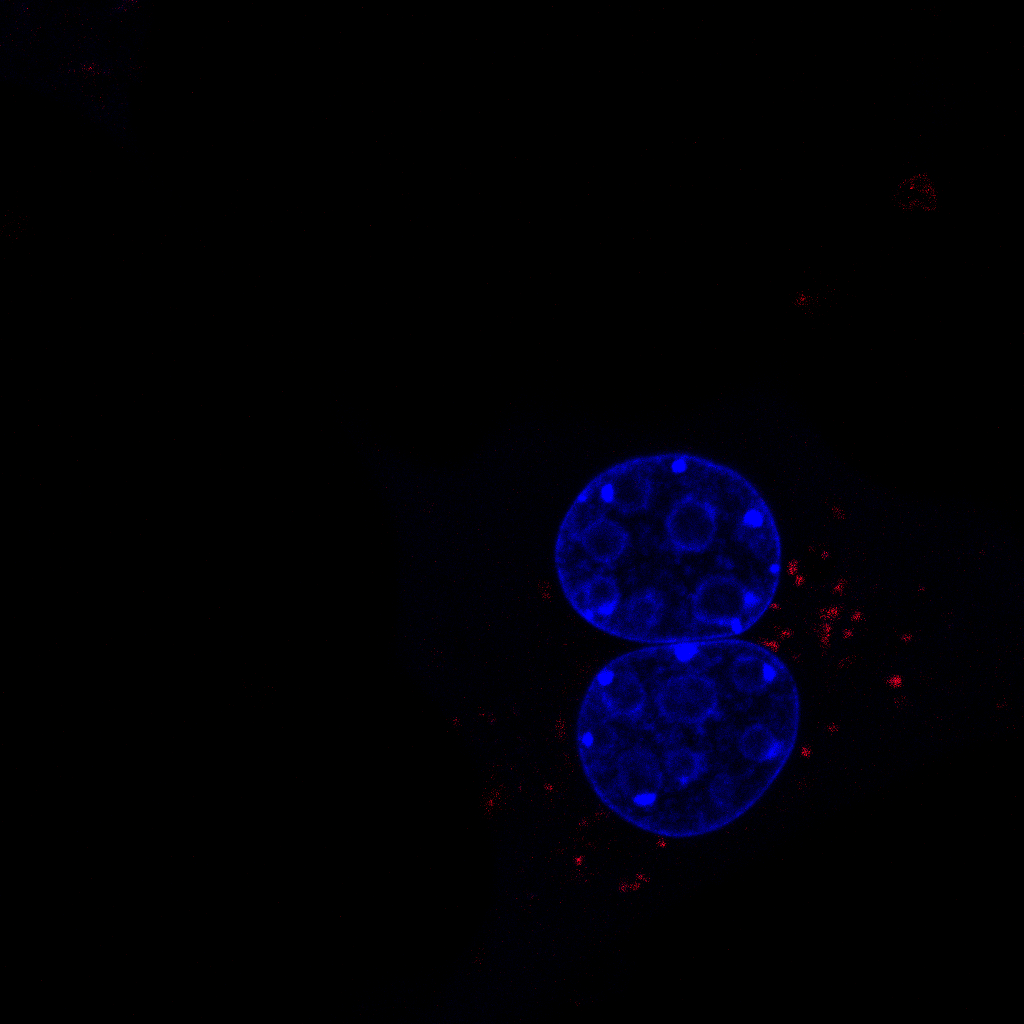

Supplement: Supplementary file 10 — Source data Fig. 4 [file 44321_2024_160_MOESM10_ESM.zip › Figure 4/4E/Uroc1 sh RNA CTL.tif]

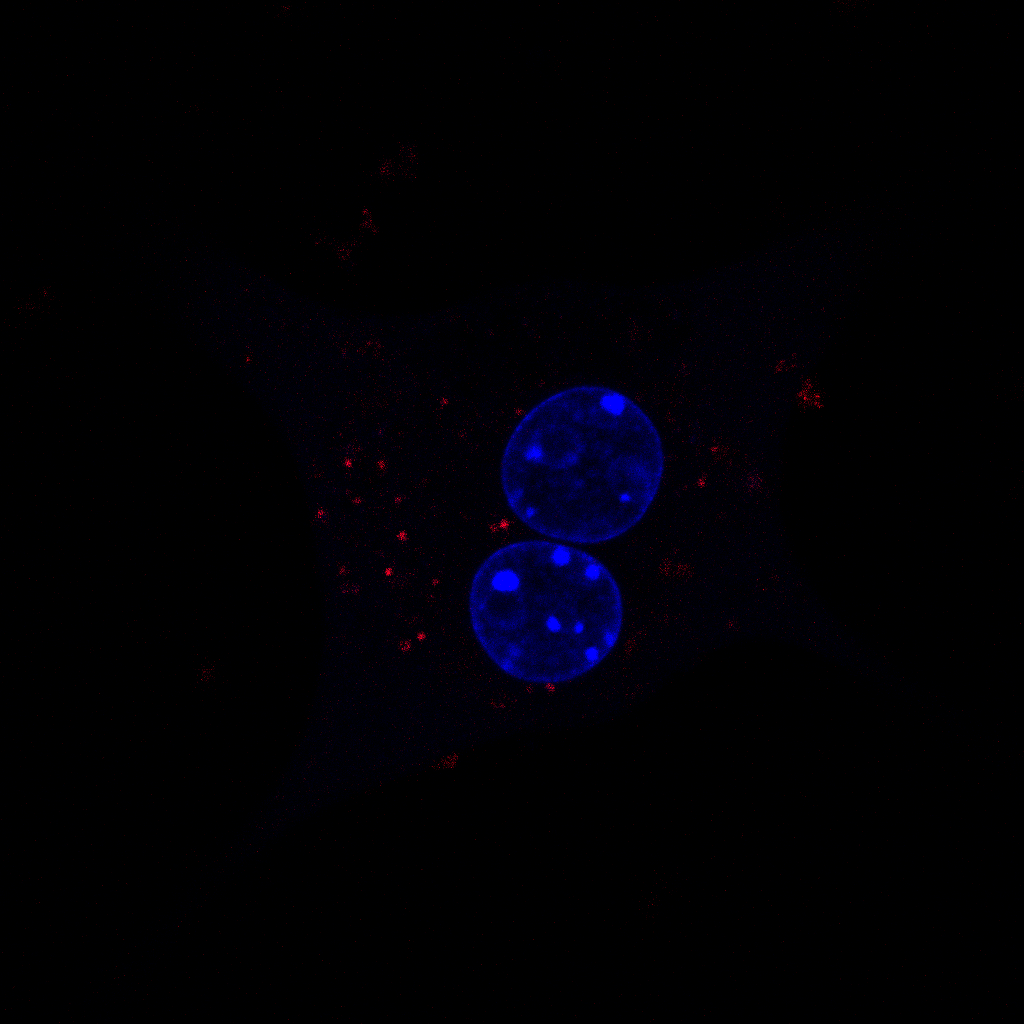

Supplement: Supplementary file 10 — Source data Fig. 4 [file 44321_2024_160_MOESM10_ESM.zip › Figure 4/4E/Uroc1 sh RNA Luteolin.tif]

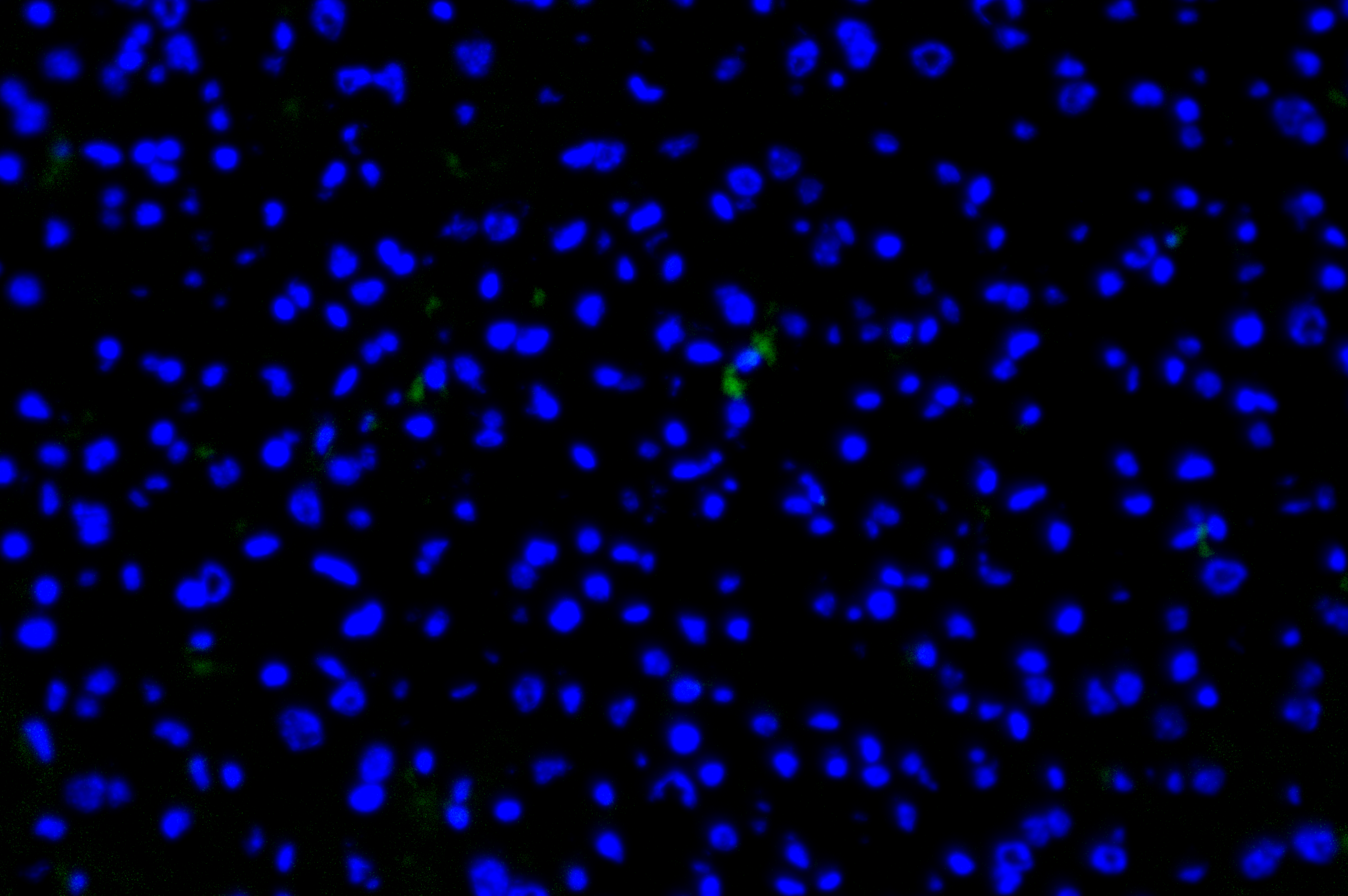

Supplement: Supplementary file 10 — Source data Fig. 4 [file 44321_2024_160_MOESM10_ESM.zip › Figure 4/4I/F480 AAV-TBG+DEHP+Luteolin.tif]

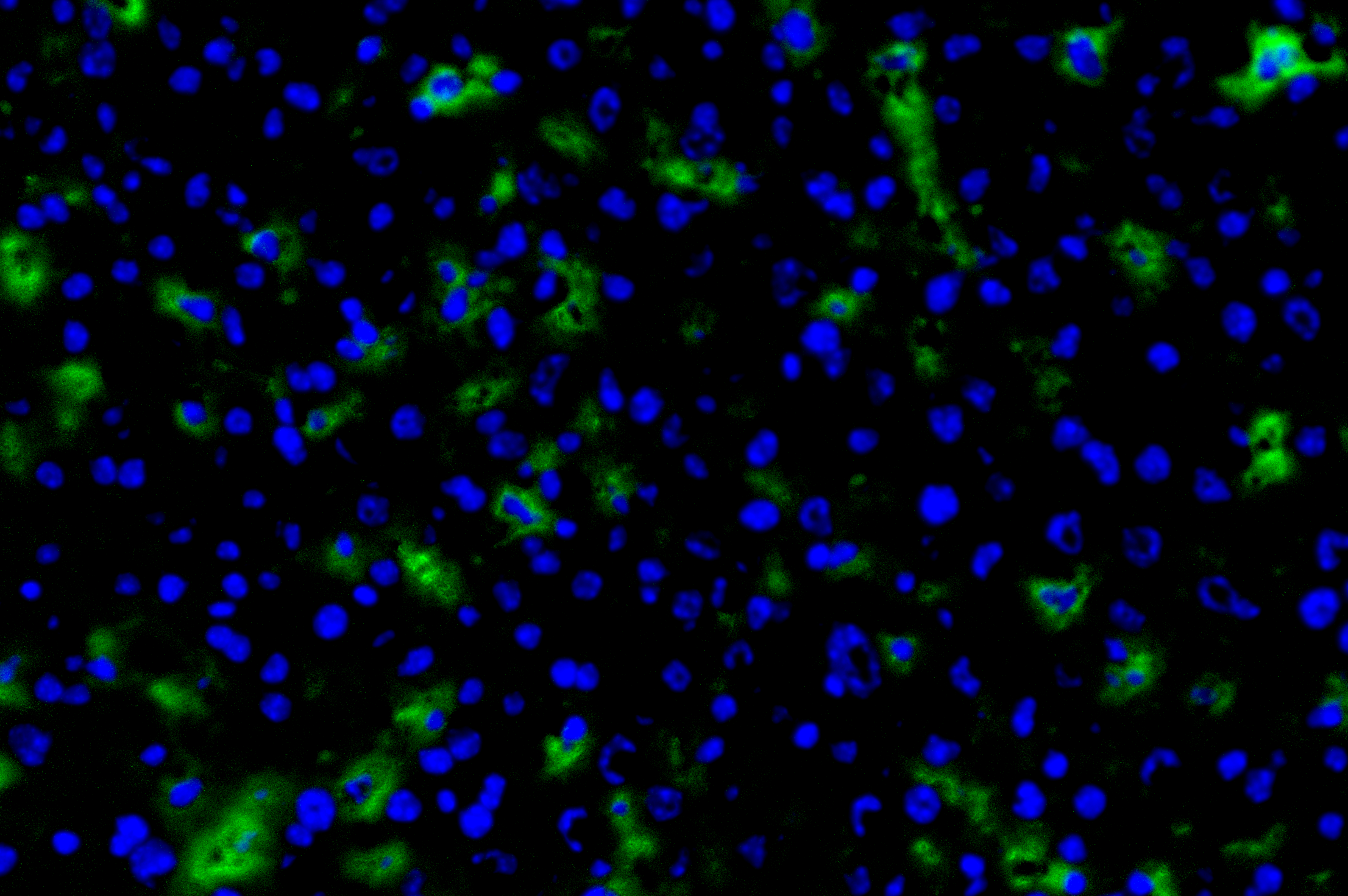

Supplement: Supplementary file 10 — Source data Fig. 4 [file 44321_2024_160_MOESM10_ESM.zip › Figure 4/4I/F480 AAV-TBG+DEHP.tif]

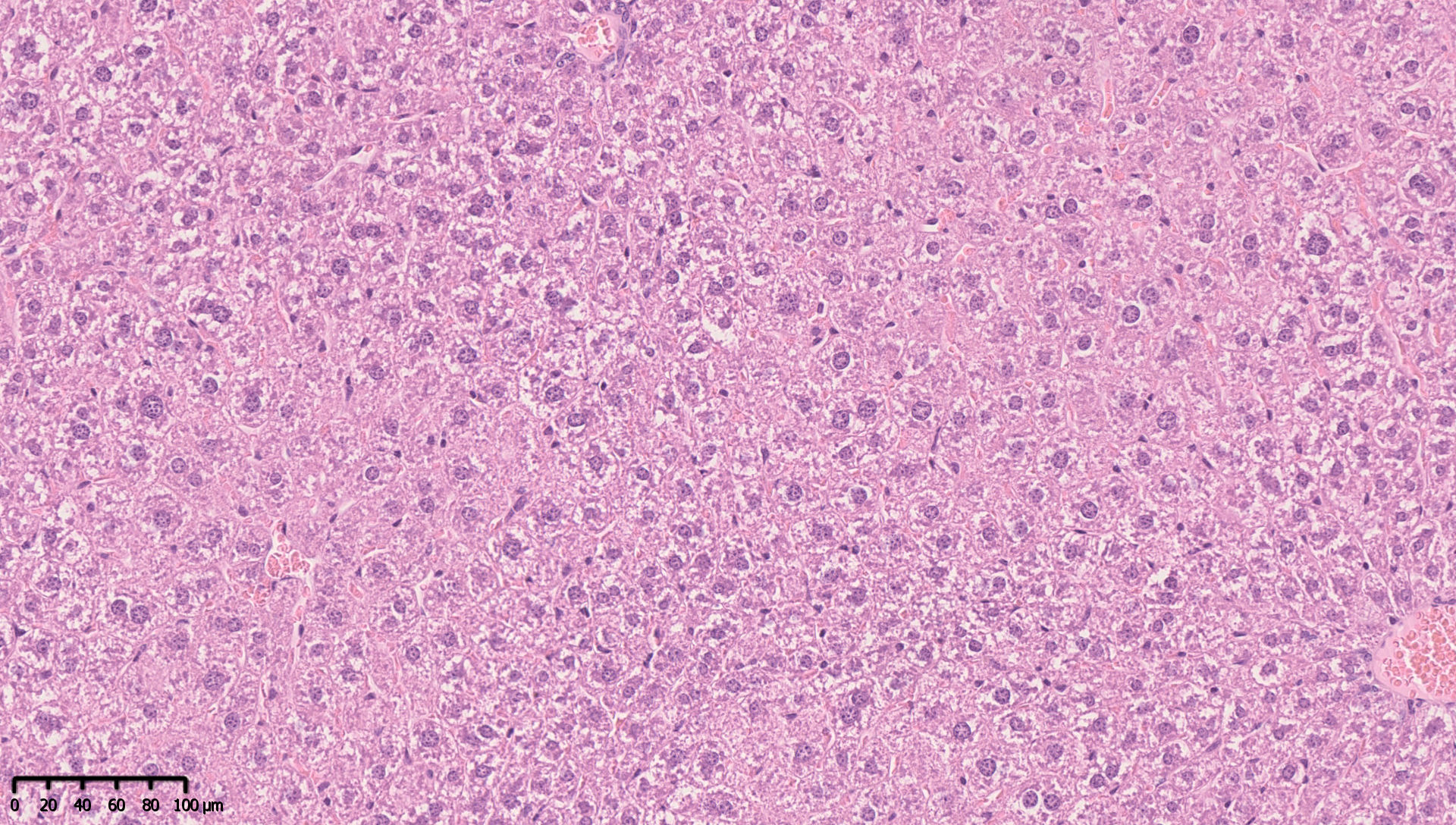

Supplement: Supplementary file 10 — Source data Fig. 4 [file 44321_2024_160_MOESM10_ESM.zip › Figure 4/4I/HE AAV-TBG+DEHP-Luteolin.jpg]

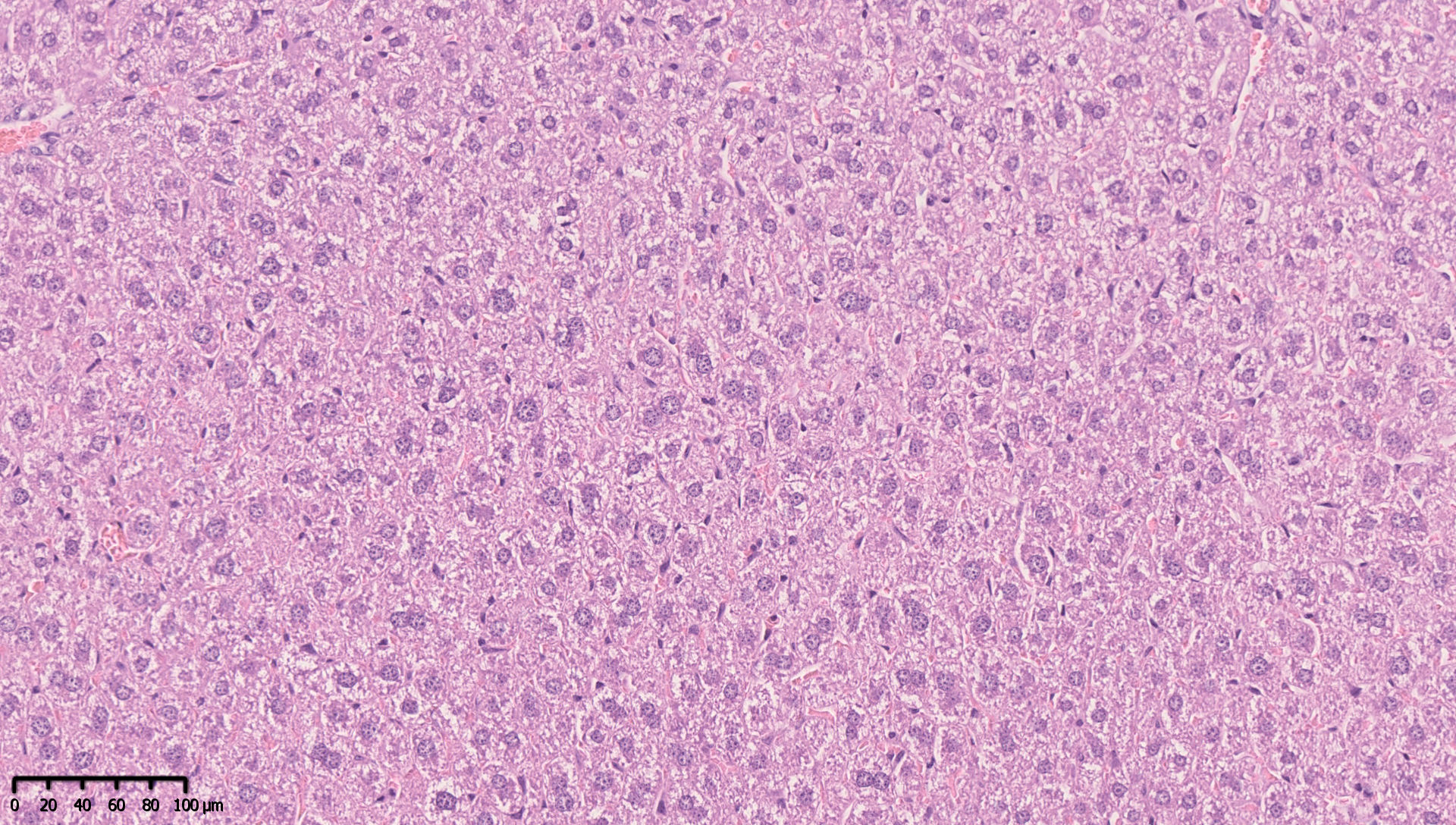

Supplement: Supplementary file 10 — Source data Fig. 4 [file 44321_2024_160_MOESM10_ESM.zip › Figure 4/4I/HE AAV-TBG+DEHP.jpg]

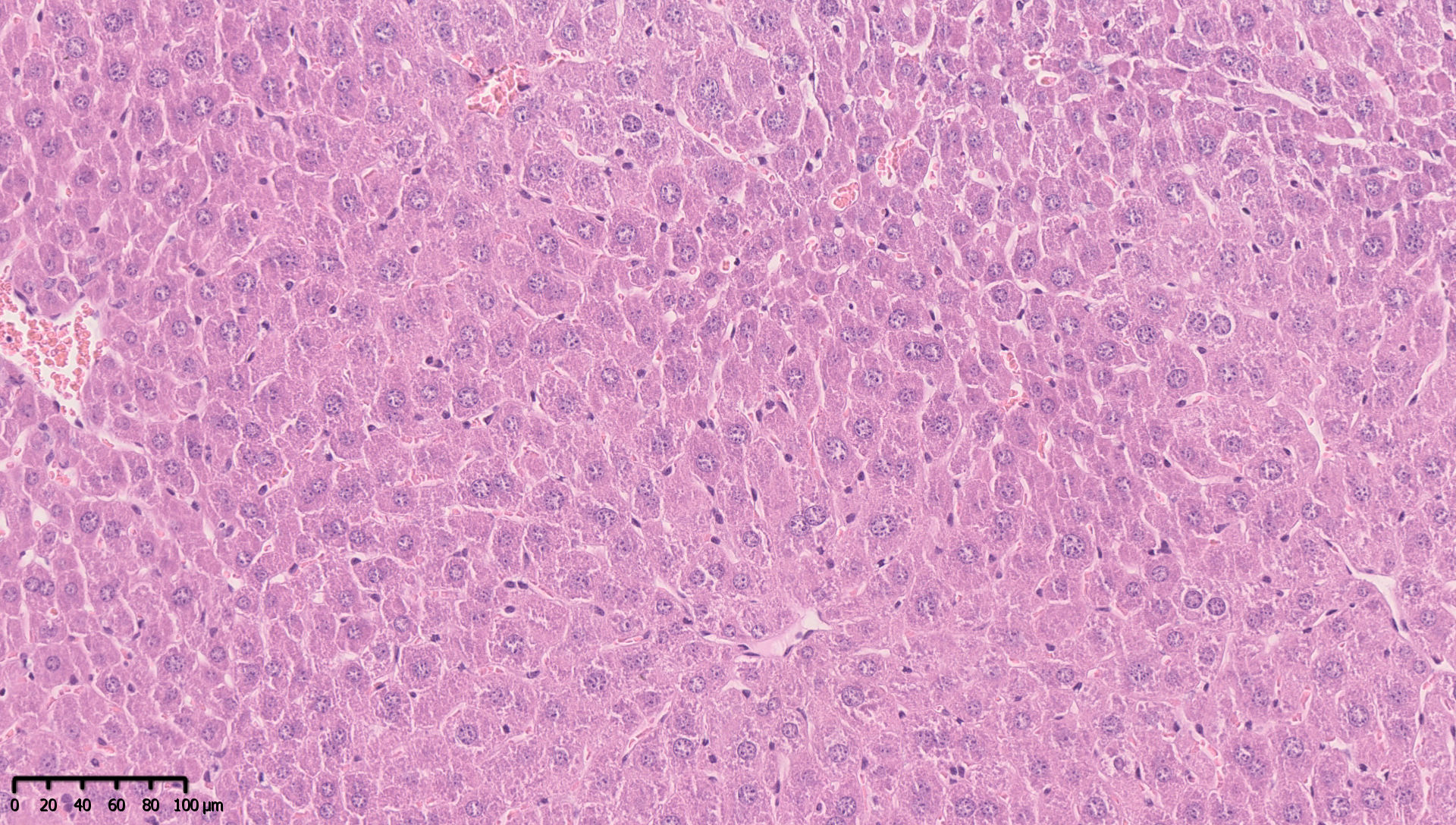

Supplement: Supplementary file 10 — Source data Fig. 4 [file 44321_2024_160_MOESM10_ESM.zip › Figure 4/4I/HE AAV-UROC1+DEHP+Luteolin.jpg]

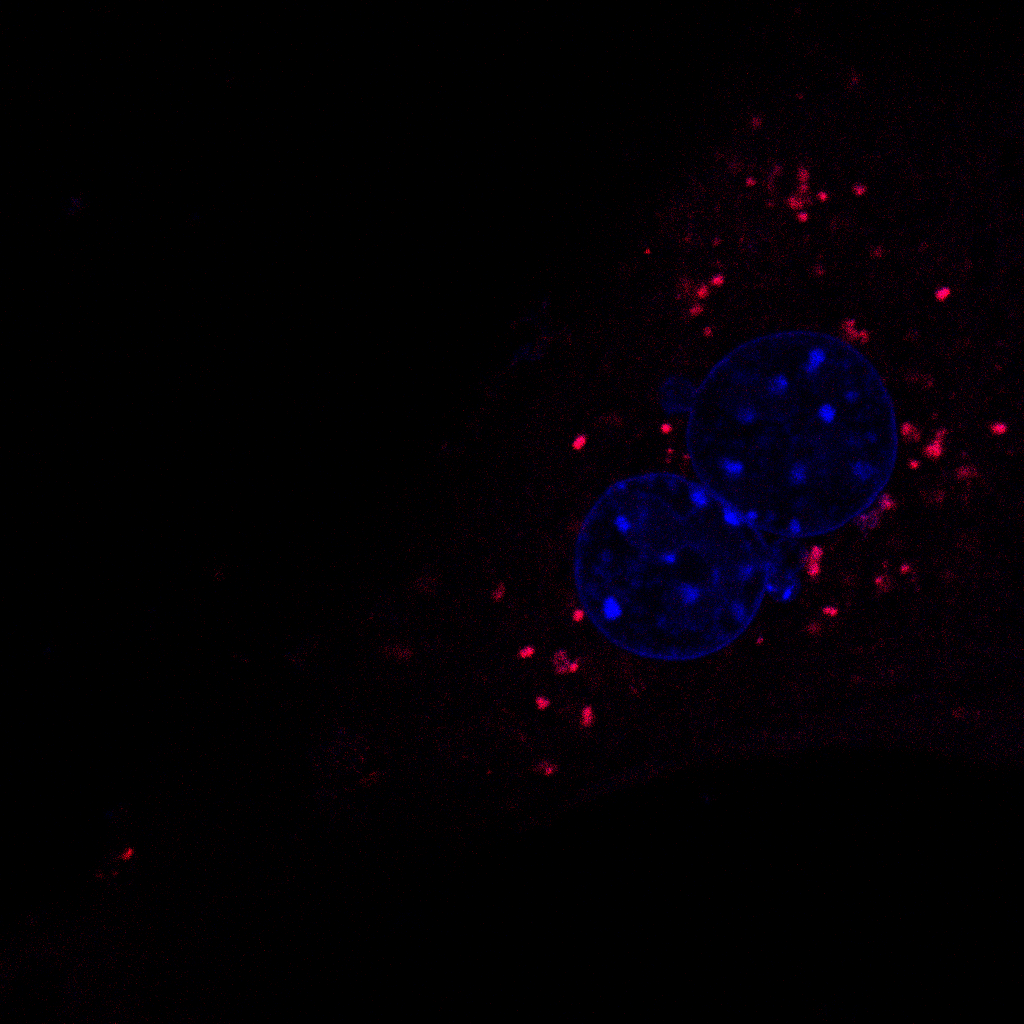

Supplement: Supplementary file 11 — Source data Fig. 5 [file 44321_2024_160_MOESM11_ESM.zip › Figure 5/5B/CTL.tif]

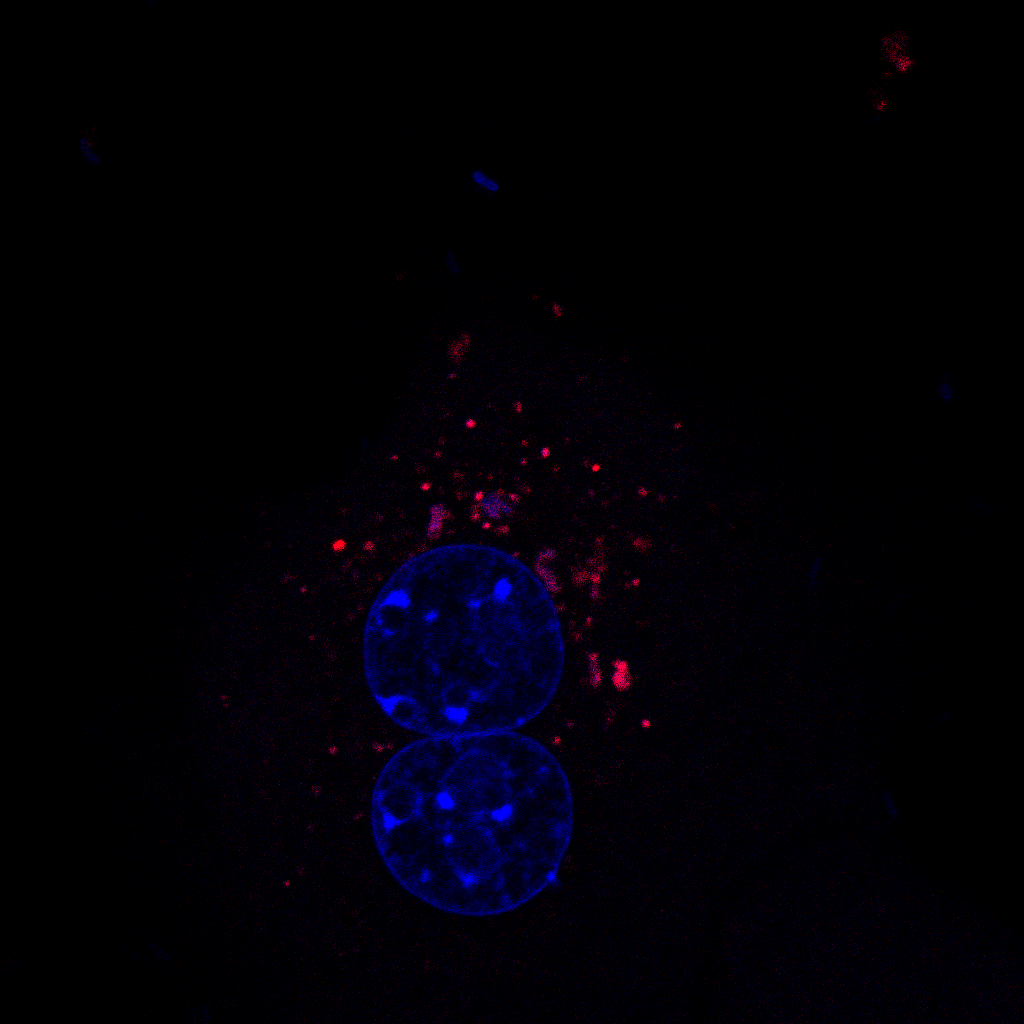

Supplement: Supplementary file 11 — Source data Fig. 5 [file 44321_2024_160_MOESM11_ESM.zip › Figure 5/5B/L-His.tif]

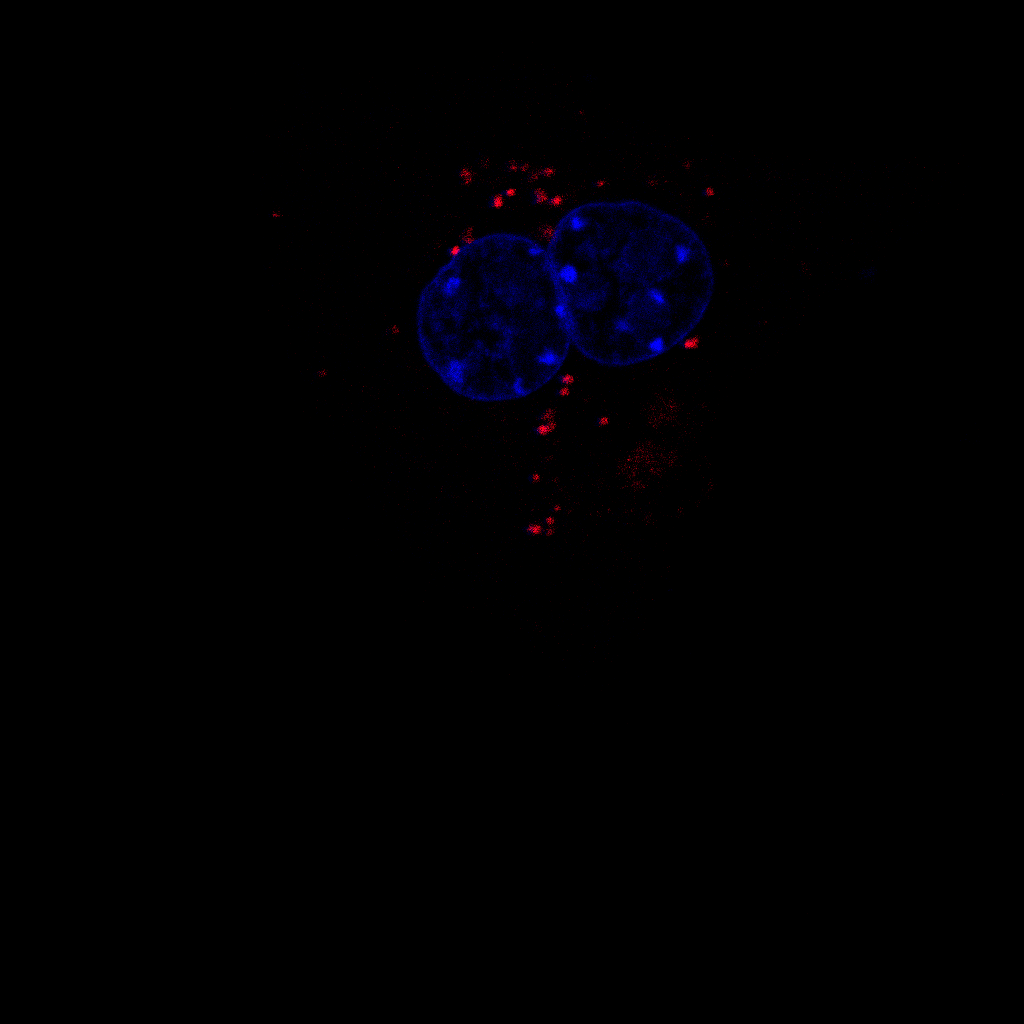

Supplement: Supplementary file 11 — Source data Fig. 5 [file 44321_2024_160_MOESM11_ESM.zip › Figure 5/5B/Luteolin.tif]

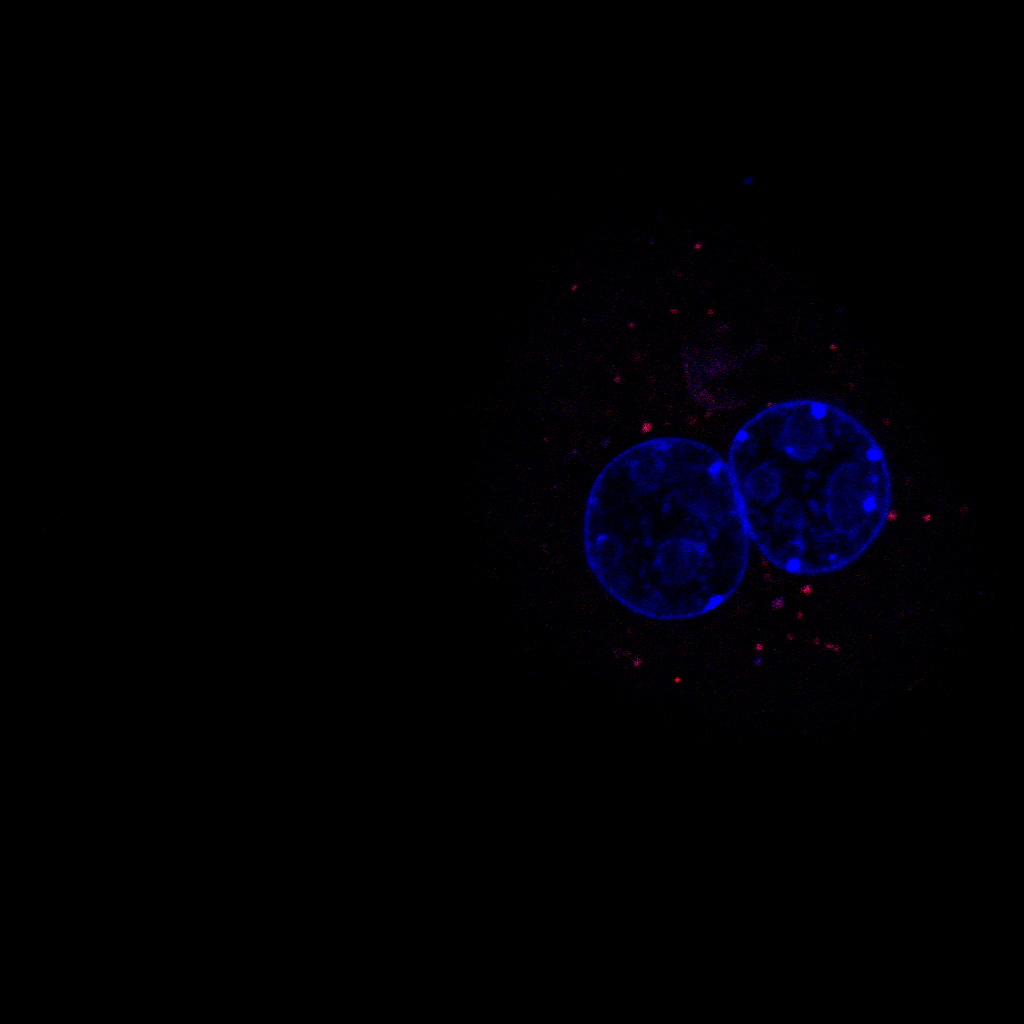

Supplement: Supplementary file 11 — Source data Fig. 5 [file 44321_2024_160_MOESM11_ESM.zip › Figure 5/5B/trans-UCA.tif]

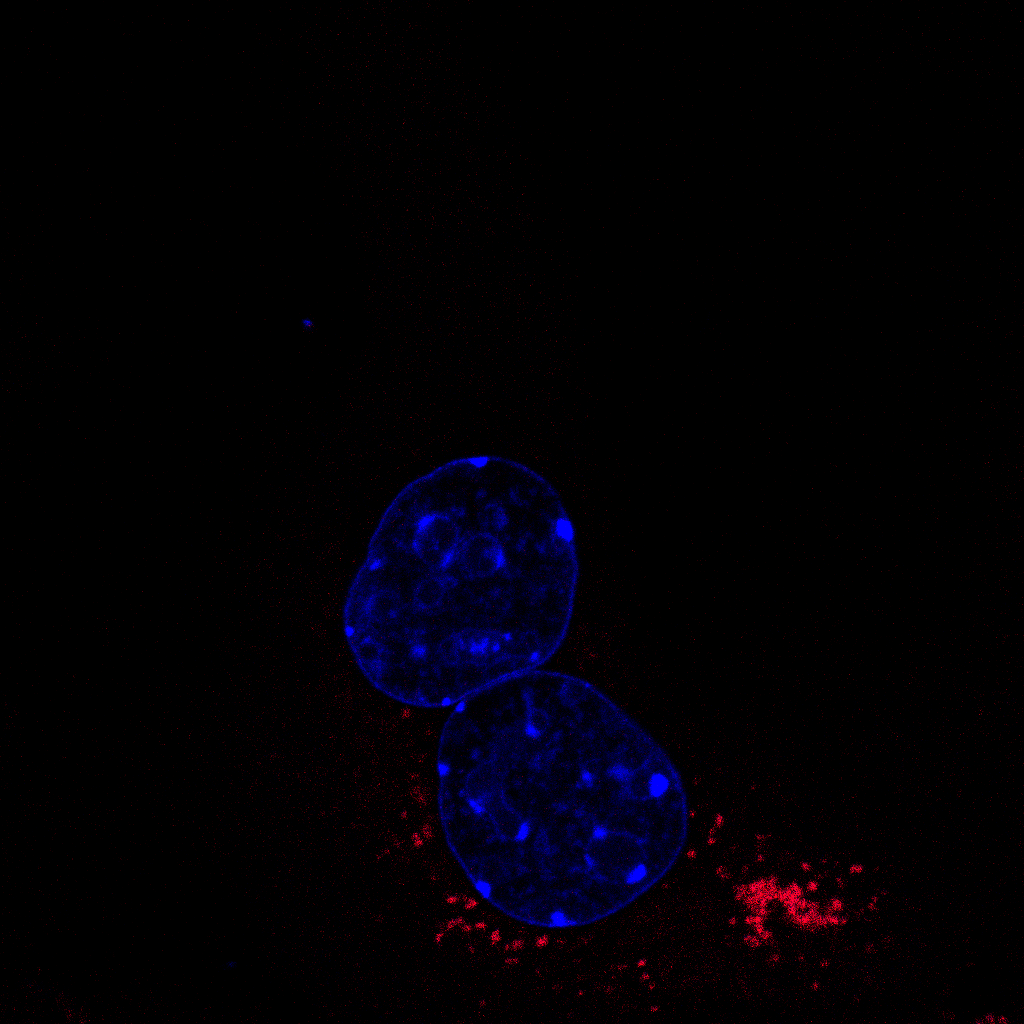

Supplement: Supplementary file 11 — Source data Fig. 5 [file 44321_2024_160_MOESM11_ESM.zip › Figure 5/5D/trans-UCA 0 mM.tif]

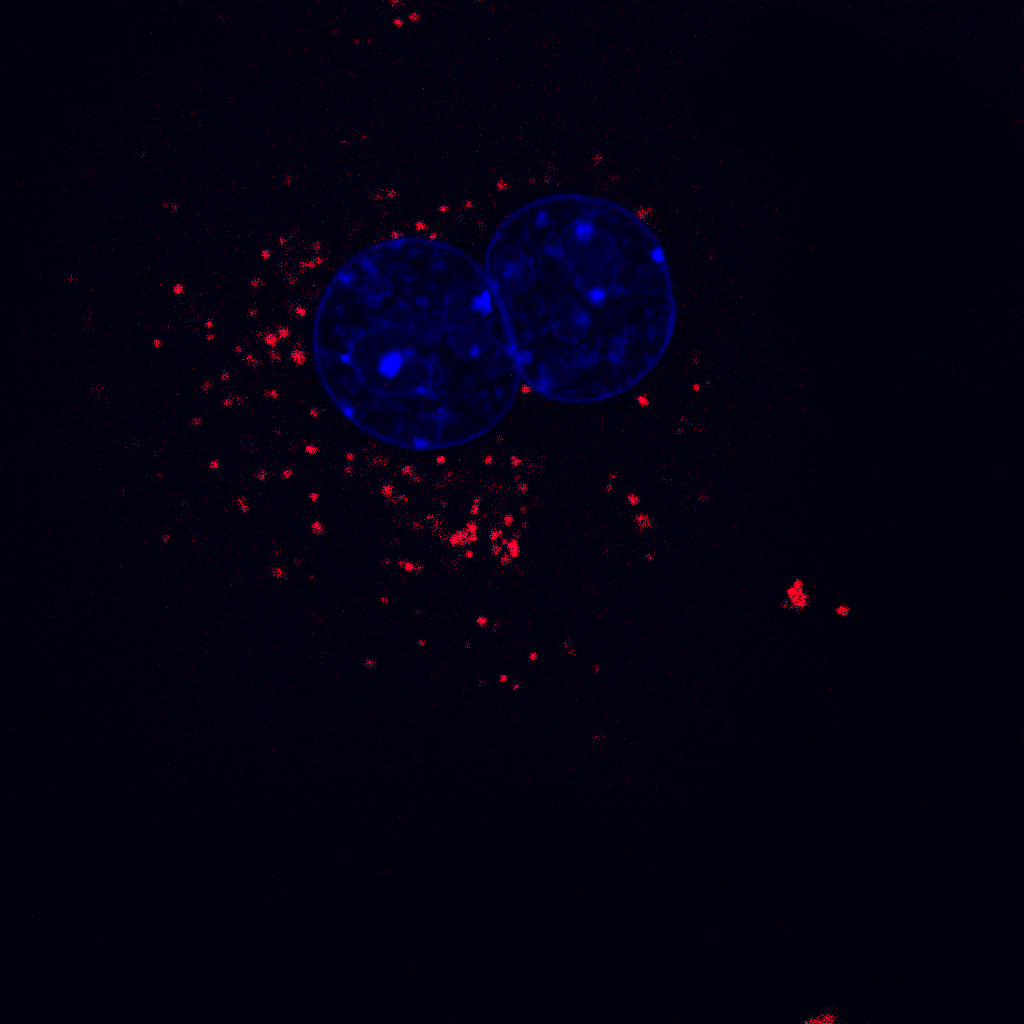

Supplement: Supplementary file 11 — Source data Fig. 5 [file 44321_2024_160_MOESM11_ESM.zip › Figure 5/5D/trans-UCA 0h.tif]

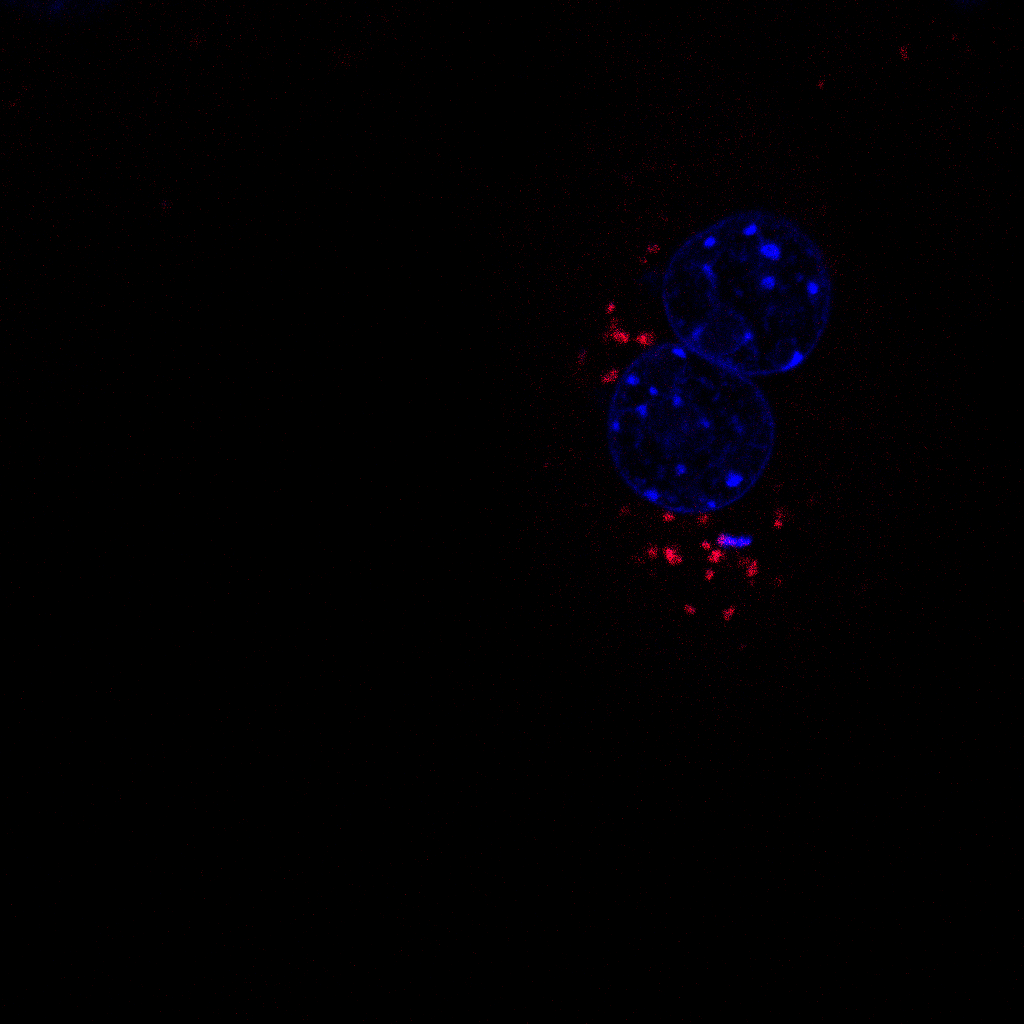

Supplement: Supplementary file 11 — Source data Fig. 5 [file 44321_2024_160_MOESM11_ESM.zip › Figure 5/5D/trans-UCA 1 mM.tif]

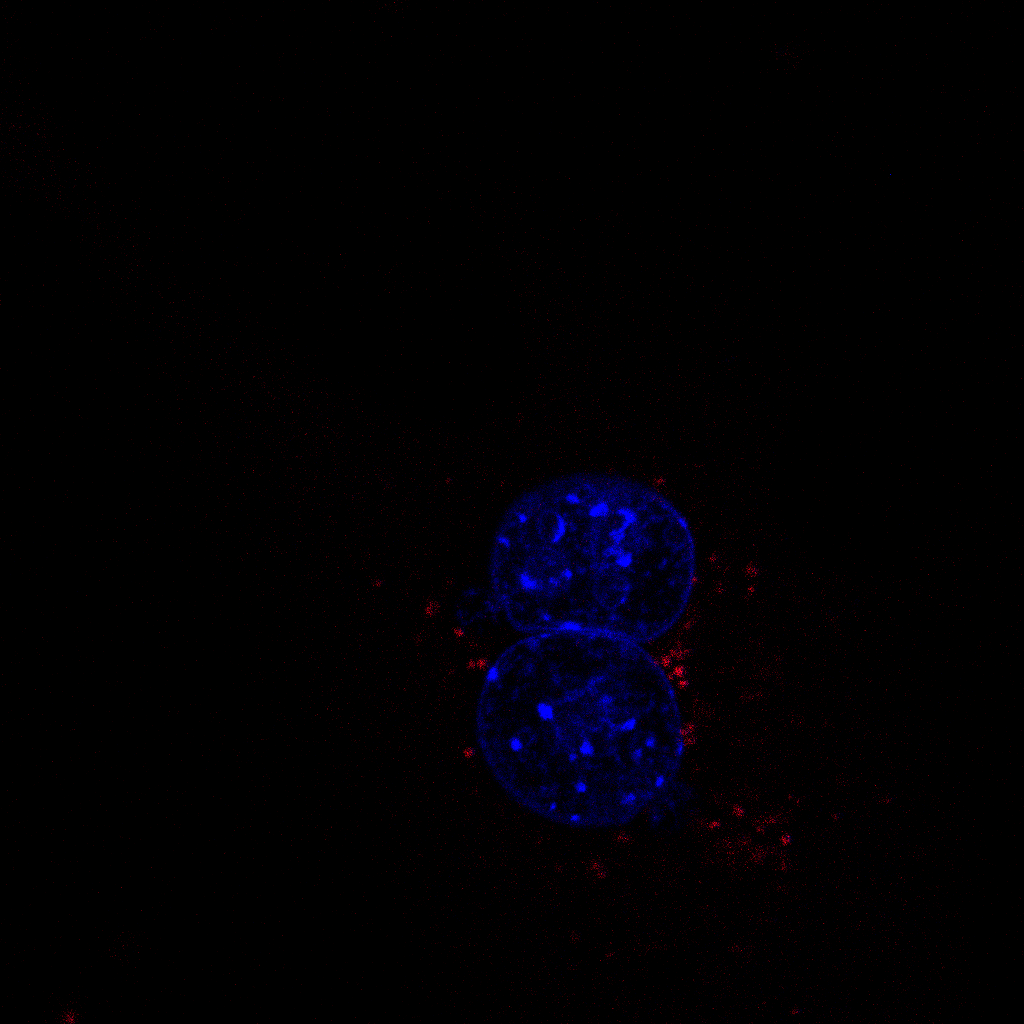

Supplement: Supplementary file 11 — Source data Fig. 5 [file 44321_2024_160_MOESM11_ESM.zip › Figure 5/5D/trans-UCA 10 mM.tif]

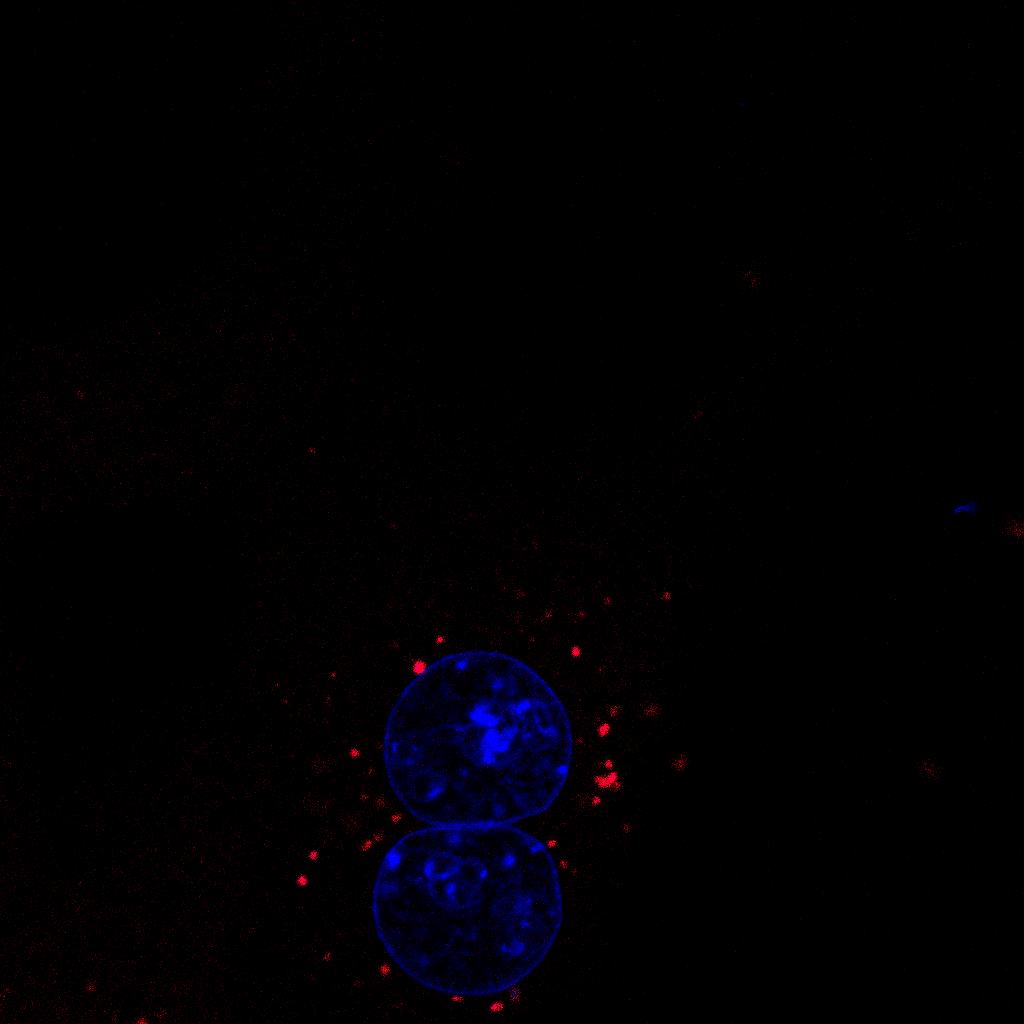

Supplement: Supplementary file 11 — Source data Fig. 5 [file 44321_2024_160_MOESM11_ESM.zip › Figure 5/5D/trans-UCA 12h.tif]

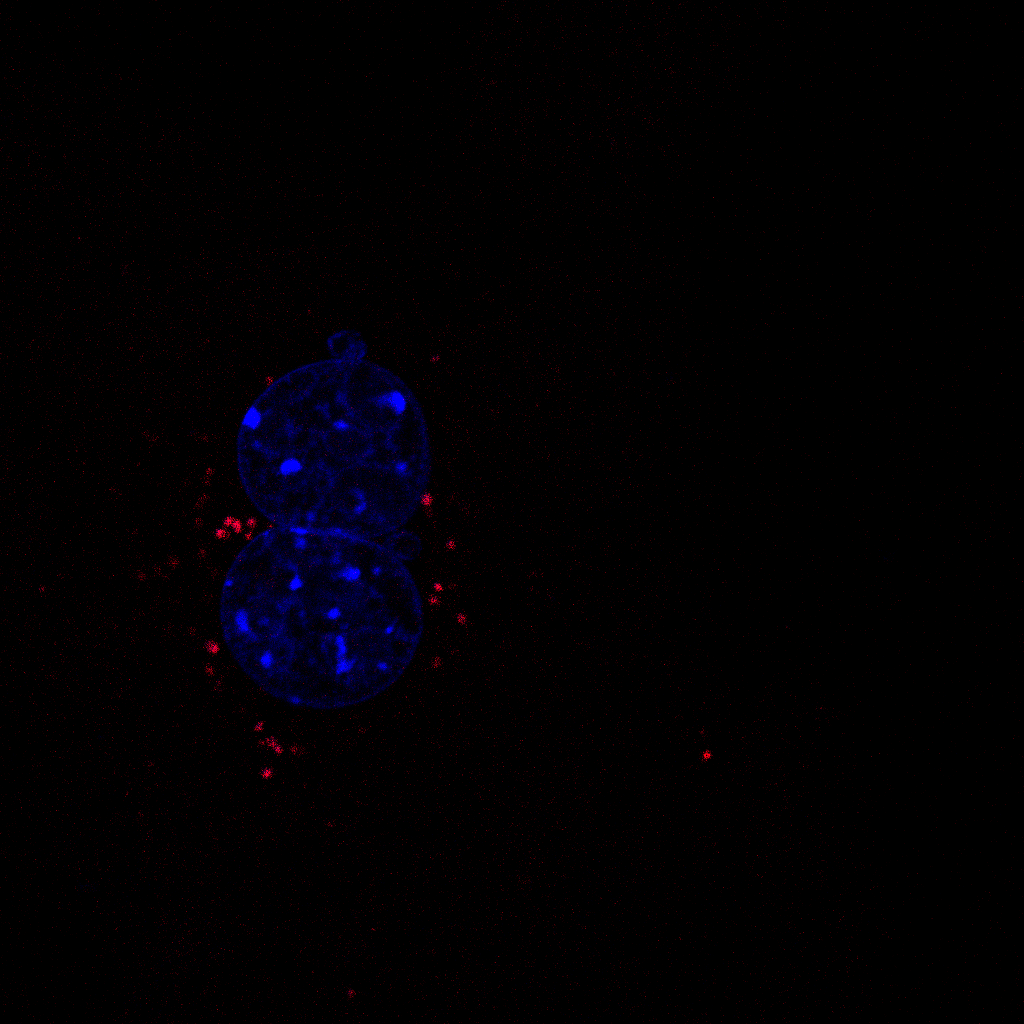

Supplement: Supplementary file 11 — Source data Fig. 5 [file 44321_2024_160_MOESM11_ESM.zip › Figure 5/5D/trans-UCA 24h.tif]

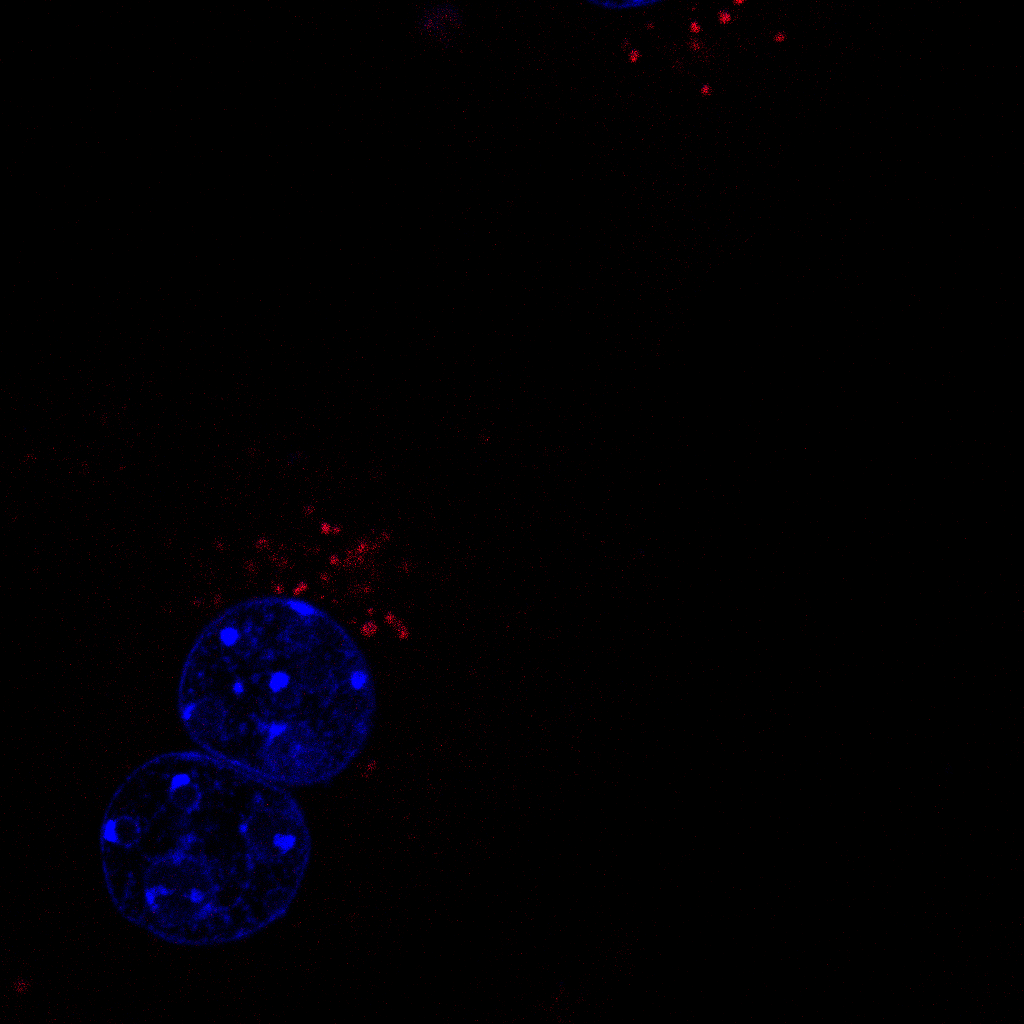

Supplement: Supplementary file 11 — Source data Fig. 5 [file 44321_2024_160_MOESM11_ESM.zip › Figure 5/5D/trans-UCA 5 mM.tif]

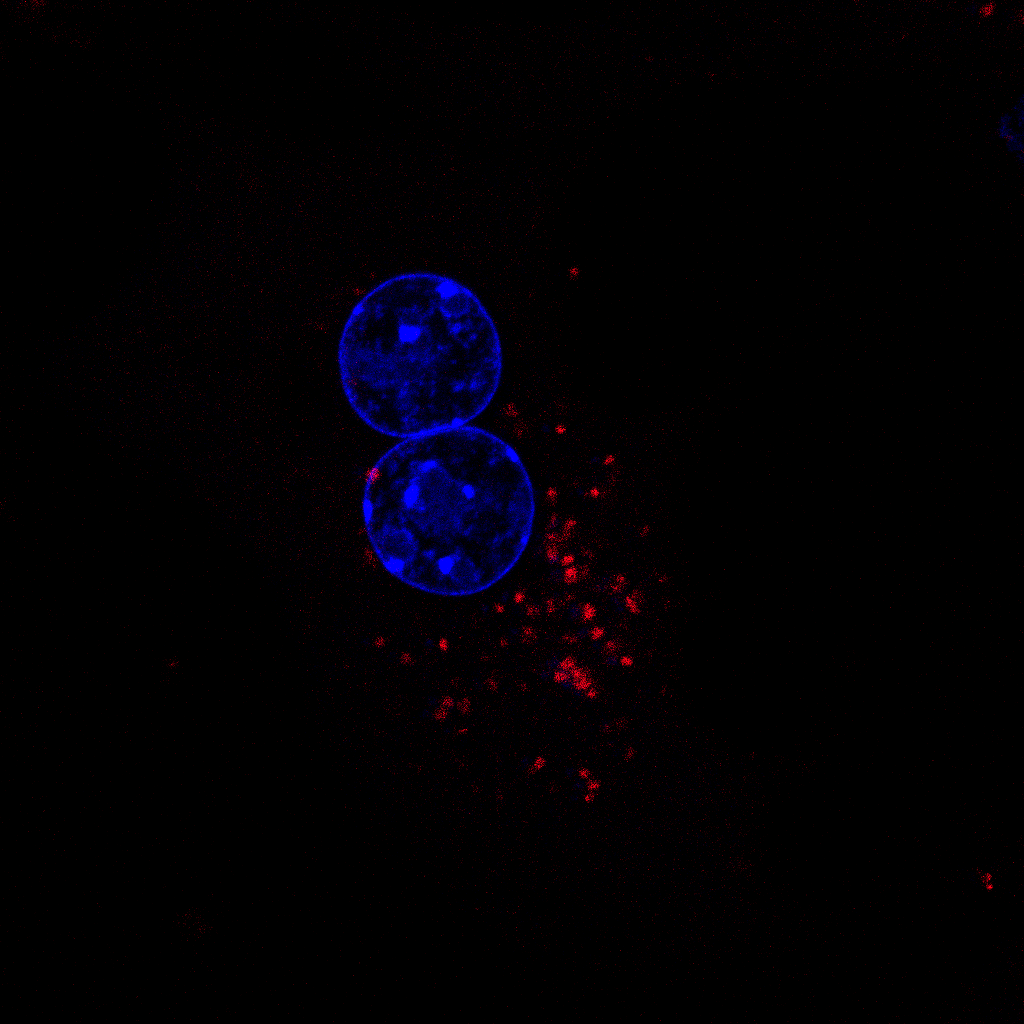

Supplement: Supplementary file 11 — Source data Fig. 5 [file 44321_2024_160_MOESM11_ESM.zip › Figure 5/5D/trans-UCA 6h.tif]

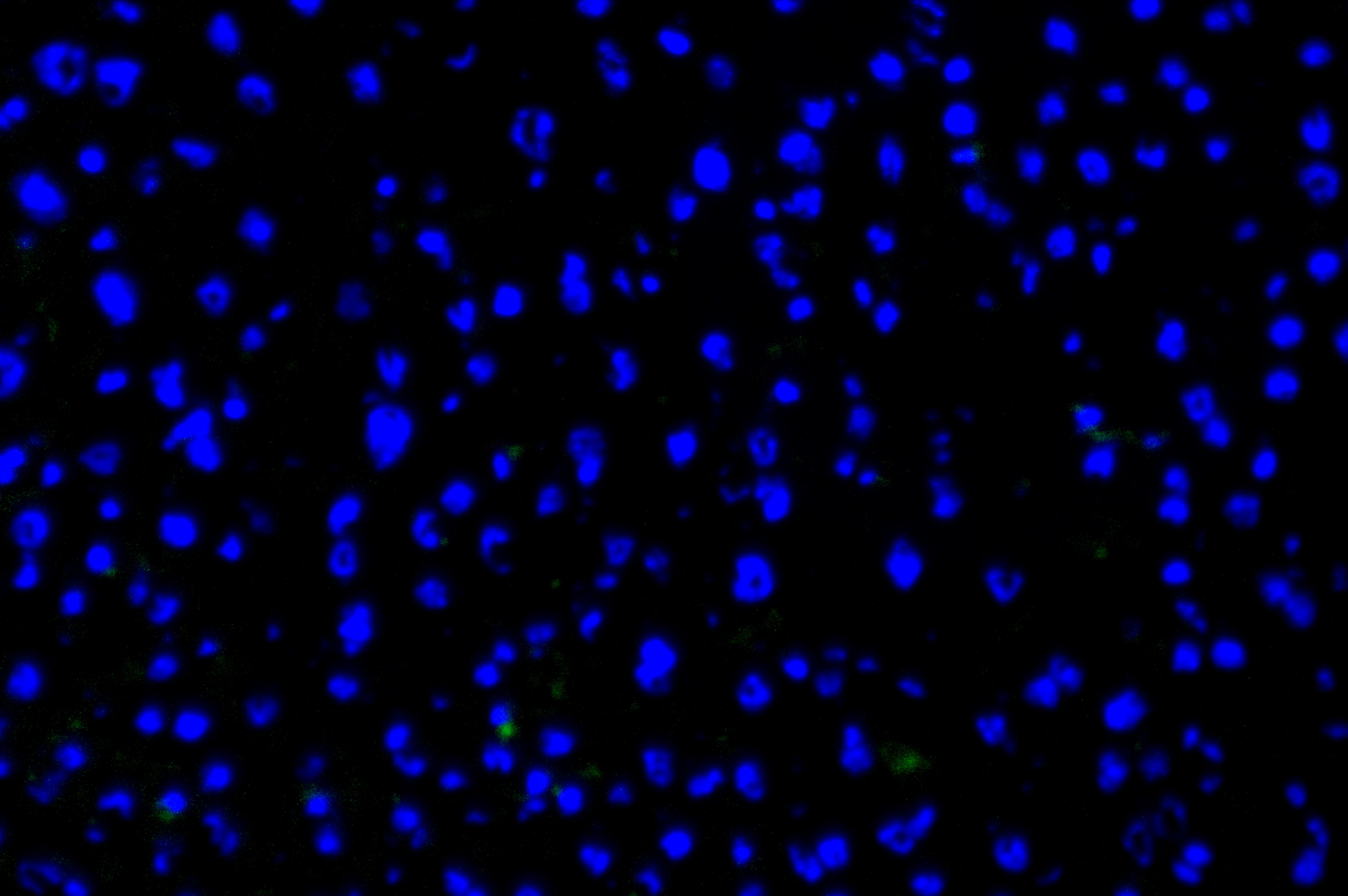

Supplement: Supplementary file 11 — Source data Fig. 5 [file 44321_2024_160_MOESM11_ESM.zip › Figure 5/5H/F480 DEHP+trans-UCA.tif]

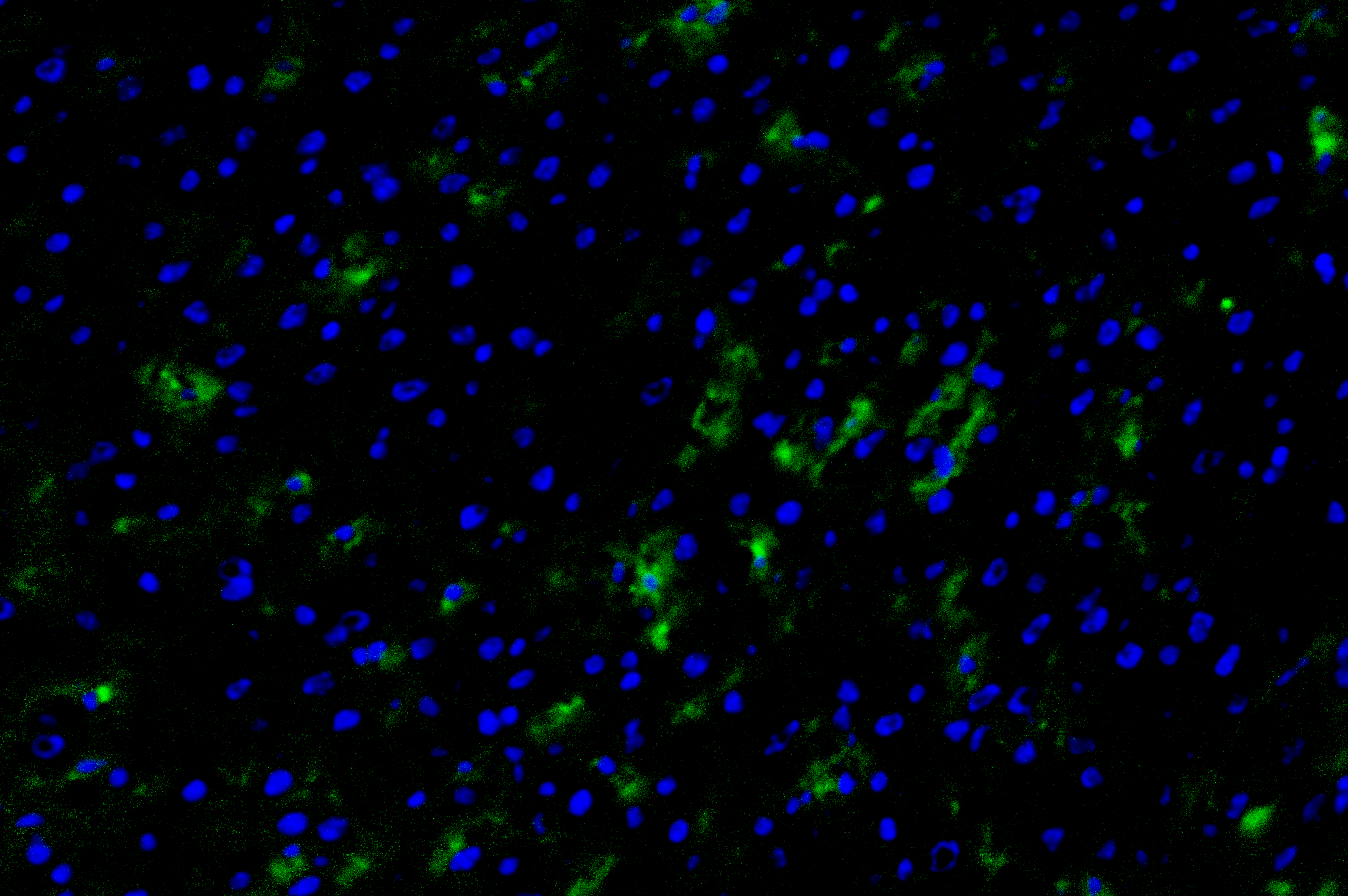

Supplement: Supplementary file 11 — Source data Fig. 5 [file 44321_2024_160_MOESM11_ESM.zip › Figure 5/5H/F480 DEHP.tif]

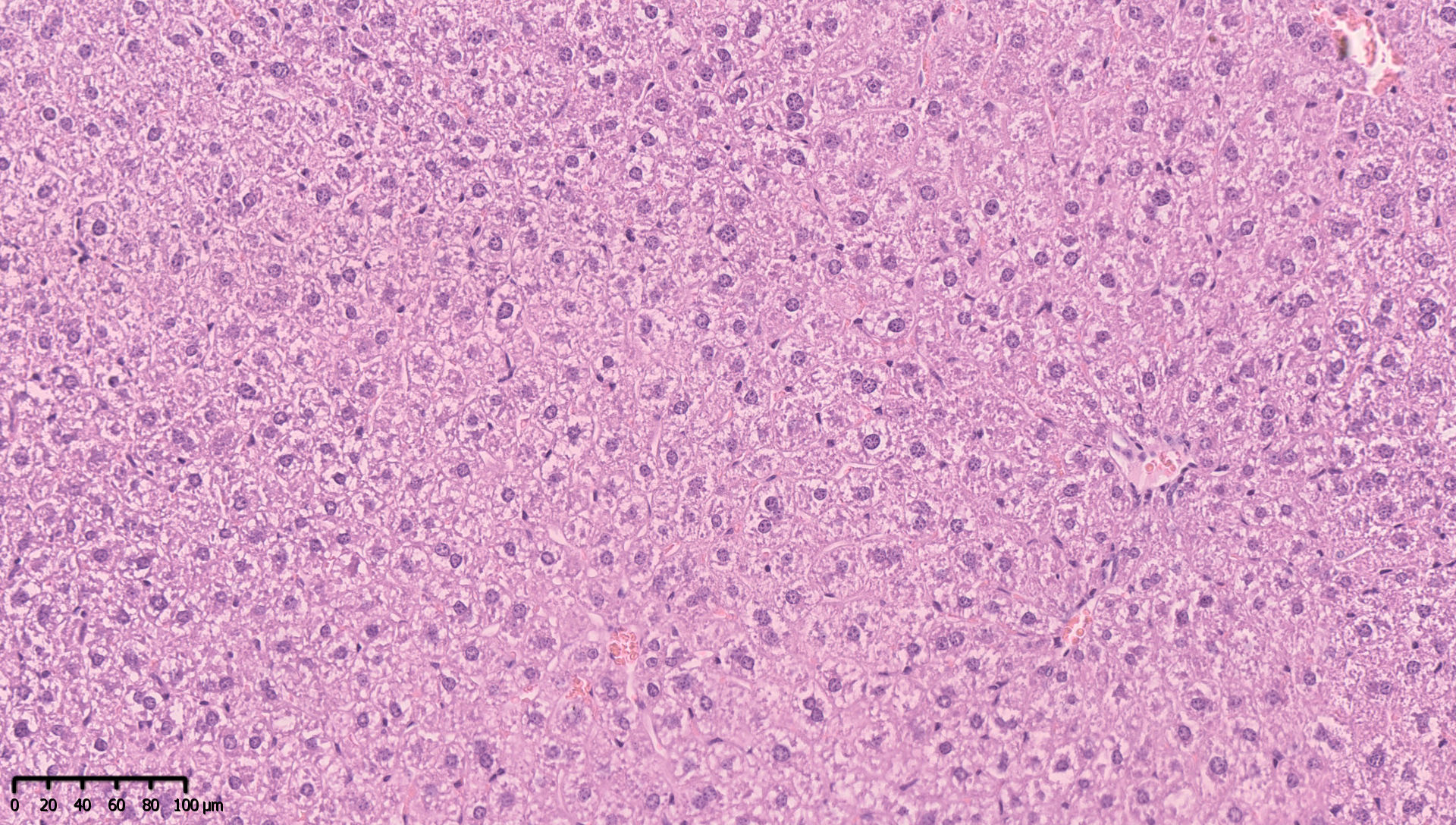

Supplement: Supplementary file 11 — Source data Fig. 5 [file 44321_2024_160_MOESM11_ESM.zip › Figure 5/5H/HE DEHP+trans-UCA.jpg]

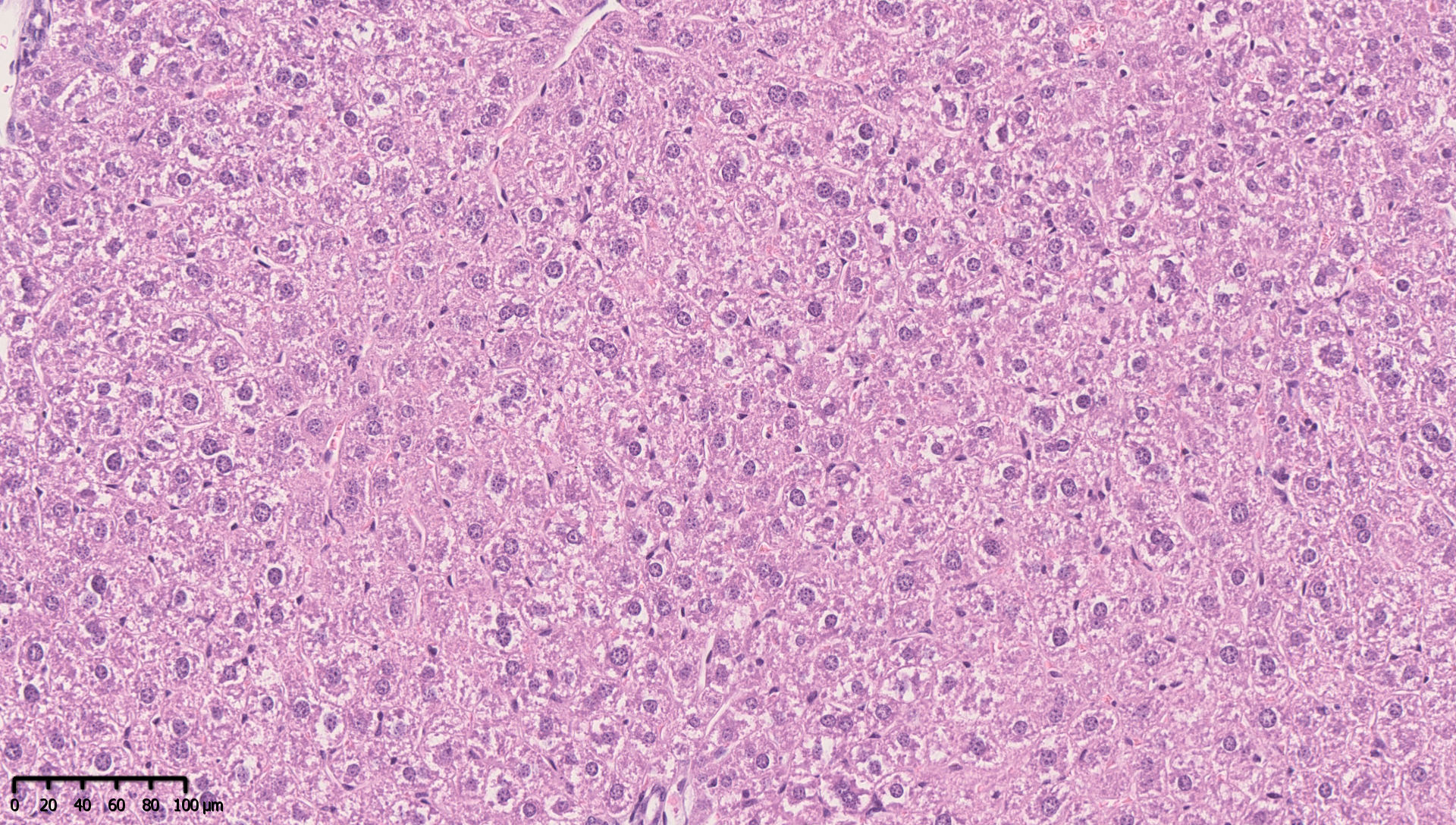

Supplement: Supplementary file 11 — Source data Fig. 5 [file 44321_2024_160_MOESM11_ESM.zip › Figure 5/5H/HE DEHP.jpg]

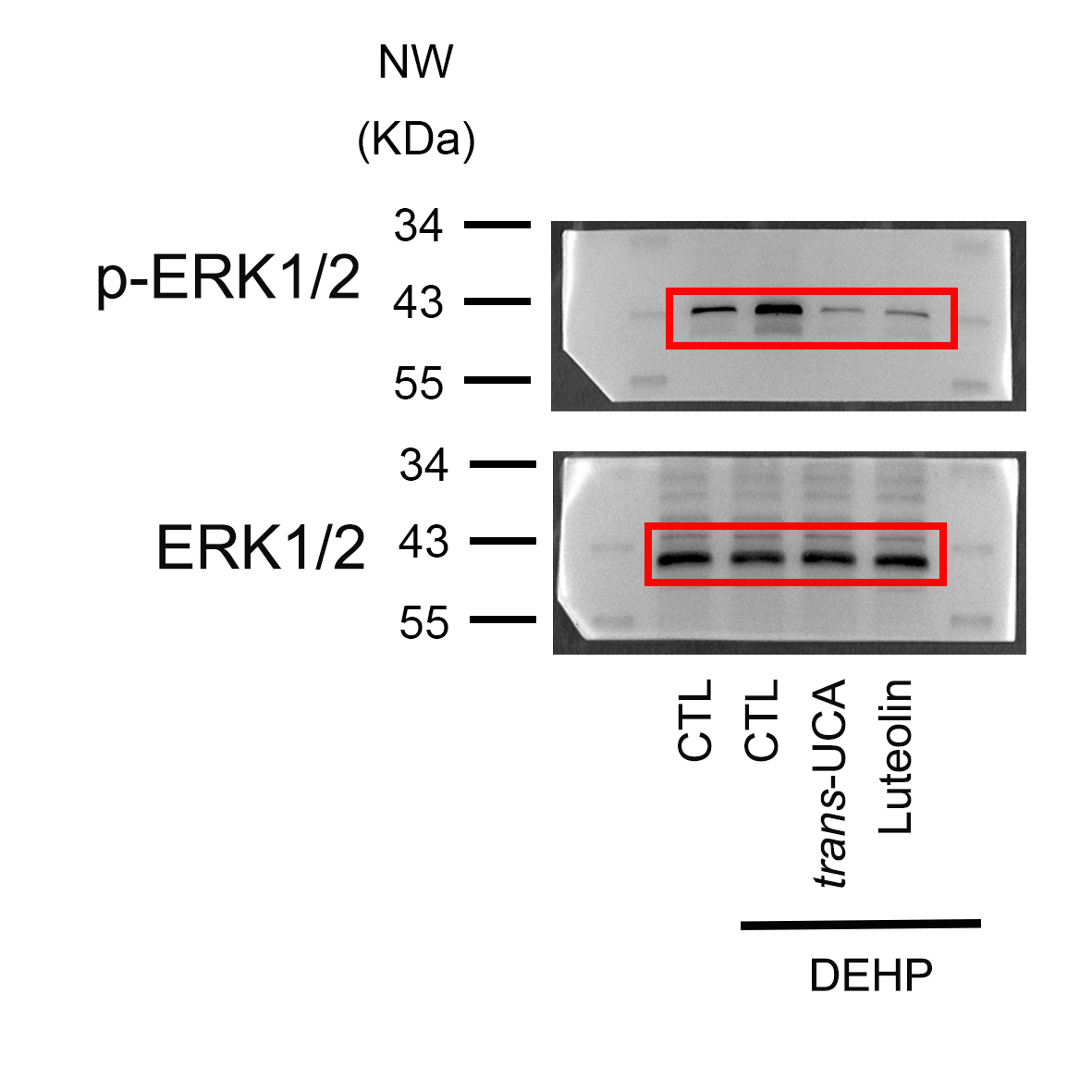

Supplement: Supplementary file 12 — Source data Fig. 6 [file 44321_2024_160_MOESM12_ESM.zip › Figure 6/6D/6D.tif]

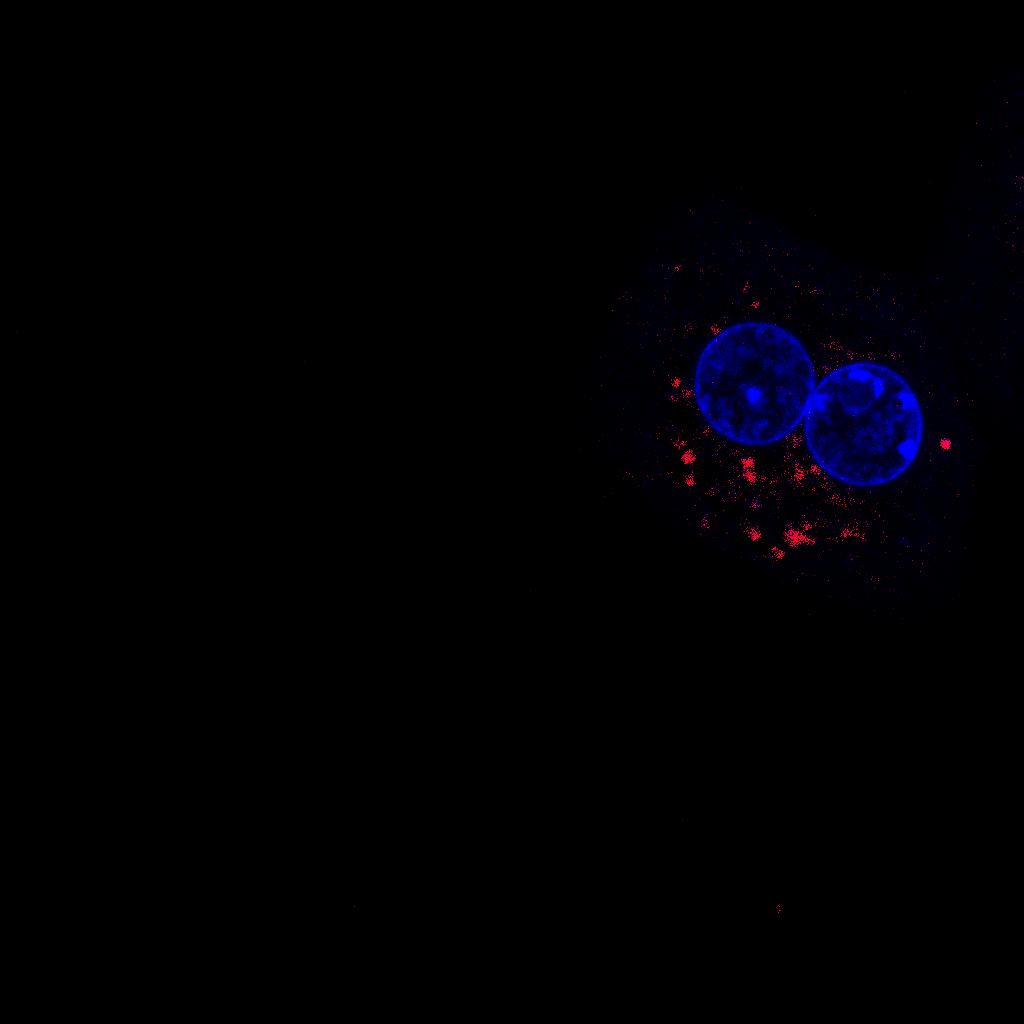

Supplement: Supplementary file 12 — Source data Fig. 6 [file 44321_2024_160_MOESM12_ESM.zip › Figure 6/6E/CTL.tif]

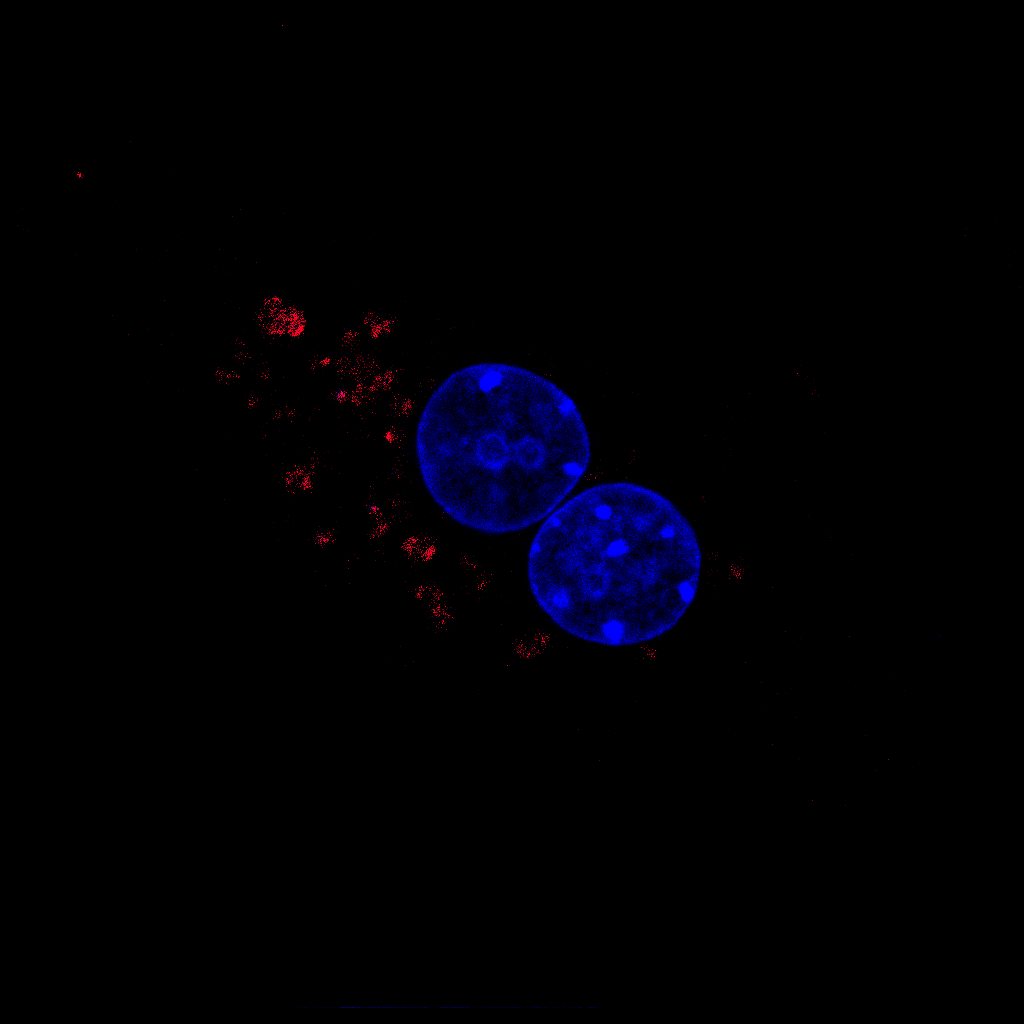

Supplement: Supplementary file 12 — Source data Fig. 6 [file 44321_2024_160_MOESM12_ESM.zip › Figure 6/6E/Luteolin+Baf A1.tif]

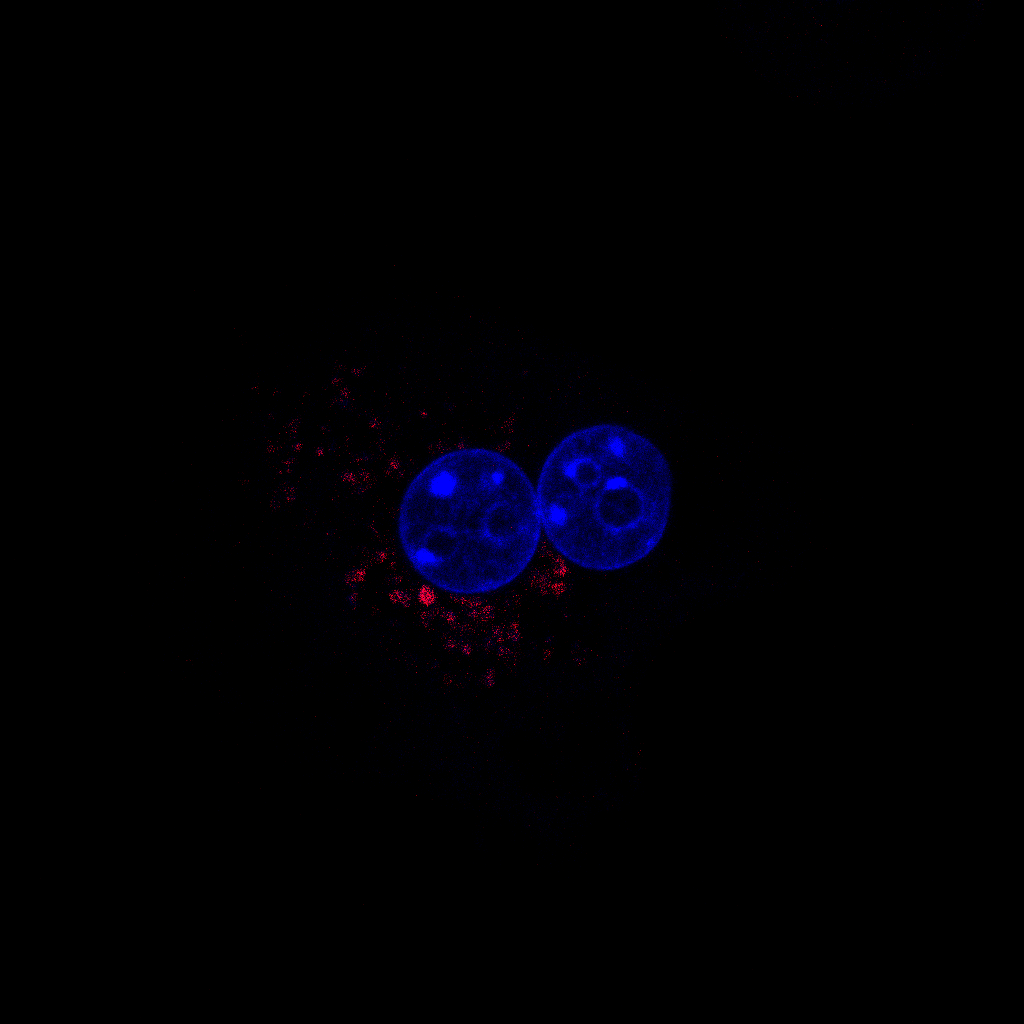

Supplement: Supplementary file 12 — Source data Fig. 6 [file 44321_2024_160_MOESM12_ESM.zip › Figure 6/6E/Luteolin.tif]

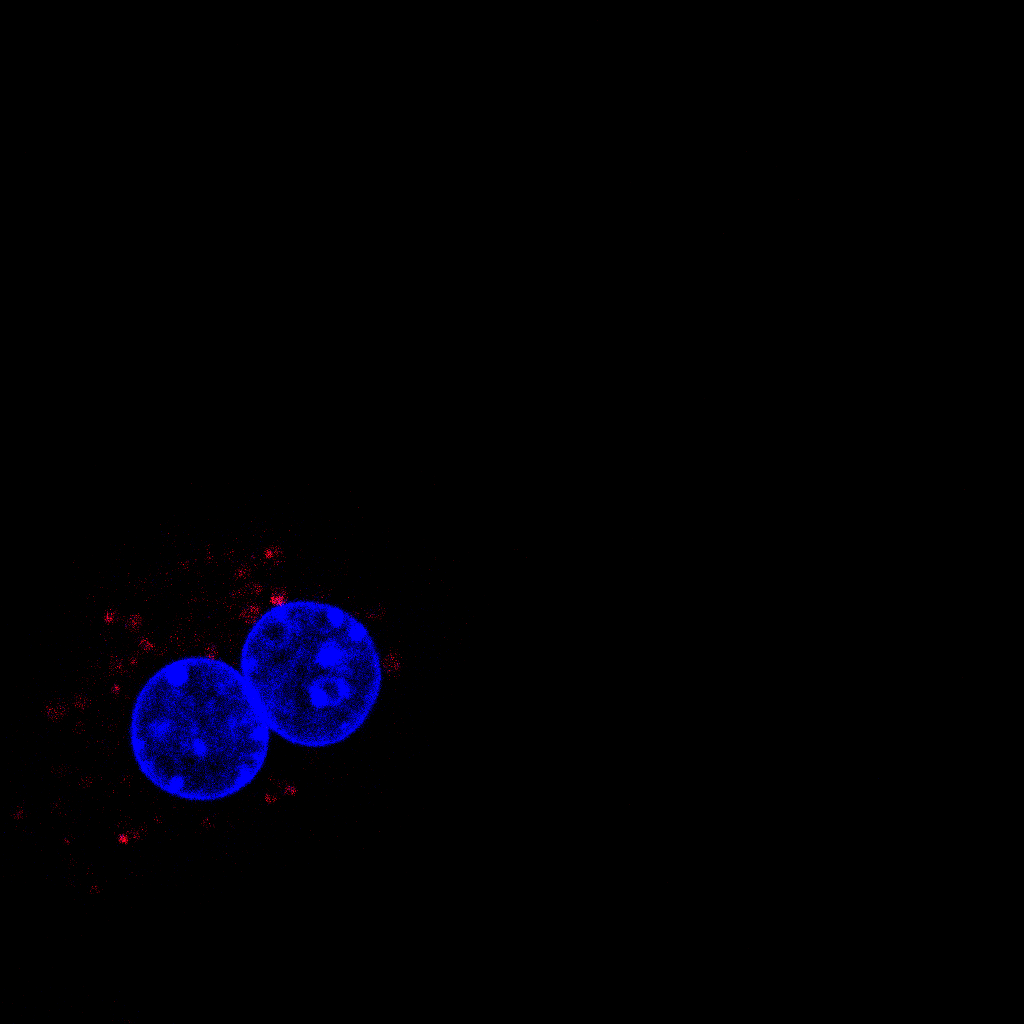

Supplement: Supplementary file 12 — Source data Fig. 6 [file 44321_2024_160_MOESM12_ESM.zip › Figure 6/6E/trans-UCA+Baf A1.tif]

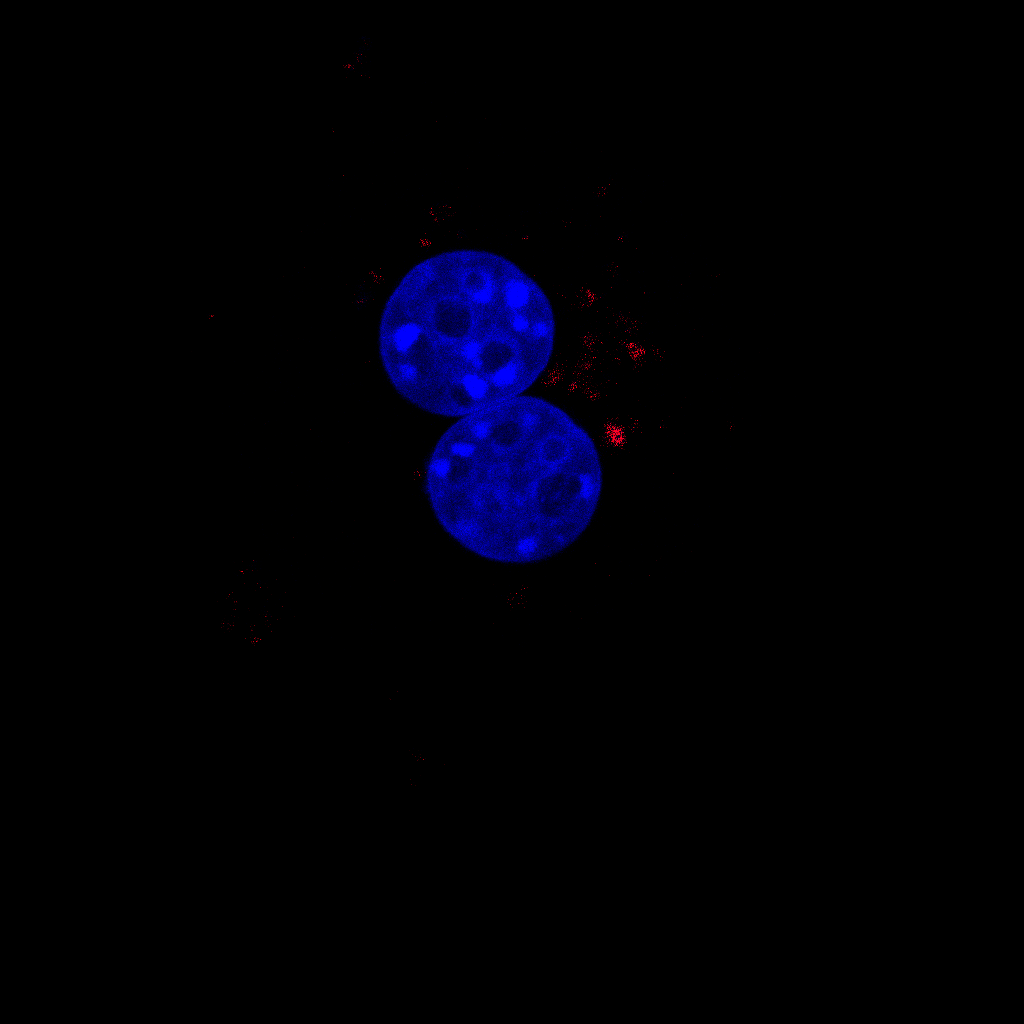

Supplement: Supplementary file 12 — Source data Fig. 6 [file 44321_2024_160_MOESM12_ESM.zip › Figure 6/6E/trans-UCA.tif]
