# Supplementary material for: Luteolin detoxifies DEHP and prevents liver injury by degrading Uroc1 protein in mice
Source: EMBO Mol Med. 2024 Oct 29;16(11):2699–724. doi: 10.1038/s44321-024-00160-9 (PMC11555401; doi:10.1038/s44321-024-00160-9)
Supplement: Supplementary file 13 — Expanded View Figures [file 44321_2024_160_MOESM13_ESM.pdf]

Expanded View Figures

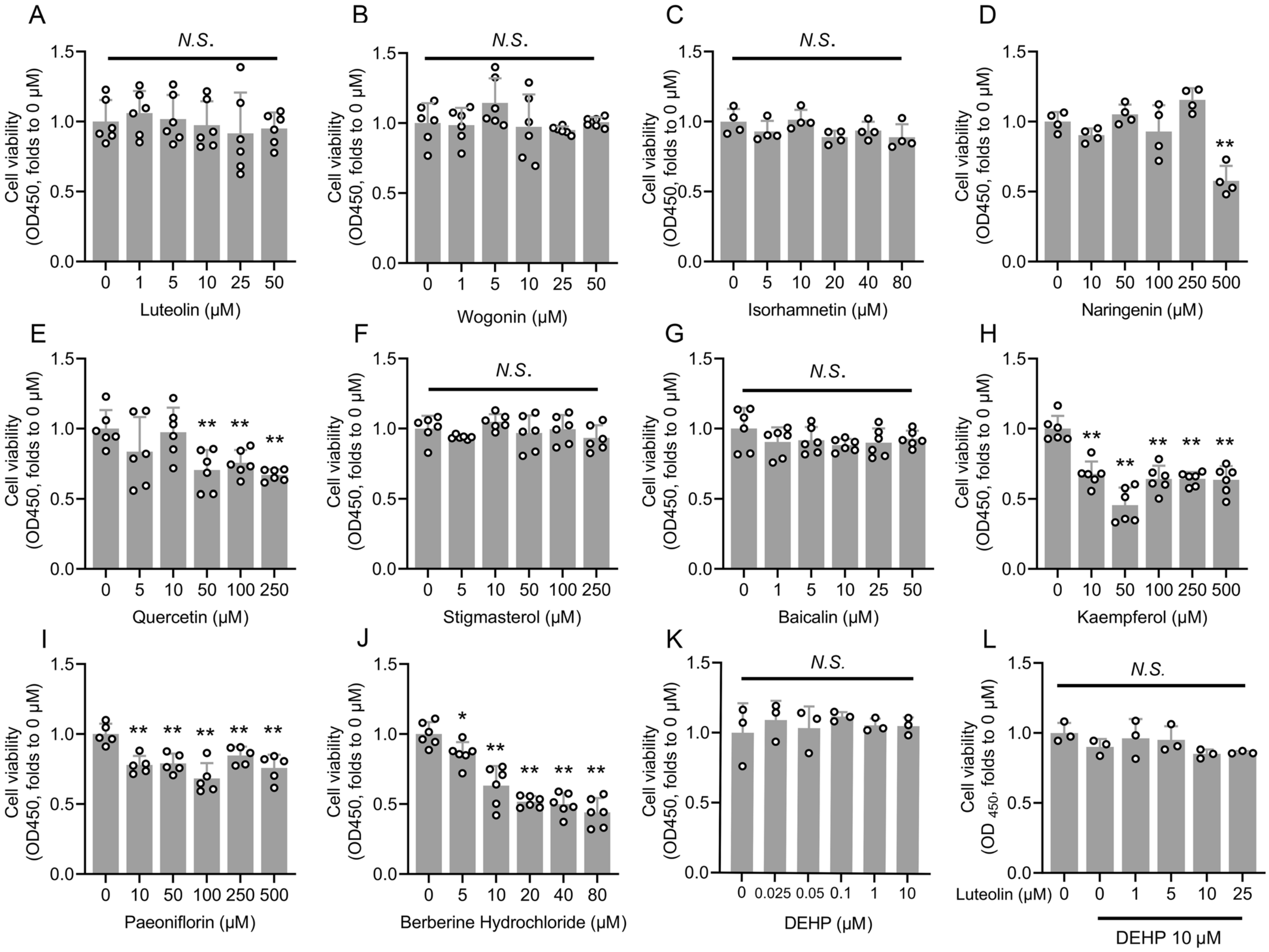

Figure EV1. Cell toxicity analysis of candidate drugs in moue PHs.

(**A**) Cell viability analysis of mouse PHs treated with indicated doses of luteolin. $n = 6$. (**B**) Cell viability analysis of mouse PHs treated with indicated doses of wogonin. $n = 6$. (**C**) Cell viability analysis of mouse PHs treated with indicated doses of isorhamnetin. $n = 4$. (**D**) Cell viability analysis of mouse PHs treated with indicated doses of naringenin. $n = 4$. (**E**) Cell viability analysis of mouse PHs treated with indicated doses of quercetin. $n = 6$. (**F**) Cell viability analysis of mouse PHs treated with indicated doses of stigmasterol. $n = 6$. (**G**) Cell viability analysis of mouse PHs treated with indicated doses of baicalin. $n = 6$. (**H**) Cell viability analysis of mouse PHs treated with indicated doses of kaempferol. $n = 6$. (**I**) Cell viability analysis of mouse PHs treated with indicated doses of paeoniflorin. $n = 5$. (**J**) Cell viability analysis of mouse PHs treated with indicated doses of berberine hydrochloride. $n = 6$. (**K**) Cell viability analysis of mouse PHs treated with indicated doses of DEHP. $n = 3$. (**L**) Cell viability analysis of mouse PHs exposed to 10 μM DEHP and then incubated with indicated doses of luteolin for another 24 h. $n = 3$. *N.S.*, no significance. **$P < 0.01$ *vs*. 0 μM group. All the data were represented as the mean ± SD. The paired Student's *t*-test was employed to compare between two groups. Exact *P* values are listed in Appendix Table S11.

© The Author(s)

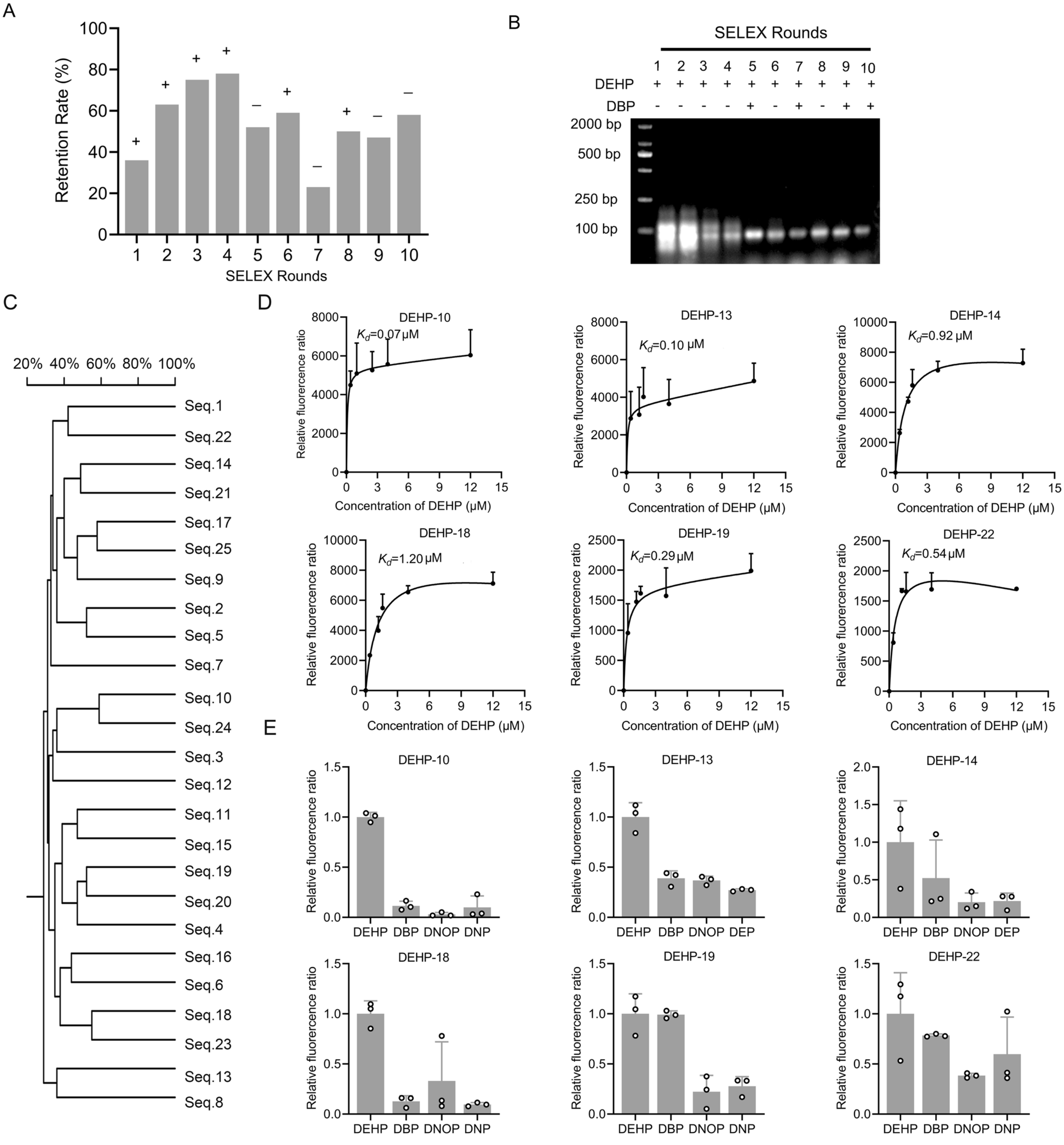

Figure EV2. Screening of DNA-aptamer targeting the DEHP.

(**A**) Retention rate of ssDNA sequences collected during the selection of DEHP aptamers using Capture-SELEX technology. (**B**) Electrophoretic verification of the ssDNA library from each screening round. (**C**) Phylogenetic tree analysis of candidate aptamers targeting DEHP. (**D**) Graphene oxide fluorescence assay for the affinity of candidate aptamers targeting DEHP. Data are fitted to a Michaelis-Menten model (curve) to calculate the dissociation constants. (**E**) Binding specificity of candidate aptamers to DEHP. $n = 3$. 'n' represents technical replicates. All the data were represented as the mean ± SD.

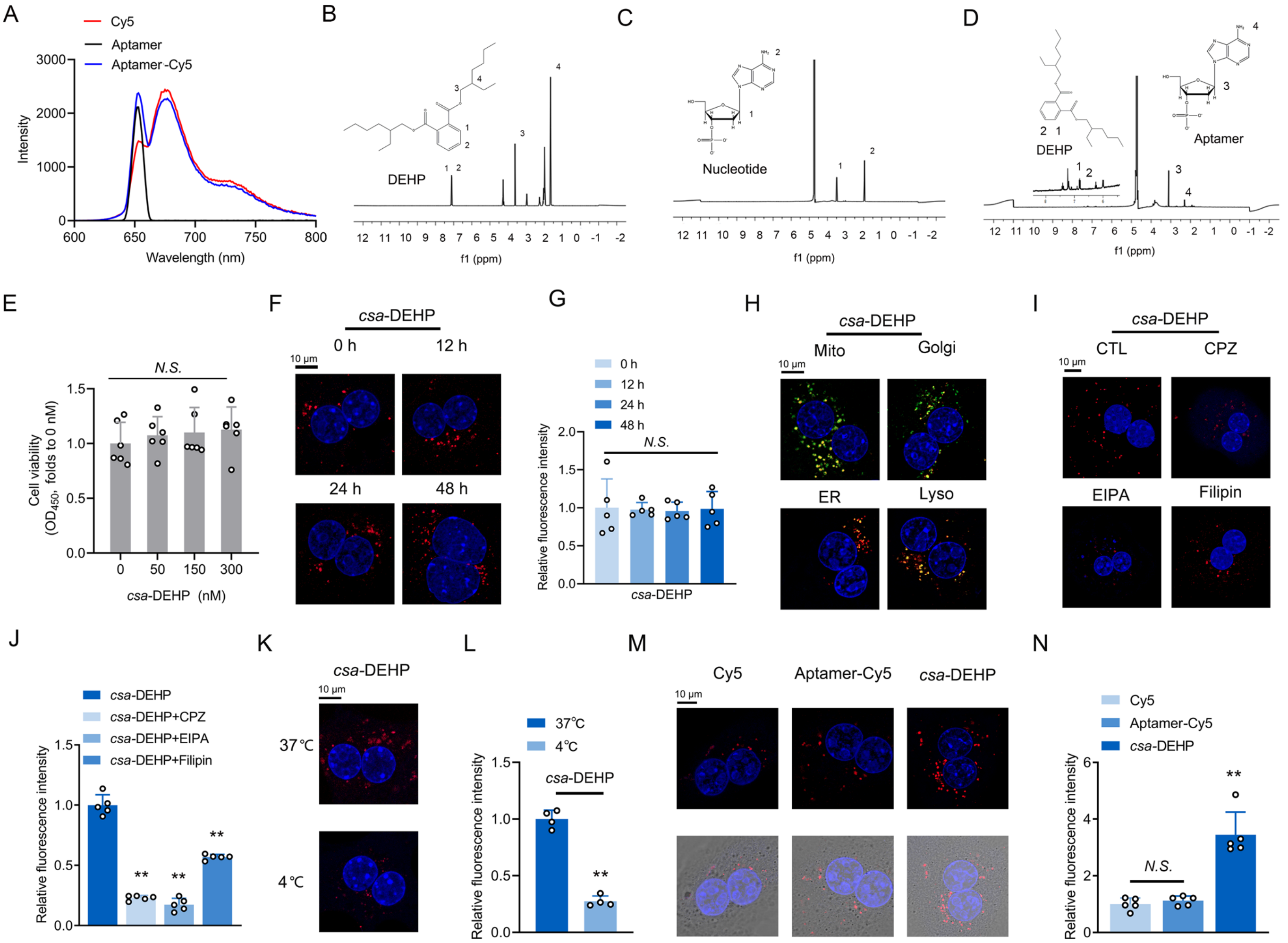

Figure EV3. Validation of DNA-aptamer targeting the DEHP.

(**A**) Fluorescent spectra of the Cy5, aptamer and aptamer-Cy5. (**B**) ^{1}H NMR spectra of DEHP. (**C**) ^{1}H NMR spectra of aptamer. (**D**) ^{1}H NMR spectra of aptamer-DEHP. (**E**) A CCK-8 analysis for cell toxicity of *csa*-DEHP in mouse PHs that were treated with indicated doses of *csa*-DEHP for 24 h. $n = 6$. *N.S.*, no significance. (**F**) CLMS analysis of retention time of internal *csa*-DEHP in mouse PHs following incubation with 150 nM *csa*-DEHP for 12 h. (**G**) Quantitative analysis of the relative fluorescence intensity in (**F**). *N.S.*, no significance. $n = 5$. '*n*' represents biological replicates. (**H**) Organelle localization of *csa*-DEHP in mouse PHs treated with 150 nM *csa*-DEHP for 12 h, and subsequently stained with specific organelle fluorescent trackers. (**I**) Representative CLMS images of mouse PHs treated with either *csa*-DEHP alone or in combination with indicated inhibitors. (**J**) Quantitative analysis of the relative fluorescence intensity in (**I**). $^{**}P < 0.01$ *vs*. *csa*-DEHP group, $n = 5$. '*n*' represents biological replicates. (**K**) Representative CLMS images of *csa*-DEHP in mouse PHs treated with 150 nM *csa*-DEHP for 1 h in either 37 °C or 4 °C. (**L**) Quantitative analysis of the relative fluorescence intensity in (**K**). $^{**}P < 0.01$ *vs*. 37 °C group, $n = 4$. '*n*' represents biological replicates. (**M**) Representative CLMS images of mouse PHs treated with 150 nM Cy5, Aptamer-Cy5 and *csa*-DEHP, respectively, for 12 h. (**N**) Quantitative analysis of the relative fluorescence intensity in (**M**). $^{**}P < 0.01$ *vs*. Cy5 group, $n = 5$. '*n*' represents biological replicates. Scale bar: 10 μm. Mito, Mitochondria. ER, Endoplasmic Reticulum. Golgi, Golgi apparatus. Lyso, Lysosome. Organelle, green. *csa*-DEHP, red. Nuclei, blue. All the data were represented as the mean ± SD. The paired Student's *t*-test was employed to compare between two groups. One-way ANOVA with a Fisher's LSD post hoc test was utilized to compare among multiple groups. Exact *P* values are listed in Appendix Table S11.

 © The Author(s)

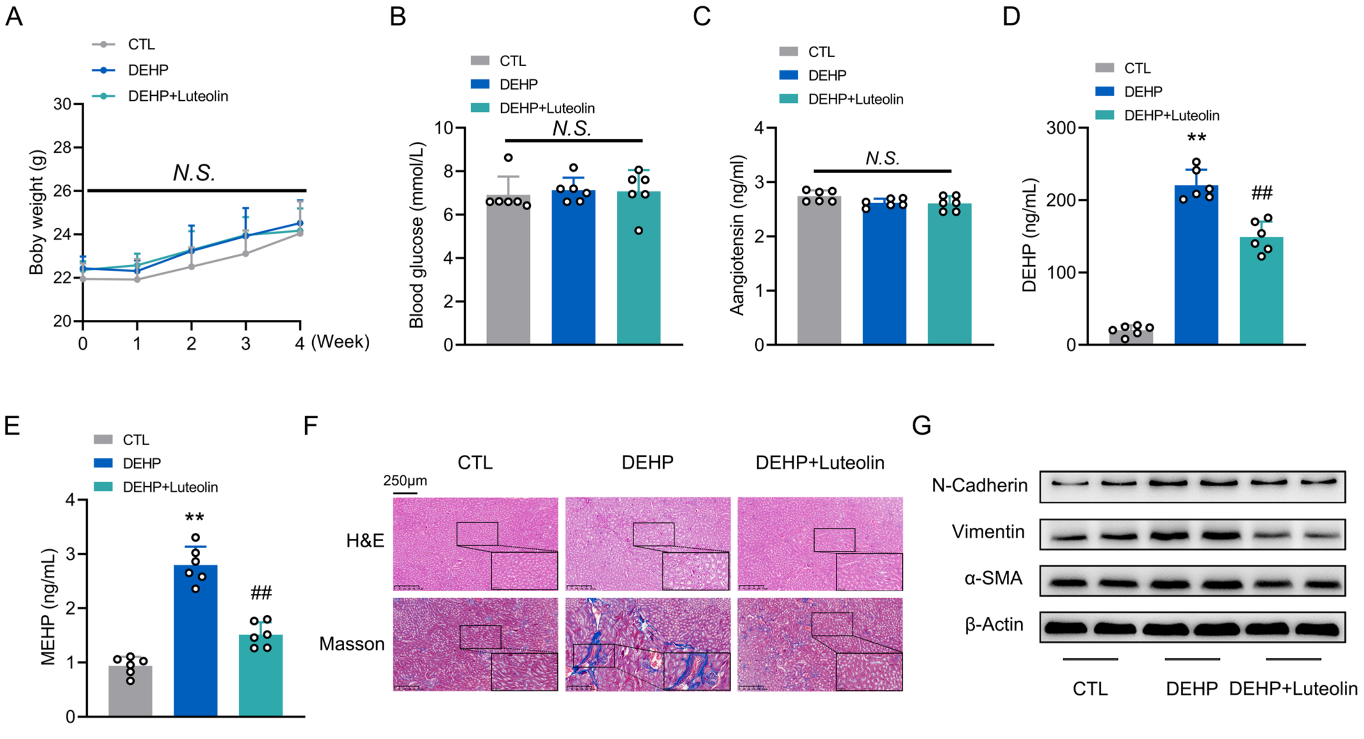

Figure EV4. The effects of luteolin on the metabolic profile of DEHP-treated mice.

Mice subjected to a 14-day treatment with DEHP (10 mg/kg body weight/day), followed by an additional 14-day treatment with luteolin (10 mg/kg body weight/day) in the presence of DEHP. $n = 6$. (**A**) Body weight. *N.S.*, no significance. (**B**) Serum levels of glucose. *N.S.*, no significance. (**C**) Serum levels of angiotensin. *N.S.*, no significance. (**D**) HPLC-MS analysis of DEHP levels in the adipose tissue of mice. $^{**}P < 0.01$ *vs.* CTL group. $^{\#\#}P < 0.01$ *vs.* DEHP group. $n = 6$. (**E**) HPLC-MS analysis of MEHP levels in the adipose tissue of mice. $^{**}P < 0.01$ *vs.* CTL group. $^{\#\#}P < 0.01$ *vs.* DEHP group. $n = 6$. (**F**) The renal histopathological examination and collagen deposition were performed by H&E (up) and Masson's trichrome staining (down), respectively. (**G**) Western blot analyses of EMT and fibrotic markers expression in the kidney of mice. All the data were represented as the mean ± SD. The paired Student's *t*-test was employed to compare between two groups. One-way ANOVA with a Fisher's LSD post hoc test was utilized to compare among multiple groups. Exact *P* values are listed in Appendix Table S11.

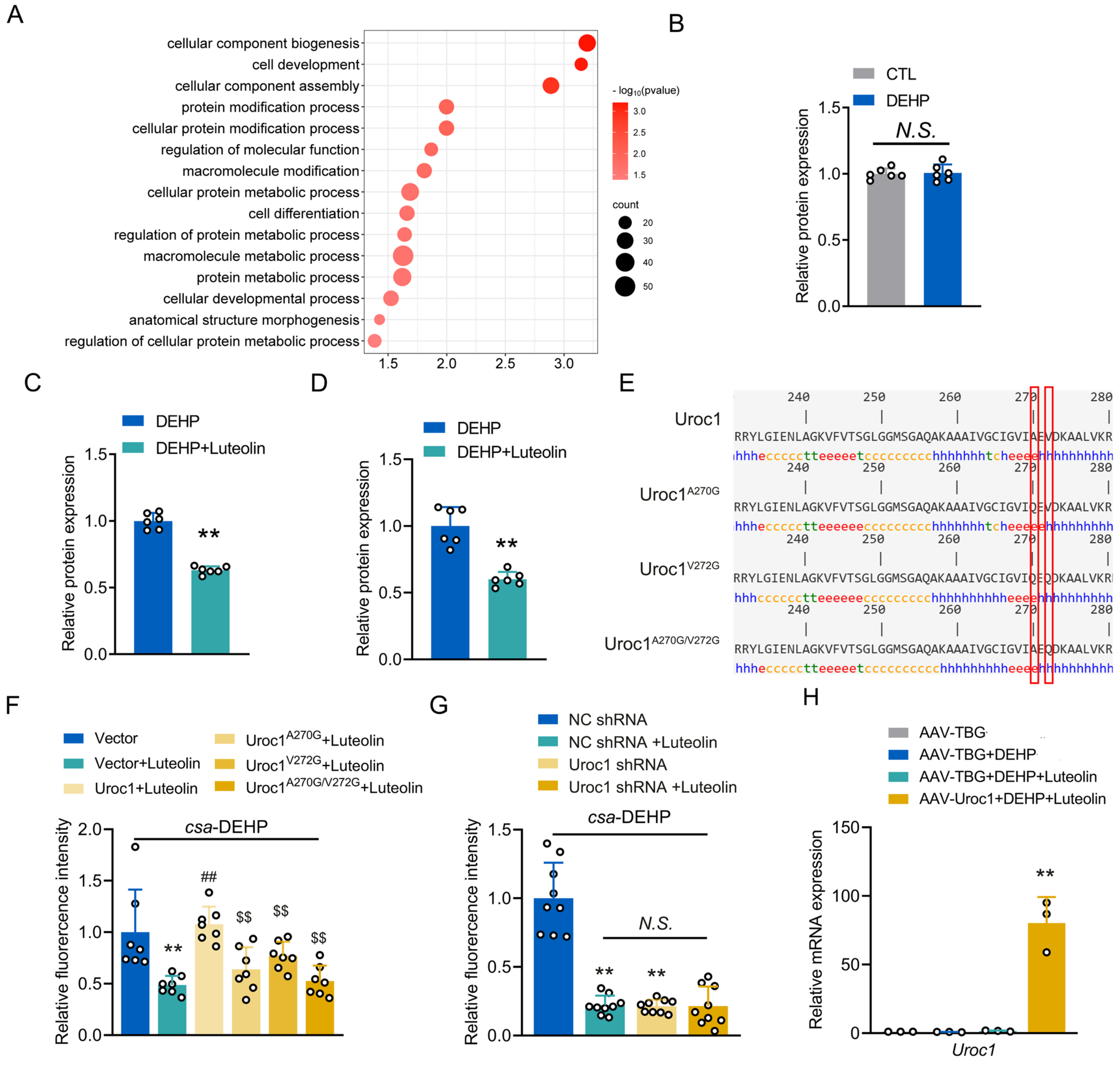

Figure EV5. Uroc1 serves as the molecular target of luteolin.

(**A**) GO enrichment analysis of differentially expressed proteins (biological process). The GO functional annotations of all differentially expressed proteins were compared with those of the reference species (or all experimentally identified proteins). A Fisher's Exact Test was performed to determine the significance of the differences and identify enriched functional categories (with a *P* value < 0.05). (**B**) Quantitative analysis of protein expression in Fig. 3F. (**C**) Quantitative analysis of protein expression in Fig. 3G. $^{**}P < 0.01$ *vs.* DEHP group. $n = 6$. (**D**) Quantitative analysis of protein expression in Fig. 3H. *N.S.*, no significance. $^{**}P < 0.01$ *vs.* DEHP group. $n = 6$. '*n*' represents biological replicates. (**E**) A cluster of three evolutionarily conserved, positively charged amino acids in Uroc1 were mutated to Gln. Secondary structures of the wild-type and mutated mouse Uroc1 fragments were predicted using the SOPMA algorithm. h, helix. e, sheet. t, turn. c, coil. (**F**) Quantitative analysis of the relative fluorescence intensity in Fig. 4D. $^{**}P < 0.01$ *vs.* Vector group, $^{\#\#}P < 0.01$ *vs.* Vector+Luteolin group, $^{\$\$}P < 0.01$ *vs.* Uroc1+Luteolin group. $n = 7$. '*n*' represents biological replicates. (**G**) Quantitative analysis of the relative fluorescence intensity in Fig. 4E. *N.S.*, no significance. $^{**}P < 0.01$ *vs.* NC shRNA group. $n = 9$. '*n*' represents biological replicates. (**H**) RT-qPCR analysis of Uroc1 expression in the liver of mice treated as described in Fig. 3H. $^{**}P < 0.01$ *vs.* AAV-TBG + DEHP+Luteolin group, $n = 3$. All the data were represented as the mean ± SD. The paired Student's *t*-test was employed to compare between two groups. One-way ANOVA with a Fisher's LSD post hoc test was utilized to compare among multiple groups. Exact *P* values are listed in Appendix Table S11.

 © The Author(s)
